# Autolougous platelet concentrates in esthetic medicine

**DOI:** 10.1111/prd.12582

**Published:** 2024-07-31

**Authors:** Catherine Davies, Richard J. Miron

**Affiliations:** ^1^ ZD Hair Clinic Johannesburg South Africa; ^2^ Advanced PRF Education Venice Florida USA; ^3^ Department of Periodontology University of Bern Bern Switzerland

**Keywords:** Alb‐PRF, Bio‐Filler, platelet concentrates, platelet‐rich fibrin, platelet‐rich plasma

## Abstract

This narrative review summarizes current knowledge on the use of autologous platelet concentrates (APCs) in esthetic medicine, with the goal of providing clinicians with reliable information for clinical practice. APCs contain platelets that release various growth factors with potential applications in facial and dermatologic treatments. This review examines several facial esthetic applications of APCs, including acne scarring, skin rejuvenation, melasma, vitiligo, stretchmarks, peri‐orbital rejuvenation, peri‐oral rejuvenation, hair regeneration and the volumizing effects of APC gels. A systematic review of literature databases (PubMed/MEDLINE) was conducted up to October 2023 to identify randomized controlled trials (RCTs) in the English language on APCs for facial rejuvenation and dermatology. A total of 96 articles were selected including those on platelet rich plasma (PRP), plasma‐rich in growth factors (PRGF), and platelet‐rich fibrin (PRF). Clinical recommendations gained from the reviews are provided. In summary, the use of APCs in facial esthetics is a promising yet relatively recent treatment approach. Overall, the majority of studies have focused on the use of PRP with positive outcomes. Only few studies have compared PRP versus PRF with all demonstrating superior outcomes using PRF. The existing studies have limitations including small sample sizes and lack of standardized assessment criteria. Future research should utilize well‐designed RCTs, incorporating appropriate controls, such as split‐face comparisons, and standardized protocols for APC usage, including optimal number of sessions, interval between sessions, and objective improvement scores. Nevertheless, the most recent formulations of platelet concentrates offer clinicians an ability to improve various clinical parameters and esthetic concerns.

## 1 FACIAL AGING

Facial aging is a complex process that affects everyone as they age. It is multifactorial process that results from both intrinsic and extrinsic factors.^1^, ^2^, ^3^ Intrinsic factors include genetics, hormonal changes, and cellular senescence, while extrinsic factors include sun exposure, smoking, pollution, and poor lifestyle habits. A decline in skin elasticity, loss of facial fat, and the appearance of wrinkles and fine lines characterizes the aging process. This article aims to provide a comprehensive review of the mechanisms and factors contributing to facial aging and potential treatment options.

### 1.1 Mechanisms of facial aging

The intrinsic factors reduce collagen and elastin production, cause loss of facial fat, and thinning of the epidermis.^4^, ^5^, ^6^ Hormonal changes, such as a decrease in estrogen levels in women, can further reduce collagen production and contribute to skin thinning.^6^, ^7^ Cellular senescence, the process of cellular aging and death, can lead to a decrease in the number of fibroblasts responsible for producing collagen and elastin.^2^

Extrinsic factors, such as sun exposure, smoking, and pollution, can accelerate aging by inducing oxidative stress and inflammation. Sun exposure is a significant contributor to facial aging, as ultraviolet radiation damages the skin's DNA, leading to the breakdown of collagen and elastin fibers. Smoking is also a significant contributor to facial aging, as it reduces blood flow to the skin and lessens the production of collagen and elastin, involves changes to fat, muscle, and bone, as well as changes in skin tone and texture.^8^ It is characterized by changes that occur in different parts of the face at various decades of life as an individual ages. These include deterioration of skin tone and texture, deflation of certain areas due to loss of bone and fat, and descent of soft tissues and fat due to loss of muscle tone and skin elasticity.

### 1.2 Facial anatomy

Facial anatomy continues to be one of the cornerstones of every facial esthetics procedure. To properly apply the numerous treatments outlined, it is necessary to comprehend and go over the anatomy, characteristics, and landmarks of the face. The layers that make up the face include the skin, connective tissue, subcutaneous fat layers, muscles, ligaments, and underlying bone. Numerous arteries, veins, and nerves may also be found inside this network. While they are reviewed extensively in various facial esthetics books,^8^ this article will provide a very brief overview.

**FIGURE 1:**
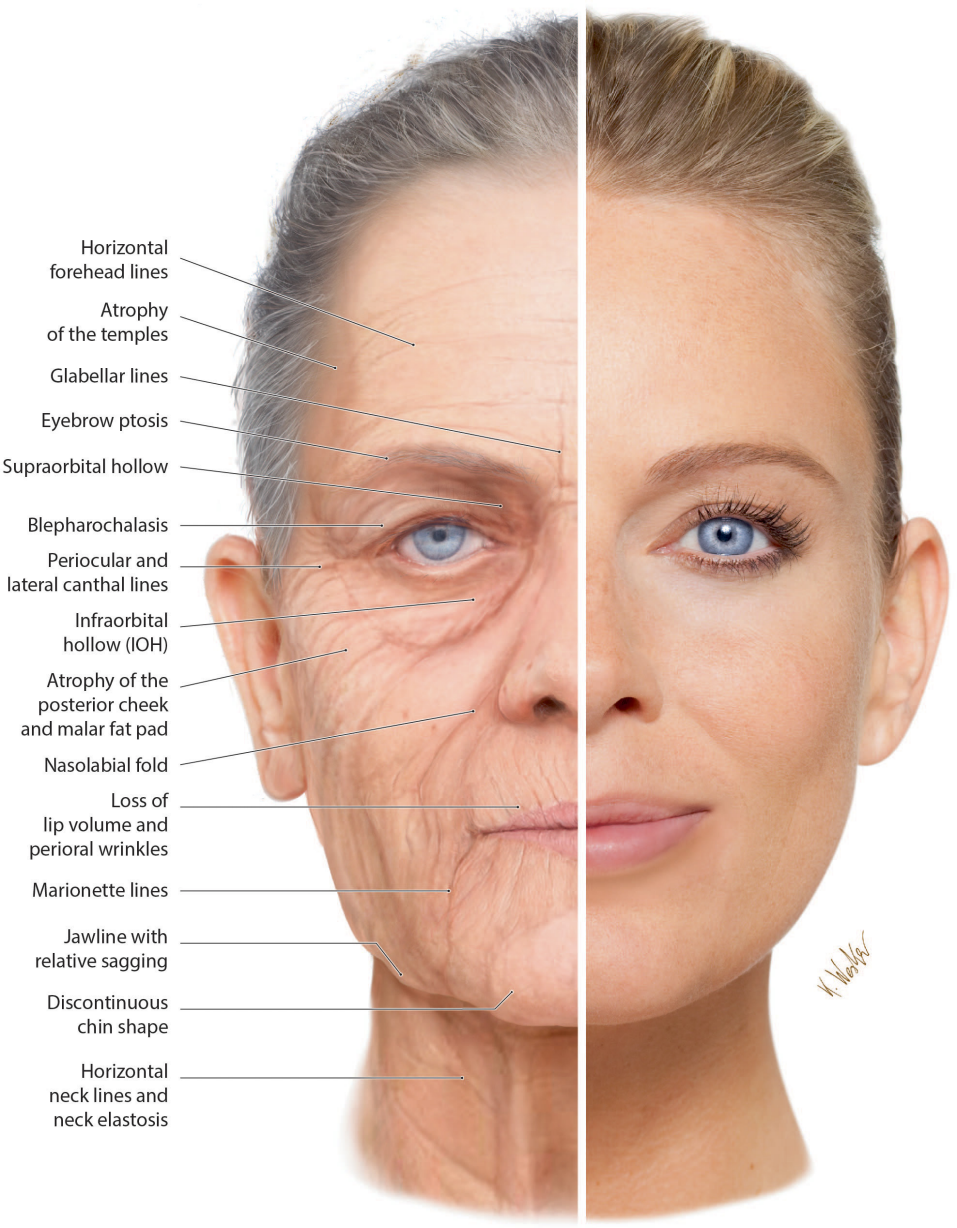
The process of aging skin. Notice that with age, facial features tend to sag with a volume shift downward of facial tissues. Reprinted with permission from Davies/Miron.^8^

While older people have sagging muscles and slack skin with fewer facial emotions, younger people have plumped muscles and firm skin and are able to completely express themselves during facial communication (Figure 1).^8^ Furthermore, the face is continuously exposed to external environmental factors such as exposure to the sun, smoking, and other chemicals. Because of these factors, a disproportionately significant segment of the esthetics market is dedicated to face‐specific skincare and cosmetics.

Therefore, it is crucial for the physician to have a good grasp of these facial traits, characteristics, and the underlying anatomy in order to slow down or even reverse the effects of aging. As a result of the typically reduced treatment costs (compared to, say, the 90s), more people are able to afford the desire to acquire a renewed appearance, which has contributed to its rising popularity.

#### 1.2.1 Muscles of the face

The face consists of a total of 30 different muscles. These are typically divided via three muscle planes and are thus distinguished as (1) superficial, (2) middle, and (3) deep.^8^ As dynamic co‐players in soft tissue complexes, muscles play an extremely important role in facial aging. Muscles are important to understand to better address dynamic wrinkles caused during contraction. Naturally, with age, these muscles become hypertrophic, permanently causing visible wrinkles that are involuntary and undesirable.

#### 1.2.2 Subcutaneous fat and connective tissue

**FIGURE 2:**
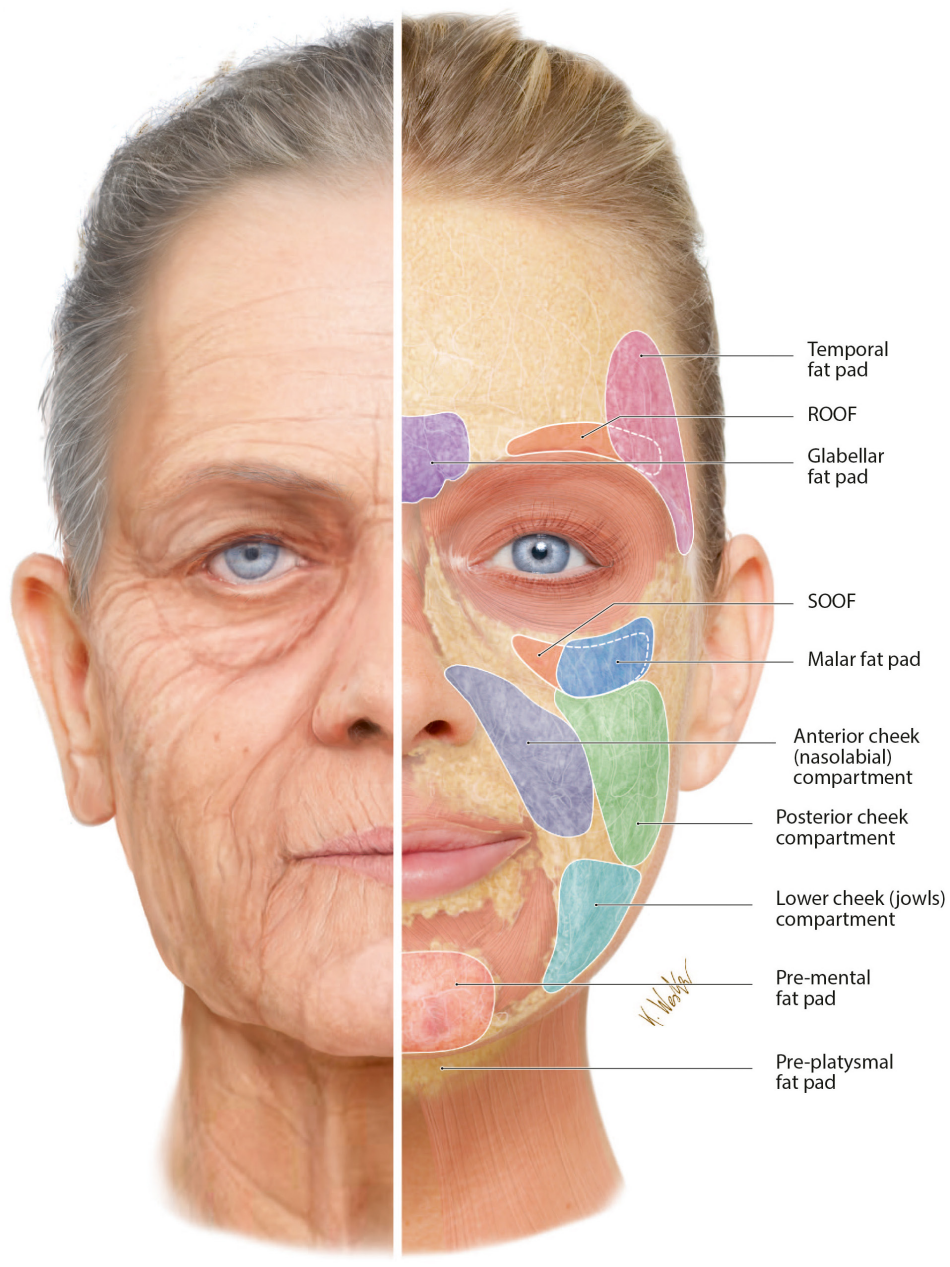
Split view of the clinical signs of aging and subcutaneous fat distribution of the face. It is apparent at first glance that there is a correlation between them. At sites where superficial fat is absent, alongside facial atrophy due to deep fat loss, the clinical signs of aging become apparent at a particularly early age. Sites of fat loss around the eyes and mouth are therefore considered to be facial aging “hot spots.” Reprinted with permission from Davies/Miron.^8^

The subcutaneous fat acts as a “volumizing cushion” for the face's soft tissues by integrating into the facial connective tissue. In addition to playing a significant function in shielding the face from harm from the outside, it makes sure that the facial tissues are constantly receiving vital nutrients and fluids. High‐fat compartments on the face are usually well‐defined and uniform in layers. These include the glabella, the jaw‐chin area, the cheeks, and the nasolabial folds (Figure 2). This particular tissue shrinks with age in elderly people, usually due to decreased blood supply, causing atrophy. Notice also in Figure 2 that a very thin subcutaneous fat layer exists in the area of the temples and forehead, and almost none exists in the periorbital and perioral region. As a result, these regions are more likely to develop creases and folds with age and are among the first indications of face aging in people.

#### 1.2.3 Blood supply

**FIGURE 3:**
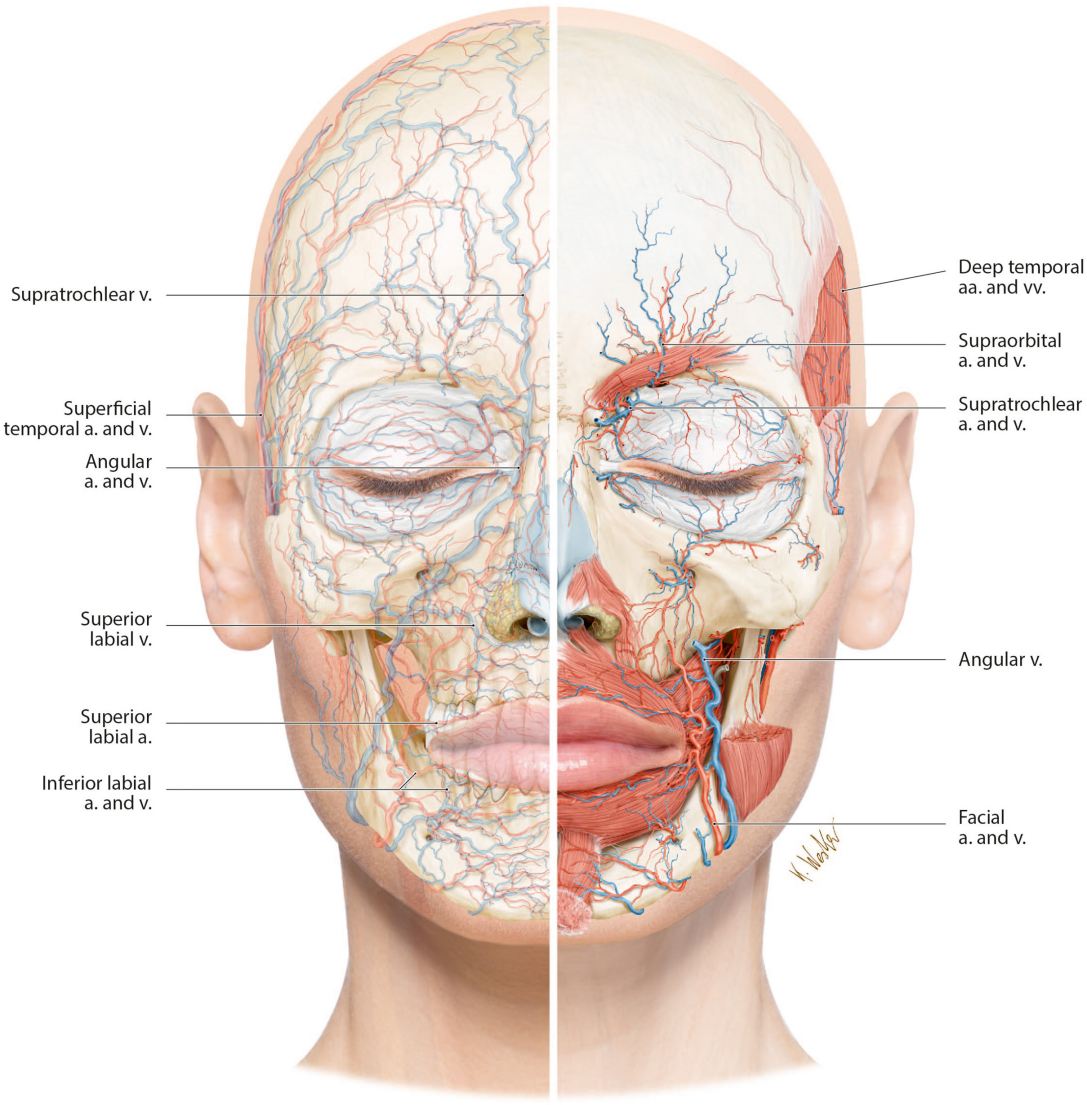
Blood vessels of the face projected onto the facial skeleton (left) and the position of the deep arteries and veins of the face relative to the deep muscles (right) (a., artery; aa., arteries; v., vein; vv., veins). Reprinted with permission from Davies/Miron.^8^

There is a noticeable and intricate blood vessel network almost everywhere on the face (Figure 3). Fine capillary capillaries deliver blood to the skin's periphery layers. Adequate diffusion is made possible by these tiny veins into every blood layer. When injecting into areas of the face, *a thorough knowledge regarding the whereabouts of the major blood vessels is crucial*. By doing so, the occurrence of possible problems associated with intravascular injections, which are most often observed with fillers, would be prevented. Significantly, understanding the structure of the face, arteries, and veins is crucial.

#### 1.2.4 Innervation

In addition to the blood supply, the face is also equipped with a sophisticated innervation system that primarily originates from 2 sources: the trigeminal nerve and the facial nerve. The trigeminal nerve supplies the sensory innervation of the face. The nerve in question is comprised of three branches, one of which is the V1 ophthalmic nerve. This particular branch exits the orbit via the supraorbital foramen and fissure and is responsible for providing sensory input to the top region of the face. The V2 maxillary nerve originates from the infraorbital foramen and provides innervation to the midface. The V3 mandibular nerve innervates the mandibular and temporal regions (reviewed in great detail in the textbook by Davies and Miron, 2020).^8^

### 1.3 Biology of the skin

**FIGURE 4:**
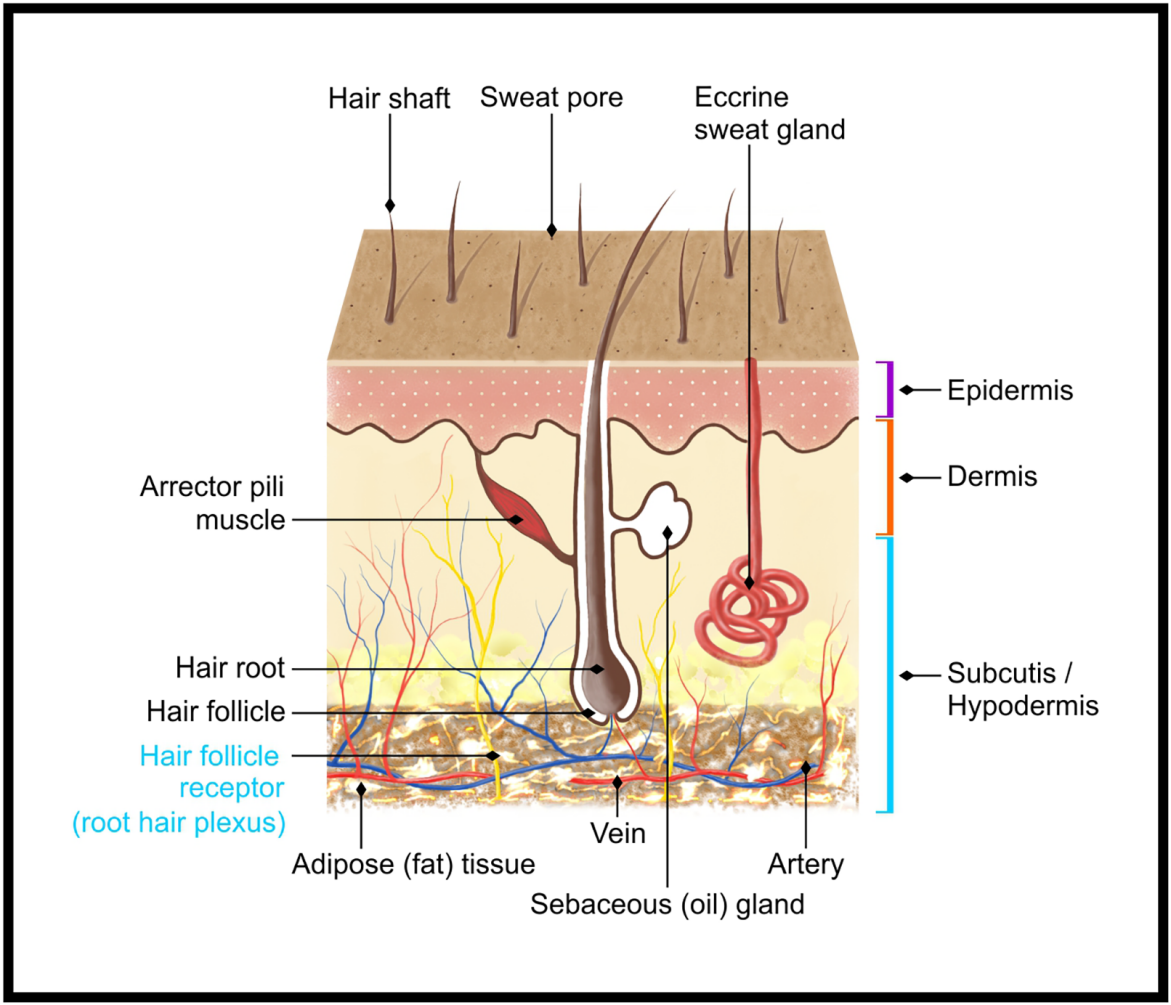
Illustration of the various layers of skin. Reprinted with permission from Davies/Miron.^8^

Prior to commencing any facial esthetic regimen, it is also important to have a thorough understanding of the various layers and cell types found in the skin (Figure 4). Within each section, the structure and function of each layer are described with an overview of each of the individual roles of the various skin/hair layers. The importance of vascularization within these tissues is clearly defined providing fundamental principles and reasoning for the use of platelet concentrates presented in this series of articles.

The skin, being the body's largest organ, is vital for maintaining human health. Its core function is to provide a protective barrier and waterproof sheath for the body by encasing the body's organs. The skin performs many vital functions, such as providing a protective barrier from UV light, water loss, temperature regulation, microbes, and chemicals. The skin is composed of three layers: the epidermis, the dermis, and subcutaneous tissue.

#### 1.3.1 The epidermis

The epidermis is the outermost layer of the skin. It is multi‐layered and made up mainly of keratinocytes. It is generally considered to be subdivided into 4 or 5 separate strata as follows^8^:

**Table:** 

**Stratum basale** is also known as the stratum germinativum; this is the deepest layer, joined by hemidesmosomes and divided from the dermis by the foundation membrane cuboidal to columnar stem cells, which are mitotically active. **Stratum spinosum**—The stratum spinosum, also known as the prickle cell layer, is composed of irregular, polyhedral cells that possess processes extending outward and establishing contact with adjacent cells by desmosomes. **Stratum granulosum**—The cells in question are diamond‐shaped and possess keratohyalin granules. **Stratum lucidum**—The stratum lucidum is only found in thick skin and is a thin, transparent layer composed of eleidin, which is a transformed derivative of keratohyalin. **Stratum corneum**—The outermost layer consists of keratin and horny scales, which were formerly live cells. These cells, now dead, are referred to as squamous cells.

The epidermis has four major cell types: Merkel cells, Langerhans cells, melanocytes, and keratinocytes. Keratinocytes, which comprise 95% of epidermal cells, are the major cell type of the epidermis. The primary structural proteins of the stratum corneum are produced by keratinocytes. The pigment‐producing epidermal cells known as melanocytes are located in the basal layer of the skin and provide some UV protection. Dendritic immune cells, known as Langerhans cells, are dispersed throughout the suprabasal area of the epidermis.

#### 1.3.2 The dermis

The dermis is located beneath the epidermis and is between 1.5 and 4 mm thick. It is the thickest of the three layers and makes up approximately 90% of the thickness of the skin. The main functions of the dermis are to supply the epidermis with nutrients, regulate temperature, and store much of the body's water supply. It is made up of 2 layers, including the stratum papillare and stratum reticulare (Figure 4).

**Table:** 

**Blood vessels**—The skin receives vital nutrients and oxygen from the blood arteries, which also remove waste. Additionally, the blood arteries carry the vitamin D generated by the skin back to the body's other organs. **Lymph vessels**—All of the skin's tissues get lymph from the lymph vessels. As lymph flows to the lymph nodes, these cells function to eradicate any illness or foreign germs. **Hair follicles**—The sheath that covers and feeds the portion of hair under the epidermis is called a hair follicle. **Sweat glands**—There are two different kinds of sweat glands in the dermis; Apocrine glands are located only in the pubic area and armpits. Sweat is produced by these glands. The actual sweat glands, or eccrine glands, are located throughout the body's dermis. These glands' main job is to control body temperature by delivering a hypotonic solution via pores to the skin's surface, where it evaporates to lower body temperature while preserving sodium. **Sebaceous glands**—The majority of sebaceous, or oil, glands are located on the face and scalp and are connected to hair follicles. Sebum, or oil, secreted by these glands keeps the skin supple and smooth. **Nerve endings**—Additionally, the dermis layer has touch and pain receptors that provide pressure, pain, itching, and temperature data to the brain for interpretation.

#### 1.3.3 The subcutaneous tissue

The subcutaneous tissue, also known as the hypodermis, is the deepest skin layer, varying in thickness from a few mm to several centimeters. It is made of fat, divided by loose connective tissue into fat clusters, and is separated from the underlying tissues by fascia.

### 1.4 The effects of facial aging

Today, facial appearance is one of the most important factors influencing our perception of beauty and attractiveness.^9^ The effects of facial aging are visible in several ways (Figure 1, Table 1).^8^ These include the appearance of fine lines and wrinkles, sagging skin, and loss of facial volume.^10^ Fine lines and wrinkles result from the loss of collagen and elastin fibers in the skin, leading to a reduction in skin elasticity. On the other hand, sagging skin results from a loss of facial fat and the weakening of the muscles that support the skin. The loss of facial volume, commonly seen in the cheeks and under the eyes, can result in a sunken appearance.^10^, ^11^

**TABLE 1:** In normal aging, the following changes are expected as progression occurs^8^:

Corners of the mouth move inferiorly resulting in a slight frown look
Tissue around the eyes sag inferiorly
Eyelids (upper and lower) sag inferiorly
Tissue of the forehead drifts inferiorly, creating wrinkles and dropping the eyebrows downward with flatter appearances
Nose may elongate and the tip may regress inferiorly
Nose may develop a small to pronounced dorsal hump
Tip of the nose may enlarge and become bulbous
Generalized wrinkling to the face naturally occurs
Inversion of the youthful upside‐down triangle of the face
Skin discoloration (dark circles, superficial capillaries, pigmentary disorders)
Loss of proportion of the skin envelope (loss of subcutaneous fat, downward sagging of the soft tissues)
Glabellar lines
Sagging of the eyebrows (ptsosis)
Sunken eyes (Supraorbital hollowness)
Infraorbital hollowness (dark circles under the eyes, tear trough deformity)
Fat atrophy in the upper check region (malar fat pad)
Deep nasolabial folds
Wrinkles around the mouth aka smoker's lines
Loss of lip volume and perioral wrinkles
Drooping corners of the mouth and jowls (marionette lines)
Irregular chin contour, dimpling and “sagging”

Initially, these changes occur on the anatomical and cellular levels below the skin surface (fat tissue, muscles, and bones). Eventually, they become apparent on the skin. One of the early signs of aging is found in sites with little to no superficial fat layers. When developing strategies for facial rejuvenation procedures, it is important to understand the anatomy and the mechanism of tissue breakdown. The treating clinician may begin to ask himself/herself some questions. Was the skin damage caused by UV with resulting loss of collagen synthesis? Was it caused by smoking affecting blood flow? Are wrinkles and facial folds caused by hyperactive muscles? These are all important questions to ask as a practitioner in order to develop and recommend effective therapeutic strategies.

Age‐related changes in facial tissues most often alter blood supply, and as a result, atrophy‐related deterioration is observed.^8^ This markedly decreases fat tissue layers, rate of cell division of skin cells, and collagen synthesis. Each of the above‐mentioned scenarios also impairs the regeneration capacity of various tissue‐types and also impairs the natural barrier function of the skin. Noteworthy, skin hydration is also affected by aging, leading to further signs of facial aging.^8^

Many of the signs of aging are found in “hot spot” areas of the face, leading to changes in the topographical compassion of sites with subcutaneous fat distribution versus those without. The regions with low fat (around the eyes and around the lips) are more frequently clinically related to visible signs of aging. Therefore, the periorbital and perioral regions are starting points during facial regenerative/rejuvenating strategies. Always remember, the visible signs that are observed externally of the skin (wrinkles, skin laxity, and folds) are almost always related to an underlying cause at a deeper tissue level that is not clinically visible.

Furthermore, deep fat atrophy is a significant age‐related factor for skin aging and is primarily caused by a decrease in age‐related blood flow.^8^ Hence, vascular degeneration is considered a major cause of the initiation of facial aging, and hence, platelet therapies such as PRF have been deemed extremely effective strategies for minimizing further facial aging and potentially reversing it. Noteworthy, a decrease in blood flow caused by aging always results in a decrease in the supply of oxygen and nutrients to facial tissues, and shrinkage of deep fat stores undergo significant atrophy as a result. This gradual loss of fat volume from underlying subcutaneous tissues results in a decrease in skin tone and fluid levels in the facial tissue complex. Furthermore, it is one of the main reasons why fat grafting has been commonly utilized as a strategy in facial esthetics.^8^

## 2 GROWTH OF FACIAL ESTHETIC TREATMENTS

The global noninvasive esthetic treatment market size was valued at USD 61.2 billion in 2022 and is projected to expand at a compound annual growth rate of 15.4% from 2023 to 2030.^8^, ^12^ In comparison, the market for noninvasive facial esthetic treatments is currently over 10 times larger than the dental implant market (~6 billion) and is over double the size of all fields of dentistry combined (34 billion) growing at 5.5% per year.^8^ Thus, tremendous opportunities exist for clinicians aiming to perform such procedures and opens opportunities for medical professionals including dentists who are some of the world experts in head and neck anatomy, and also in performing injections. Noteworthy, various facial esthetics attractiveness studies have reported that a person's smile and teeth are part of the top 5 most important features towards facial attractiveness.^13^, ^14^ The ability for the dentist to treat both the smile/teeth and facial aging via noninvasive treatment options poses great opportunities.

Noninvasive skin rejuvenation treatments have increased significantly over the years due to an aging population, as well as an increasing focus on physical appearance among millennials and the Gen Z population. Various opportunities exist including the use of botulinum toxins (Botox) as well as hyaluronic acid facial fillers (Juvederm, Restylane). Noteworthily, however, the use of autologous platelet concentrates (APCs) offers patients much more natural treatment options, that are both safer and more natural looking. Furthermore they may be combined with lasers to offer patients all‐natural therapeutic options.^15^, ^16^

## 3 PLATELET CONCENTRATES (APCs) IN FACIAL ESTHETICS

APCs are increasingly used in facial esthetics for their regenerative and healing properties.^8^ These concentrates, also known as platelet‐rich plasma (PRP) and platelet‐rich fibrin (PRF) are derived from autologous plasma and used in various facial esthetic procedures. One of the main drivers of the growth of platelet concentrates in facial esthetics is the increasing demand for minimally invasive procedures. Patients are seeking treatments that provide natural and subtle results without the need for surgery or extended downtime. APCs offer these benefits, making them popular among patients. Furthermore and more recently, the ability to extend the working properties and the long‐lasting effects of APCs has been improved by the novel development of heating plasma and creating an “albumin gel” that lasts 4–6 months when injected all while simultaneously building collagen over time.^17^, ^18^, ^19^

Advancements in technology have also played a significant role in the growth of platelet concentrates in facial esthetics. The development of new methods for extracting and processing platelets and the use of growth factors and stem cells have improved the effectiveness of these concentrates in promoting skin rejuvenation and healing. Another factor contributing to the growth of platelet concentrates in facial esthetics is the increasing awareness of the benefits of these concentrates from social media and celebrity endorsement. Platelet concentrates are known for their regenerative properties, promoting collagen production, improving skin texture, and reducing the appearance of fine lines and wrinkles. APCs are used as a standalone therapy administered by injection or micro‐needling, as a volumizing agent, or as an adjuvant approach in combination with other esthetic treatments.

Esthetic medicine has recently witnessed a proliferation in the number of injectable platelet concentrate products containing supra‐physiological quantities of platelets and autologous growth factors to stimulate tissue repair and skin rejuvenation. This trend is expected to continue.^12^ Growth factors within these plasma concentrates have emerged as a promising therapeutic modality by regulating essential processes in skin rejuvenation, including angiogenesis, cell migration, cell proliferation, and collagen deposition.^20^, ^21^

## 4 BASIC RESEARCH: COMPARING PRP To PRF IN FACIAL ESTHETICS AND SKIN FIBROBLASTS

Several studies have investigated the use of APCs on facial skin fibroblasts. A paper by Kim et al.^22^ found that PRP stimulated cell proliferation, expression of type I collagen, MMP‐1 protein, and mRNA in human dermal fibroblasts.

**FIGURE 5:**
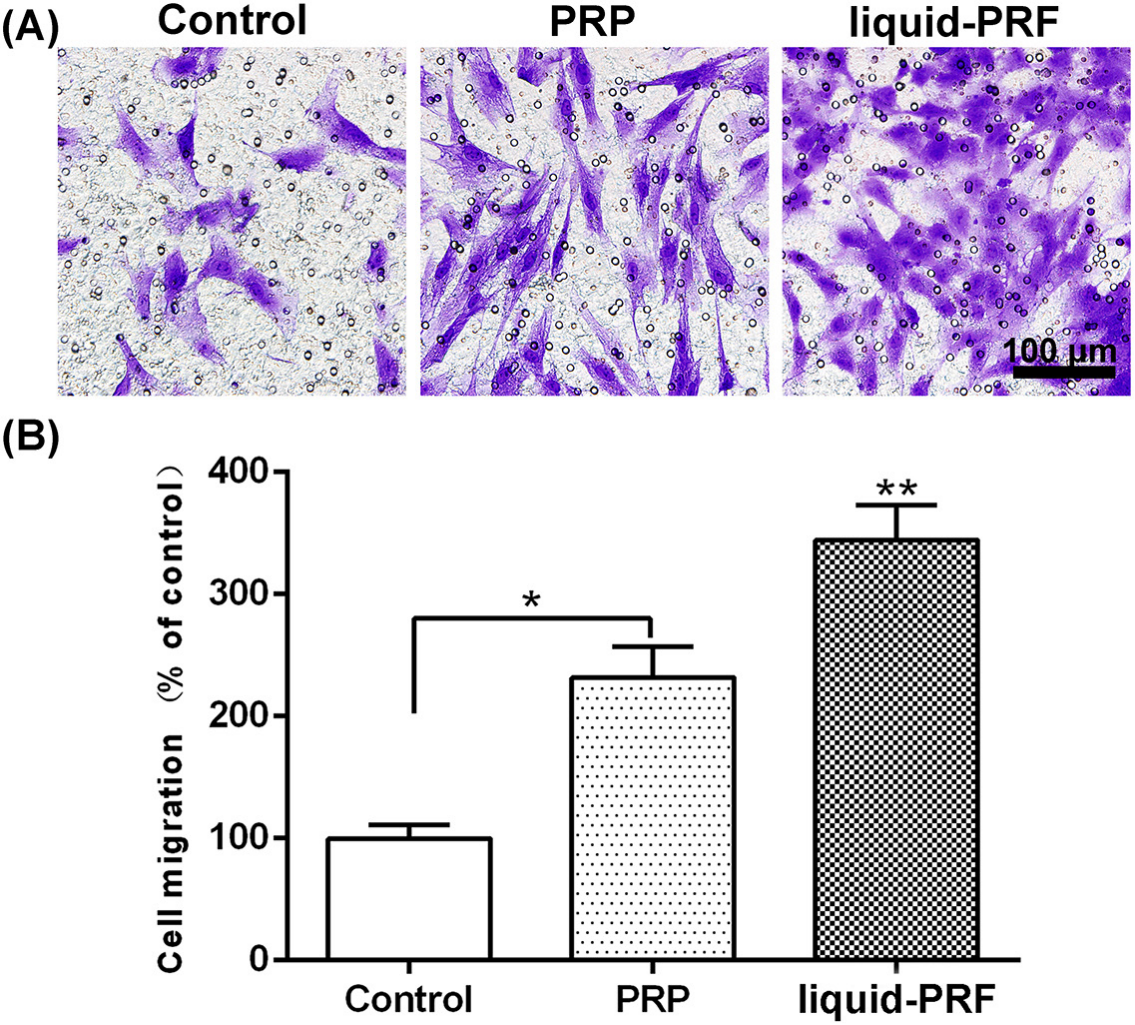
(A, B) Migration assay of human skin fibroblasts cultured with fluid‐PRF and PRP after 24 h. (Scale bar = 100 μm) (* denotes significant difference between two groups *p* < 0.05, ** denotes significantly higher than all other treatment groups *p* < 0.05). Assay performed in triplicate with three independent experiments. Reprinted with permission from Wang et al.^21^

**FIGURE 6:**
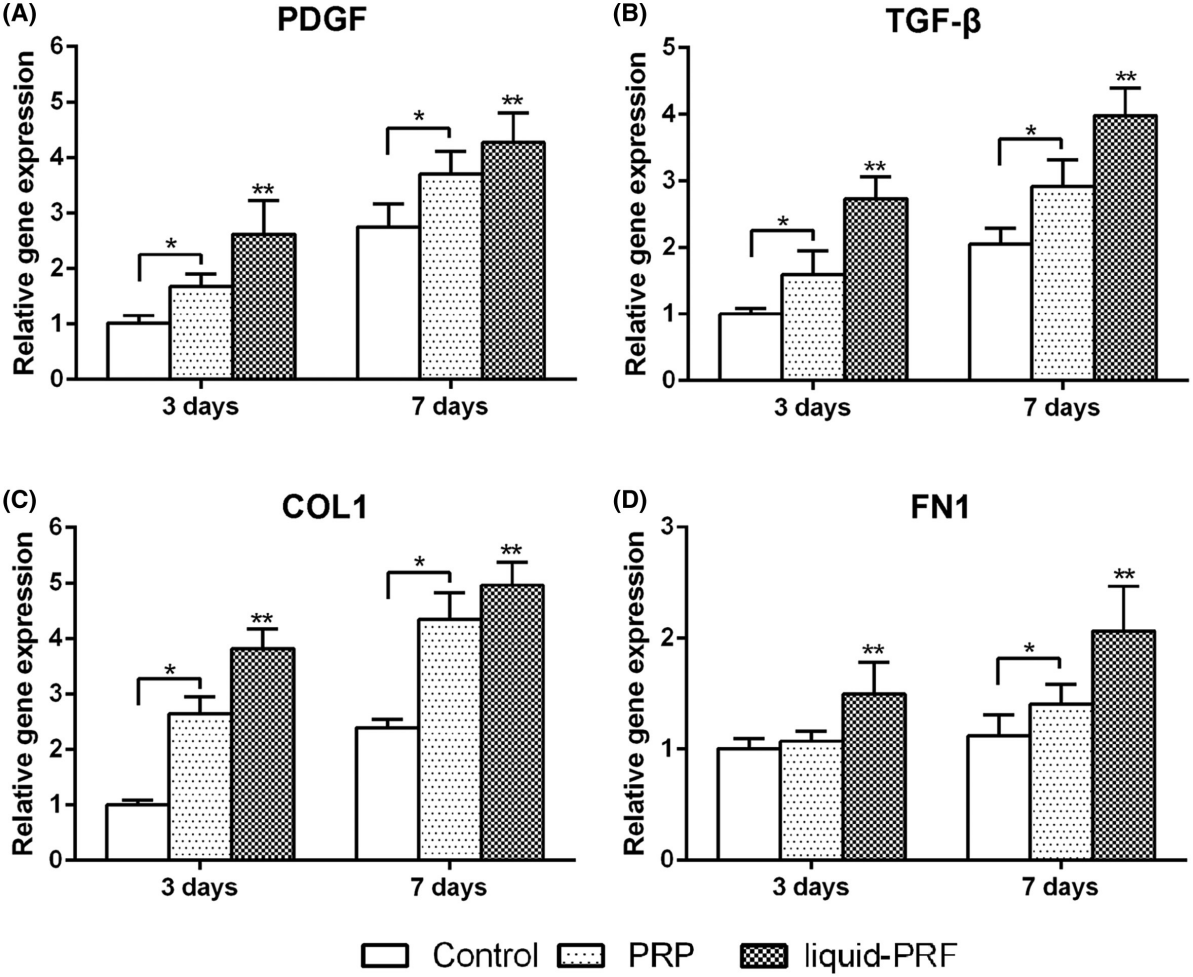
Expression of regeneration‐related and ECM‐related genes of gingival fibroblasts cultured with PRP and fluid‐PRF at 3 and 7 (A) PDGF; (B) TGF‐β; (C) COL1 and (D) FN1. (* denotes significant difference between two groups *p* < 0.05, ** denotes significantly higher than all other treatment groups *p* < 0.05). Assay performed in triplicate with 3 independent experiments. Reprinted with permission from Wang et al.^21^

**FIGURE 7:**
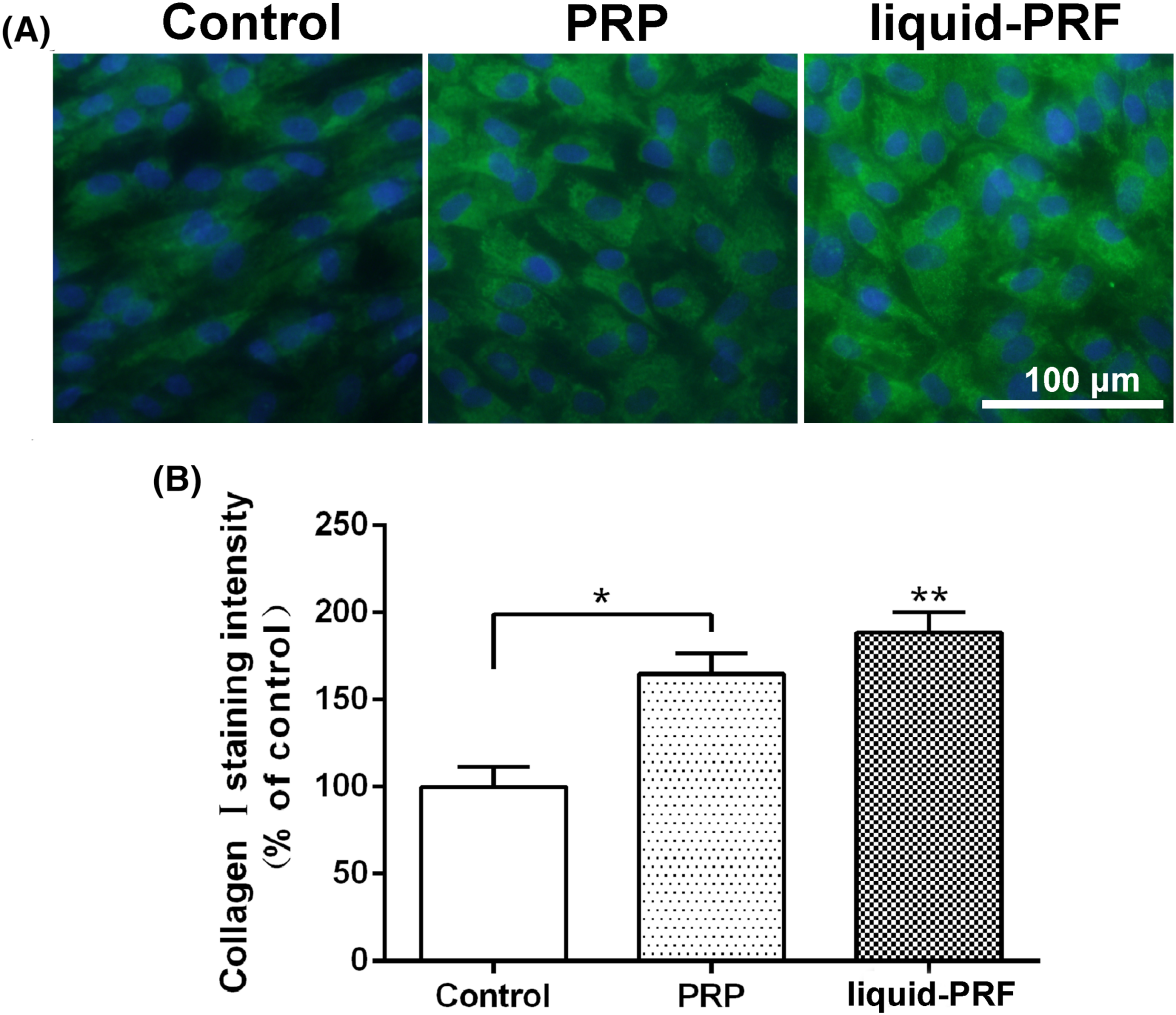
Immunofluorescent Collagen type 1 (COL1) staining of skin fibroblasts cultured with PRP and fluid‐PRF at 7 days. (A) COL1 staining (green) merged with DAPI staining (blue). (Scale bars = 100 μm); (B) COL1 staining quantification (* denotes significant difference between two groups *p* < 0.05, ** denotes significantly higher than all other treatment groups *p* < 0.05). Assay performed in triplicate with 3 independent experiments. Reprinted with permission from Wang et al.^21^

A study comparing liquid‐PRF to PRP on skin cell behavior and regeneration was conducted in 2019 by Wang et al.^21^ The capacity of dermal skin fibroblasts, cultivated with either fluid‐PRF or PRP, to affect or boost cell survival, migration, spreading, proliferation, and mRNA levels of recognized mediators of dermal biology, such as fibronectin, TGF‐beta, and PDGF, was examined in this work. Every platelet concentration showed good cell survival and was nontoxic to cells. In liquid‐PRF, skin fibroblast migration increased by nearly 350% as compared to control and PRP (200%) (Figure 5). At 5 days, liquid‐PRF also markedly increased cell proliferation. Although PDGF cell mRNA levels were dramatically raised by both PRP and liquid‐PRF, TGF‐beta, collagen1, and fibronectin mRNA levels were found to be much higher in the fluid‐PRF group (Figure 6). Finally, compared to PRP, liquid‐PRF showed a much higher capacity to stimulate collagen matrix formation (Figure 7). In conclusion, it was found that greater regenerative potential of liquid‐PRF on human skin fibroblasts.^21^ Furthermore, since PRF tubes do not contain any additives, they are considered a more natural approach to tissue regeneration (less expensive as well for the clinician).

## 5 ADMINISTRATION OF APCs

APCs may be administered topically in a gel form such as treatment of open wounds, burns or post laser wounds. In the field of facial esthetics APCs are most commonly administered by microneedling, intradermal injections or both routes.

### 5.1 Microneedling with APCs

**FIGURE 8:**
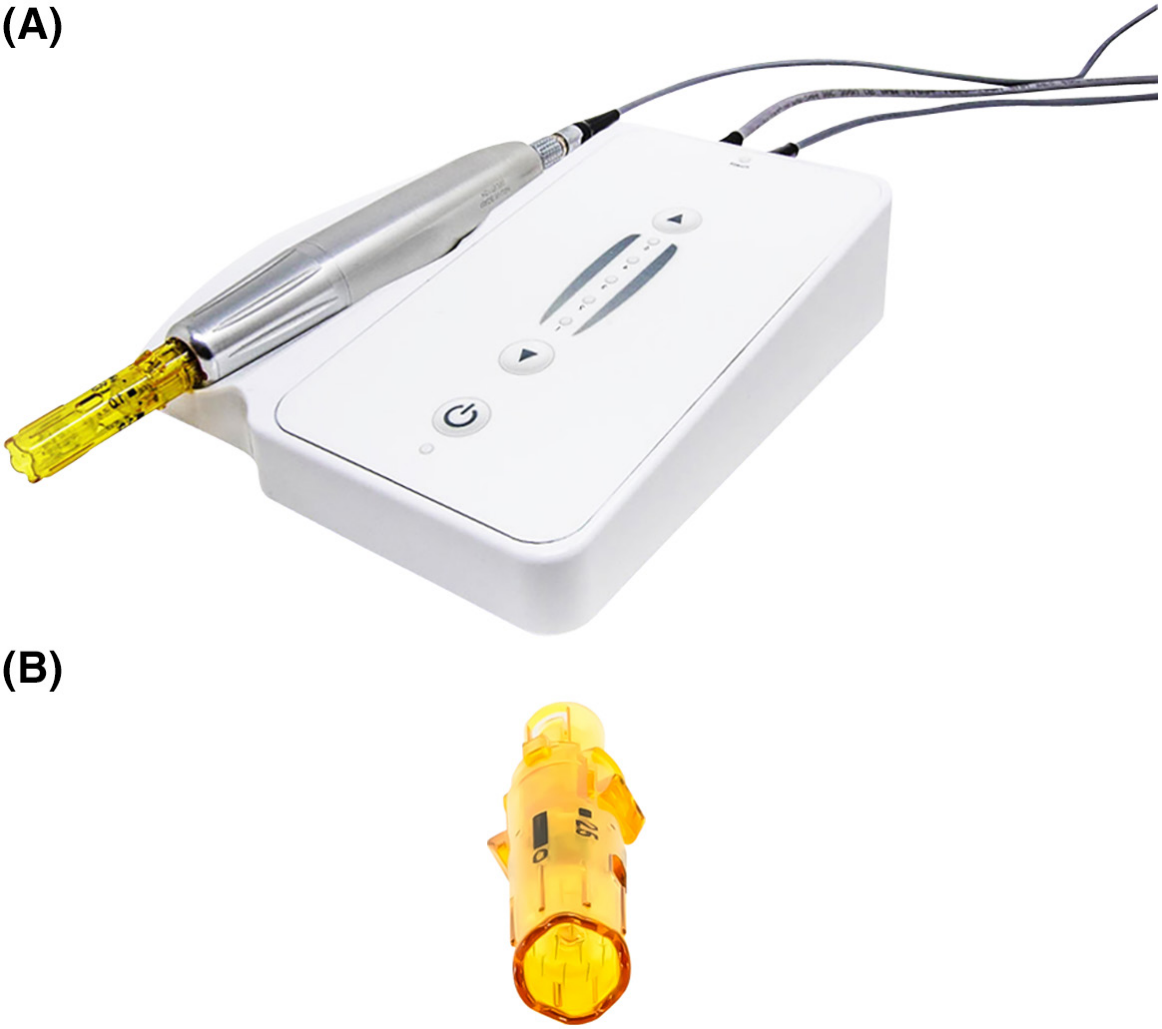
(A) Illustration of the DermaPen micro‐needling device. (B) Illustration of the DermaPen micro‐needling tip. Note that 12 small micro‐needles exist in such a device which repeated penetrate within 0.25 to 2.5 mm in depth within facial tissues at roughly 3–5000 RPMs. Reprinted with permission from Davies/Miron.^8^

**FIGURE 9:**
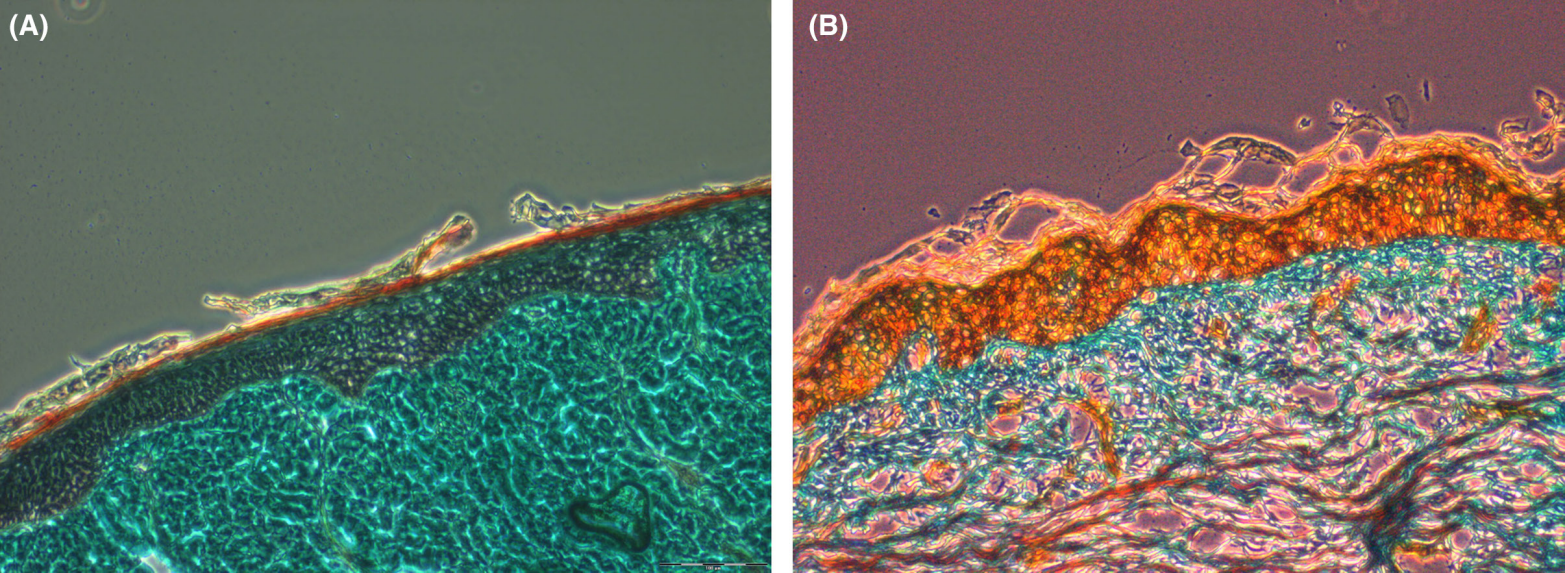
Masson's Trichrome staining. (A) Preoperative histologic photomicrograph of the burn scar. (B) Histologic photomicrograph of the burn scar obtained 24 months postoperatively. Van Gieson staining showed a considerable normalization of the collagen/elastin matrix in the reticular dermis and an increase in collagen deposition at 24 months postoperatively, and the collagen appears not to have been laid down in parallel bundles but is rather in the normal lattice pattern. Reprinted with permission from Aust et al.^24^

Microneedling, also known as “minimally invasive percutaneous collagen induction,” is perhaps one of the simplest and safest ways to deliver APCs in facial esthetics. It uses a number of “microneedles” (typically 12) to treat facial tissues in a minimally invasive, nonsurgical, and nonablative manner (Figure 8).^23^ The treatment is based on the principle of neovascularization, which occurs after minimal trauma and induces rapid neocollagenesis and tissue repair. Histological studies have demonstrated improvements in skin thickeness and hydration with microneedling alone (Figure 9).^24^ When combined with APCs, micro‐needling is a simple and effective procedure that offers clinical benefits via a safe treatment modality.^25^, ^26^, ^27^, ^28^

Microneedling can be performed in an automated way using an electrically powered hand‐held micro‐needling device that delivers a vibrating, stamp‐like motion to the skin, resulting in a series of micro‐channels. Considered a medical device, this microneedler is spring‐loaded with an adjustment ring that enables height alterations of the microneedles to penetration depths between 0.25 and 2.5 mm and in three directions. The resulting microchannels are then filled with APC; the device may also be utilized to “push” a substance (i.e., APC) into the skin to specific depths to facilitate facial rejuvenation via autologous growth factor release.

The advantages of micro‐needling are that it is an extremely safe skin resurfacing therapy and results in minimal damage to the skin. The down time is usually approximately 24–48 h. This method of facial rejuvenation has a much shorter downtime when compared to other comparable methods and lower risk of side effects such as hyperpigmentation and scarring (when compared to lasers for instance), making it a more ideal treatment choice for all individuals and especially those with thin, sensitive, or ethnic skin types (skin types > III).^29^ It is also effective for smokers and other individuals having been exposed to external pollutants.^30^

Several reported advantages have been discussed in the literature for micro‐needling.^31^ These include:

- Short healing times when compared to other modalities (typically 24–48 h).
- The technique is easy to master.
- Can be utilized on all skin types where lasers and deep peels cannot always.
- Convenient office procedure with minimal overhead cost.
- Well tolerated by patients.
- Minimal risk of post‐inflammatory hyperpigmentation or bruising since the needle depth penetrate the skin a maximum of 2.5 mm.

**Table:** 

**FIGURE 10:**
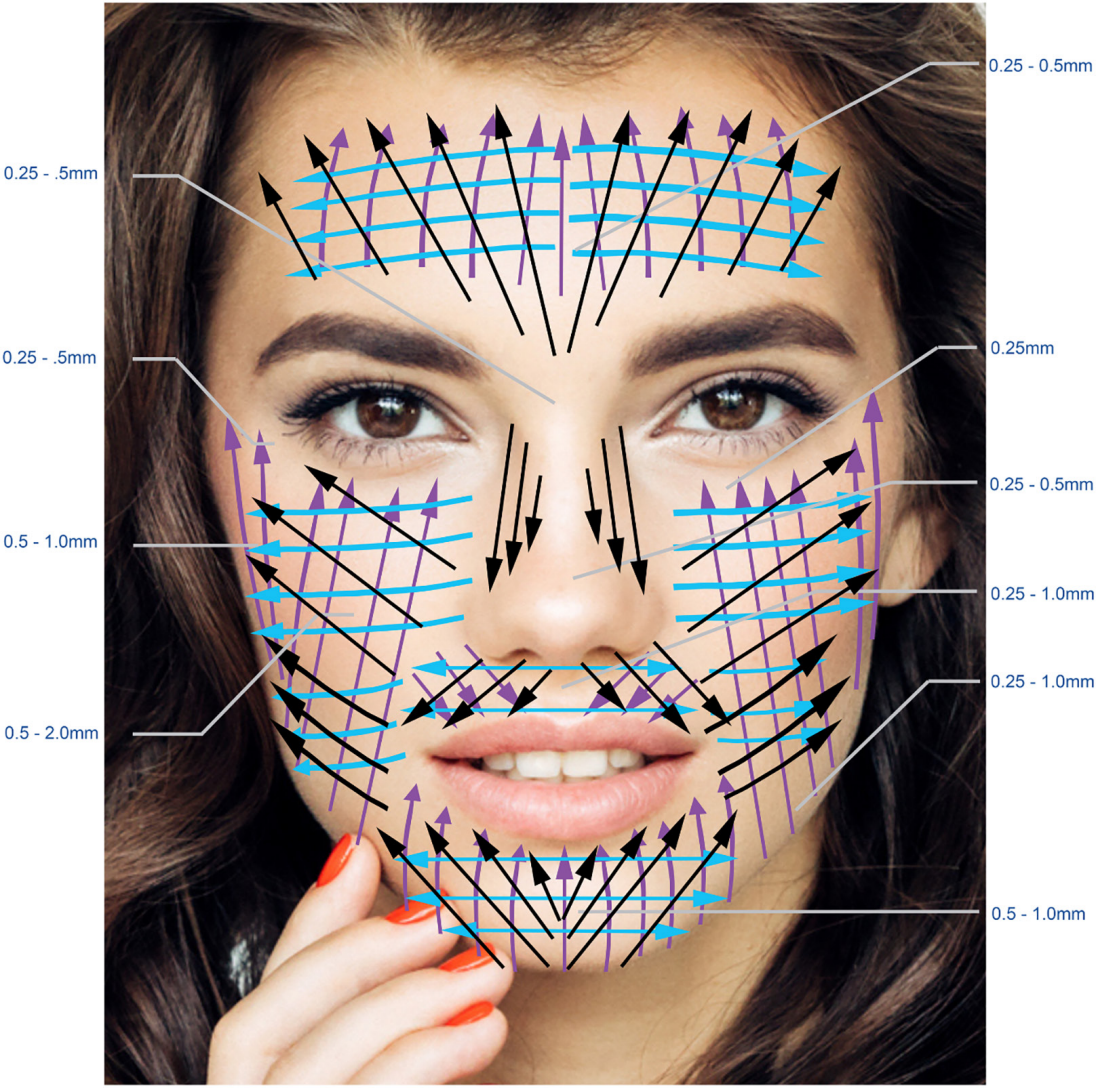
Microneedling Depth and Directional Chart, demonstrating a three directional approach to microneedling. Reprinted with permission from Davies/Miron.^8^

Tips Use compounded topical anesthesia (pharmacy based and not over the counter) for at least 30 min prior to procedure and ensure it is well removed before beginning. Ensure skin is well lubricated with APCs to avoid a dry tugging sensation. Use Directional and Depth chart for guidance (Figure 10). Do not microneedle over tattoos or permanent make up. Map out problem areas such as scarring or acne scars for special treatment with stamping techniques at great depth. Advise patient to avoid sunlight and heavily scented facial creams/products for 24 h post op.

**FIGURE 11:**
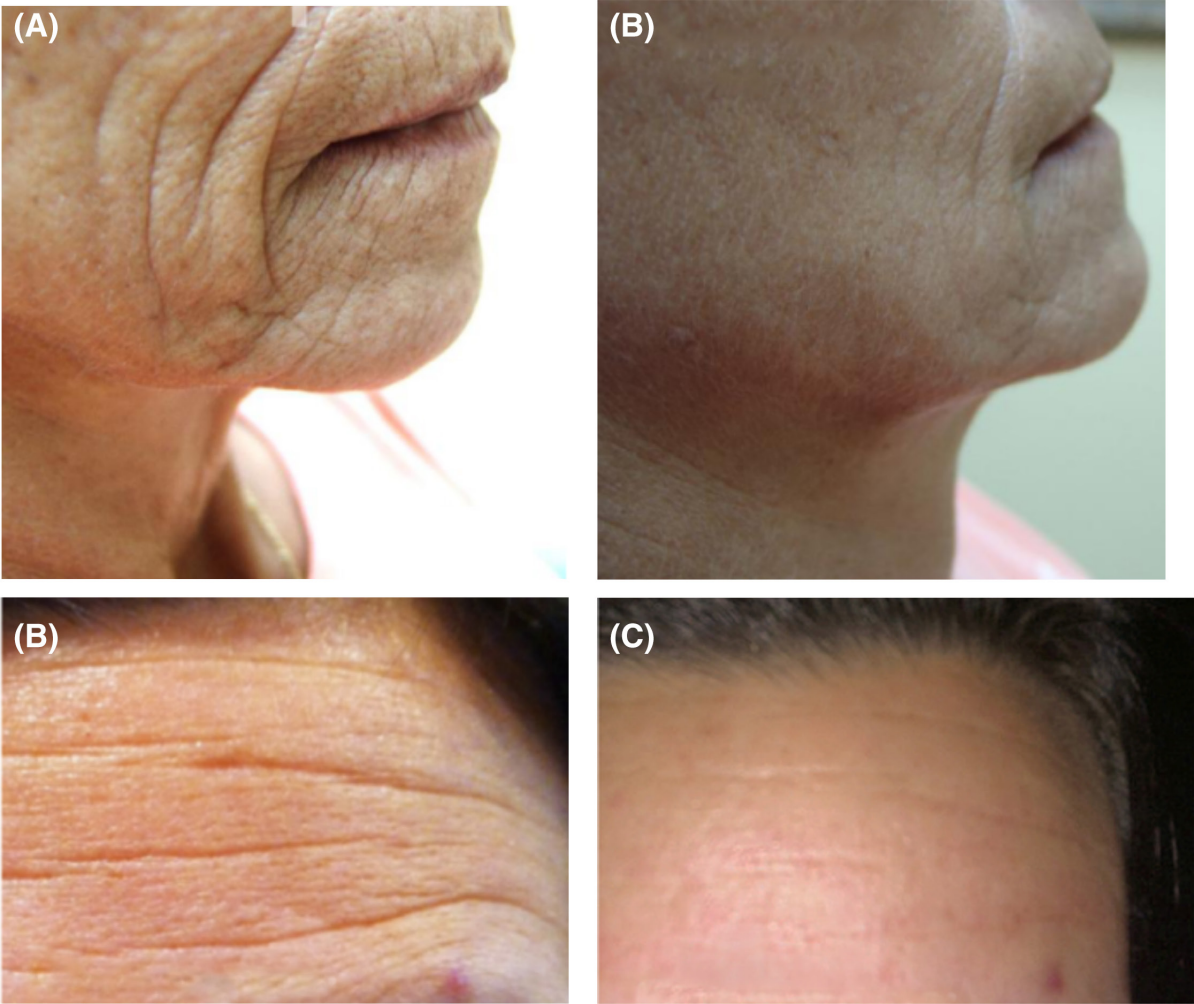
(A) Clinical photo demonstrating older female patient with pronounced deep facial wrinkles. (B) Results following four treatment procedures 1 month apart. Note the substantial reduction in depth of each wrinkle post‐op. (C) Male patient (cigarette smoker) with substantial forehead wrinkles. (D) Following four micro‐needling treatments, note the substantial improvement in facial harmony and reduction in deep forehead wrinkles. Reprinted with permission from Davies/Miron.^8^

Specific directions and depths are recommended for optimal results (Figure 10) and safety with minimal downtime.^32^ Before and after pictures are presented in Figure 11 with an accompanying short video highlighting the use of microneedling with PRF QR Code 1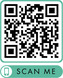.

### 5.2 Intradermal injection of APCs

Precise intradermal injections deliver APCs in a very safe manner to the desired area using very small gauge needles (typically 30 gauge 4 mm needles are recommended).^33^ Injections just below the dermis, allow for a high concentration of growth factors to be delivered to specific troubled areas such as crow's feet, glabella area, smokers lines, neck lines, etc. Intradermal papule injections do not place the product into the deep underlying vasculature which lies well below the dermis layer, in the hypodermis. These provide extremely safe injections. Formation of a papule with blanching while injecting confirms that the injection is in the intradermal layer.

**FIGURE 12:**
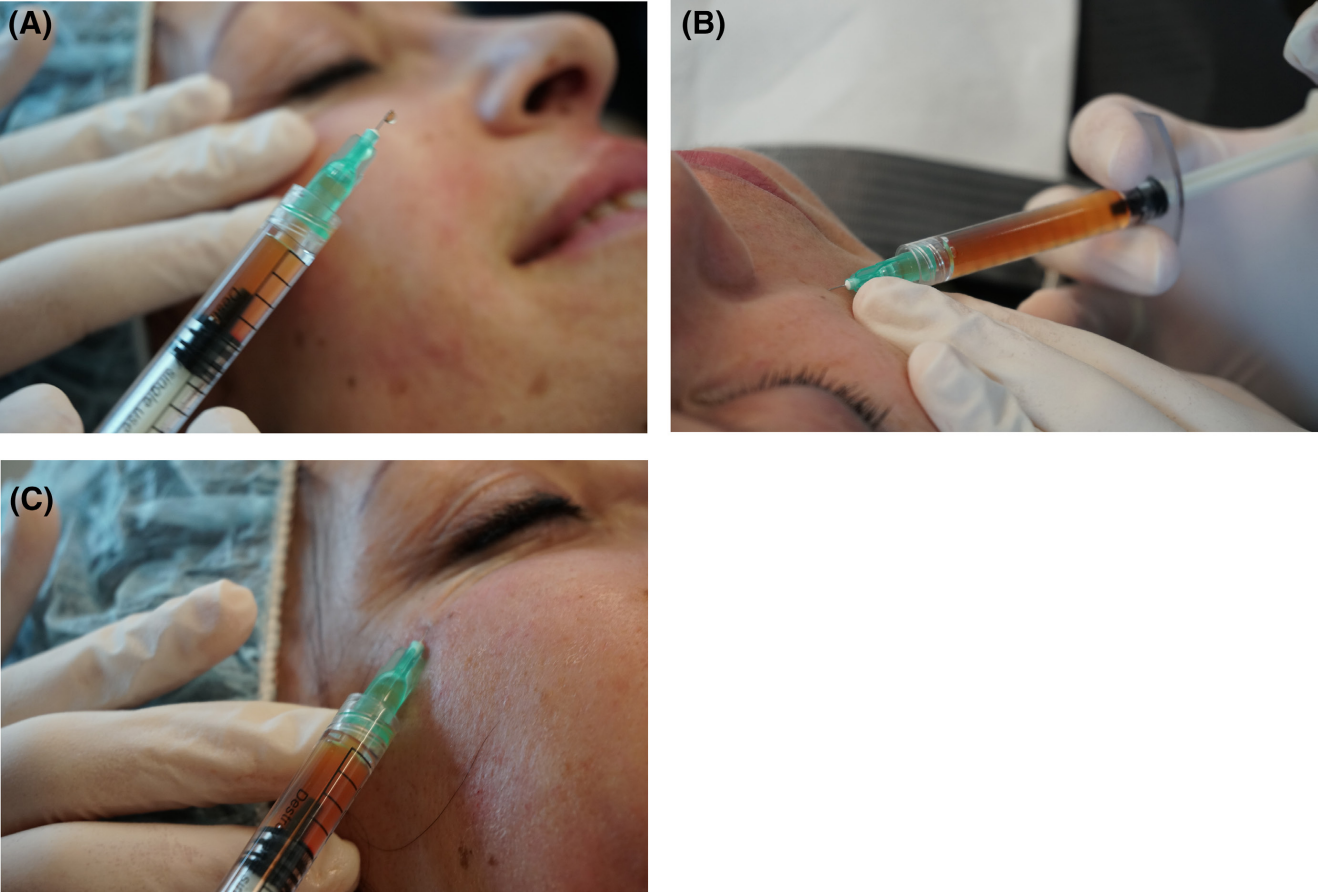
(A) Photograph of a 30 gauge 4 mm needle, (B, C) demonstrating precise intradermal injection technique. Reprinted with permission from Davies/Miron.^8^

The procedure involves injecting a small amount of APCs into the intra dermal layer, forming a papule. The injections are minimally invasive aimed at treating specific conditions such as fine lines, wrinkles, UV damage, acne scars and overall rejuvenation. Figure 12 demonstrates the use of intra‐dermal papule injections (QR Code 2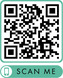). It highlights the ease of such a technique which delivers APCs in a very safe modality using a 30 gauge 4 mm needle. These injections allow for a higher delivery of growth factors to specific troubled areas (such as Crow's feet, glabellar lines and deep nasolabial folds).

**Table:** 

Tips: Use small gauge needles such as 30G, 4 mm length. Injections can be performed 5 mm from one another. Ensure the bevel of the needle is facing up for an optimal papule. Inject problem areas before microneedling when APC is in its most liquid form. Treatments should be at least one month apart, for three to four sessions. Thereafter, maintenance can be performed every 6 to 12 months.

If PRF is used, it’s important to note that the formulation will clot within 20‐40 minutes if left in the syringe. Additionally, exposure to oxygen, such as by opening the cap of the tube, will cause it to clot much more quickly. From here, PRF may be utilized as an injectable device either into facial tissues or into the scalp in a similar fashion as PRP. It may also be utilized as an autologous growth factor applied to the face prior to/after micro‐needling in a similar fashion to PRP in the vampire facelift technique.^34^, ^35^

## 6 APCs FOR ACNE SCARS

### 6.1 Background

APCs serve as adjunct therapy for atrophic acne scars, with the primary therapy including not only microneedling, but also fractional carbon dioxide laser, and subcision. Microneedling has been known to be an effective treatment option for acne scars; however, the addition of APCs has only recently been investigated. The upregulation of growth factors associated with APCs is believed to augment the effects of microneedling to promote esthetically superior tissue remodeling. Their synergistic effects offer a unique treatment approach.

### 6.2 Outcome

Eleven clinical studies investigated the use of APCs for managing acne scarring. Of these, eight studies used PRP, which was administered via microneedling, two studies used PRF, and one study combined PRF with microneedling. Additionally, one study used PRP as an adjunct to ablative CO_2_ laser treatment 8^36^, ^37^, ^38^, ^39^, ^40^, ^41^, ^42^, ^43^ of the studies used PRP, which was administered by microneedling, and 2 studies utilized PRF,^44^, ^45^ and one both.^26^ One study utilized PRP as an adjunct to ablative CO_2_ laser.^42^

The primary outcome measure were various improvement scores assessed by using Goodman's Qualitative Scale (GQS) in pre‐ and post‐treatment evaluation (Table 2). The findings from these studies indicate that the combined treatment with microneedling and APC is associated with higher odds of achieving notable/complete clinical improvement compared to microneedling therapy without PRP or with other topicals such as vitamin C. The efficacy of combined treatment was evidenced by the higher rate of significant improvement and better improvements in the Goodman and Baron Acne Scar Scale including a higher satisfaction rate from patients. Only one study, conducted by Gupta et al.,^38^ demonstrated no added advantage of topically applying PRP over microneedling for acne scars (Table 2). It is worth noting that in this study, PRP was applied after microneedling, which may explain the difference in outcomes as in all other studies, PRP was applied prior to with the needling effect pushing the APCs into the skin.

**TABLE 2:** APC for acne scars.

Author	Study type	Subjects Gender Age Condition % Smokers	Centrifuge rpm/min *g* force Anticoagulant	Treatment protocol and follow‐up	*Grading System* Outcome
PRP microneedling for acne scars					
Chawla et al.^36^	*NRCT* *spl‐f*	27 patients ♀ = 27/♂ = 0 Age: 18–34 years Acne scar gr2–4	PRP double spin 1500 rpm 10 min 3700 rpm 10 min AC used	4 micro needling sessions 30 days interval RHS’ N S LHS Vitamin C	FU 4 months *GQS* PRP poor response rate: 22.2% Vitamin C poor response rate: 37% (*p* = 0.021) Patient satisfaction greater with PRP (*p* = 0.01) Final conclusion: PRP combined with microneedling is a better treatment option than microneedling with vitamin C in treating atrophic acne scars
Ibrahim et al.^37^	*RCT*	90 pts ♀ = 46/♂ = 44 Mean Age: 16–40 years Acne scars	Double spin 1419 *g* 7 min 2522 *g* 5 min AC sodium citrate	Rx: 6 sessions or patient satisfaction Group 1: MN 1 session every 4 weeks Group 2: Intradermal PRP: 1 session every 2 weeks Group 3: Combination MN plus intradermal PRP, alternating every 2 weeks MN Device: 0.25–2.5 mm length	Colored photographs (assessed by 2 dermatologists) *GQS* Patient satisfaction on a quartile scale Group 1: mean improvement (39.71 ± 13.06) Group 2: mean improvement (48.82 ± 23.74) Group 3: mean improvement (70.43 ± 13.32) SS difference between the studied groups with higher response in group 3 followed by group 2 and lastly group1 (*p*‐value < 0.0.001) Final conclusion: Combination between skin needling and PRP is more effective in all types of acne scars
Gupta et al.^38^	*NRCT* *Spl‐f*	36 patients ♀ = 19/♂ = 17 Age: 21–30 years Acne scars	Double spin 1400 rpm 10 min 3500 rpm 10 min	4 sessions: 1 month apart RHS: MN with PRP applied after LHS: MN alone 2 mm	FUs: baseline and second, fourth, and sixth visits. was evaluated by both physicians and patients. RHS vs. LHS Visual Analog score (VAS): evaluated by patient and physician showed maximum improvement at second and third visits, respectively The mean total scars declined with insignificant differences (*p* = 0.094) Final conclusion: This study showed *no* added advantage of topical application of PRP over microneedling in acne scars
Sharma et al.^39^	*NRCT* *Spl‐f*	40 patients ♀ = 27/♂ = 13 Age: 17–31 years Acne scars GR 1–4	Single spin 3600 rpm 15 min AC: ACD	Grp A RHS MNPRP Grp B LHS – MN saline Depth 1.5 mm 4 treatment 1 month apart	FU end of 4 months *GBS* Group A: 3.20 ± 0.40 at baseline, to 2.13 ± 0.56 Group B: 3.20 ± 0.40 to 2.36 ± 0.56 Final conclusion: Combination approach using MN and PRP is a better option than using MN alone in atrophic acne scars for clinical improvement, although not SS
Asif et al.^40^	*NRCT* *Spl‐f*	50 pts Age: 17–32 years Gr2‐3 acne scars	Double spin 294 *g* 5 min 691 *g* 17 min	Grp A RHS MN first then PRP intradermal Grp B LHS – MN first then distilled water intradermal 3 treatments 1 month apart 1.5 mm depth	FU 12 weeks (GQS) Excellent in 60% Excellent in 40% Final conclusion: PRP has efficacy in the management of atrophic acne scars. It can be combined with microneedling to enhance the final clinical outcomes
Nandini et al.^43^	*NRCT* *Spl‐f*	30 patients ♀ = 12/♂ = 18 Age: 20–40 years Acne scars gr2–4	Single spin 3600 rpm 15 min AC: ACD	Grp A RHS MN With PRP Grp B LHS – MN alone Four treatments once monthly	FU 12 months *GQS*: Gr A 13 (43%) patients – excellent response Group B 6 (20%) patients – excellent response Patient's satisfaction Gr A 11 (36%) patients had more than 75% Group B 1 (3%) patient had more than 75% The study showed a decrease in scar severity grade in all the patients Final conclusion: A combination of PRP + MN was found to be more effective than a single method used for the treatment of acne scars
Amer et al.^41^	*NRCT* *Spl‐f*	41 patients ♀ = 28/♂ = 13 Age: 20–40 years Gr2‐4 acne scars	1600 rpm 10 min 4000 rpm 15 min, *t*	Grp A RHS MN first then PRP Grp B LHS – MN first then HA 4 treatments 1 month apart	FU > 4 weeks *GQS*& quartile grading scale Right showed 85.4% improvement, (statistically significant) Left halves and 82.9% improvement, (statistically significant) The difference of the improvement between the two modalities is statistically insignificant *p* > 0.05 Final conclusion: MN has efficacy in the management of atrophic acne scars. It can be combined with either PRP or noncross‐linked hyaluronic acid to enhance the final clinical outcomes in comparison with microneedling alone
Gawdat et al.^42^	RCT *Spl‐f*	30 patients ♀ = 18/♂ = 12 Age: 20–40 years Acne gr 2–4	Double spin 150 *g* 15 min 400 *g* 10 min AC: ACD Activator: CaCl	3 sessions 1 month apart. CO_2_ laser + Intradermal‐PRP RHS intradermal NS LHS Intradermal‐PRP RHS topical—PRP LHS	FU 6 months Intradermal or topical PRP showed SS in skin smoothness > saline‐treated area (*p* = 0.03)‐ NS between intradermal PRP and topical PRP (*p* = 10) In in areas treated with PRP, leading SS shorter downtime (*p* = 0.02) Final conclusion: PRP shorter downtime than CO_2_ laser alone and better tolerability than the laser combined with ID PRP.
PRF microneedling for acne scars					
Krishnegowda et al.^44^	*Spl‐f*	40 patients ♀ = 20/♂ = 20 Age: 18–50 years Acne scars	REMI R4C centrifuge 700 rpm 3 min 60 *g* RCF NO AC	*Spl‐f* Grp A RHS first PRF the MN Grp B LHS – first then saline then MN 4 treatments 1 month apart	FU 2 months Goodman and Baron Scale (GBS) Baseline mean GB grade: 3.45. At 24 weeks, mean GB grade was significantly reduced on the study side (1.47, SD 0.56) than control side (3.33, SD 0.53). Final conclusion: Mean patient satisfaction score was significantly higher on the right side (5.95) compared with the left side (5.35). Rolling scars responded the best followed by boxcar and the ice pick scars MN and PRF act synergistically to improve acne scars
APCs for intradermal injection in acne					
Shashank et al.^45^	*CS*		800 rpm 4 min No AC	PRF: intradermal injection Acne scars, Rejuvenation, Hair loss in	Final conclusion: PRF produced positive clinical outcomes in: acne scars, rejuvenation, hair loss.
PRP vs. PRF for skin acne scarring using both microneedling and/or intradermal injection					
Diab et al.^26^	*RCT* *Spl‐f*	30 patients With acne scars Group 1 PRP: ♀ = 13/♂ = 2 Age: 18–33 years Group 2 PRF: ♀ = 11/♂ = 4 Age: 22–38 years	Group I PRP double spin 900 rpm 5 min 2000 rpm 15 min AC EDTA Activated: 10% CaCl_2_ Group II PRF 700 rpm 3 min	Group I LHS intradermal PRP RHS: MN with PRP Group II LHS intradermal PRF RHS: MN with PRF 4 sessions with 3 weeks interval	FU 1 month after last treatment GBGS The acne scars significantly improved in both sides of face in both groups (3‐fold). According to; the therapeutic response was significantly higher in PRF (5‐fold) group than PRP (3‐fold) either alone or combined with needling Final conclusion: PRF is highly effective, safe, and simple procedure that can be used instead of PRP with better outcomes in the treatment of acne scars. The combination with needling increases efficacy of PRF and PRP. Fluid

*Note*: Acne Grading: Grade 1: Also known as “comedones,” and is categorized into two types, open and closed. Grade 2: Inflammatory lesions present as a small papule with erythema. Grade 3: Pustules. Grade 4: Many pustules coalesce to form nodules and cysts called nodulocystic acne.

Abbreviations: Study type: CCT, controlled clinical trial; CS, case series; non‐R, nonrandomized; RCT, randomized controlled trial; spl‐F, split‐FACE; Centrifuge data: *g*, *g*‐force; rpm, revolutions/rotations per minute; APC preparation: AC, Anticoagulant; Administration: HA, hyaluronic acid; ID, intradermal; MN, microneedling; Outcomes: GBS, Goodman Baron scale; GQS, Goodman's qualitative scale; NR, not reported; NS, Rx Treatment.

### 6.3 Conclusion

**FIGURE 13:**
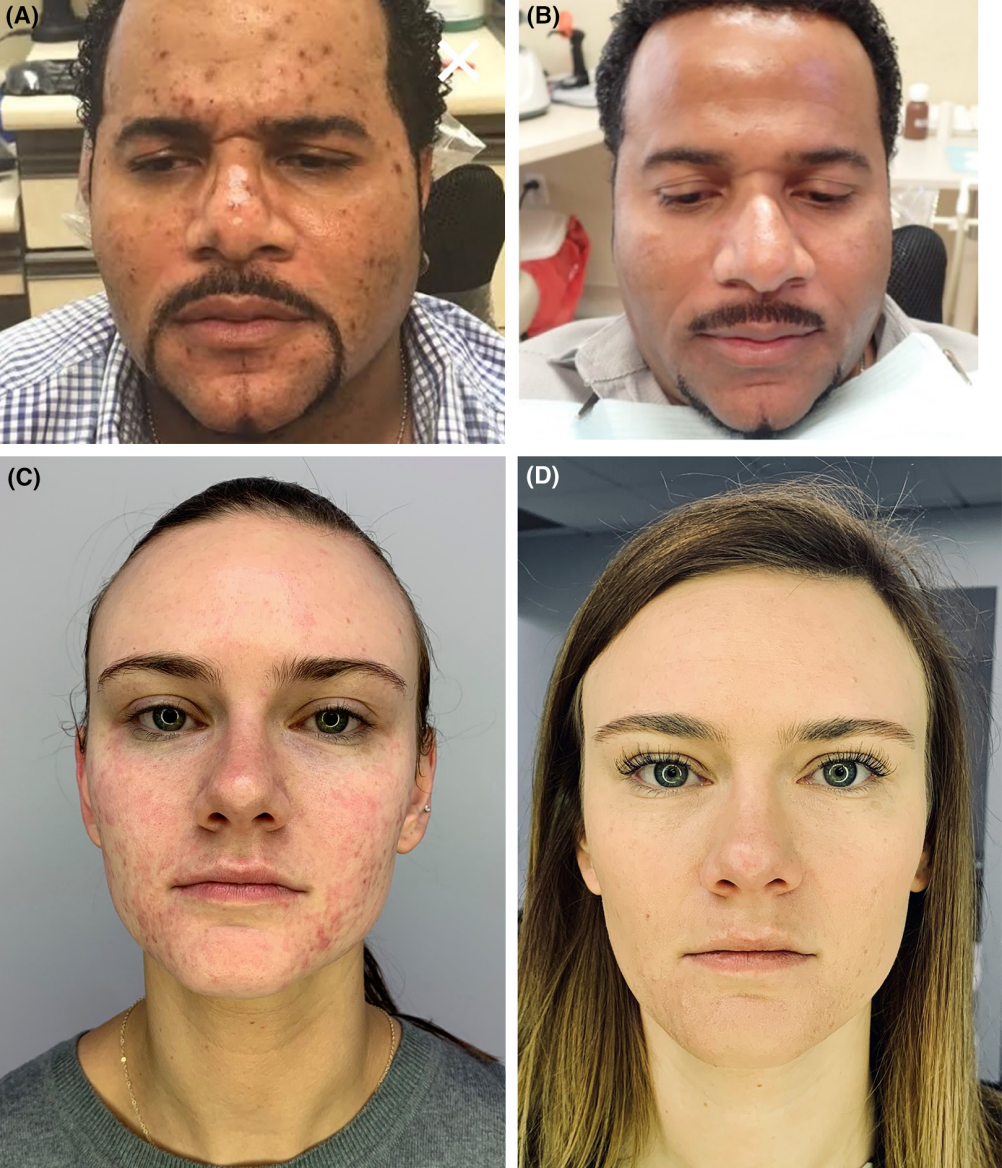
(A, B) Before and After of male patient with acne throughout his mid 30s treated with microneedling + PRF. (C, D) Young teenage female treated with microneedling + PRF as opposed to utilizing common prescription medication such as Accutane.

Overall these findings support the use of APCs as an adjuvant therapy for patients with acne scars undergoing microneedling treatment, without a significant increase in the risk of adverse events. Microneedling combined with APCs is more effective than the use of microneedling without APCs. The follow‐up period ranged from 1 to 3 months. The timing of the results should be assessed and analyzed in subsequent research to determine the optimal follow‐up interval. These procedures are routinely performed with marked clinical outcomes (Figure 13).

One study compared the therapeutic responses of PRP and PRF Diab et al.,^26^ both alone and in combination with microneedling. The improvement was significantly greater in the PRF group compared to the PRP group, whether used alone or combined with microneedling. Treatment of Left Side of Face: Group 1 received Intradermal injection of PRP, Group 2 received Intradermal injection of PRF. Treatment of Right Side of Face: Group 1 received microneedling of PRP, Group 2 received microneedling of PRF.^26^ The study reported outcomes using the following assessment:

**Table:** 

Goodman and Baron's global scarring grading system (GSGS): by comparing its values before the start of treatment and 4 weeks after the last session. Quartile grading scale: the improvement was classified into: excellent if improvement >75%; very good improvement 50–74%; good 25–49% and poor improvement <25%. Patient's satisfaction: the patients assessed their degree of improvement as poor, good, very good and excellent. All patients were also asked to rate their pain on a scale of 0 to 10. 0 means no pain and 10 means the worst pain.

The findings demonstrated that for both PRP and PRF, microneedling led to a 3‐fold improvement in “excellent” results of microneedling with PRP (20% of participants) versus intradermal injections with PRP (6.7% of participants) and roughly a 2‐fold increase when comparing microneedling with PRF (53.3% of participants) versus intradermal injections with PRF (33.3% of participants). Comparative results investigating PRF versus PRP led to a 5‐fold increase in reported “excellent” results when PRF intradermal injections (33.3% of participants) were performed versus PRP intradermal injections (6.7% of participants).^26^

**FIGURE 14:**
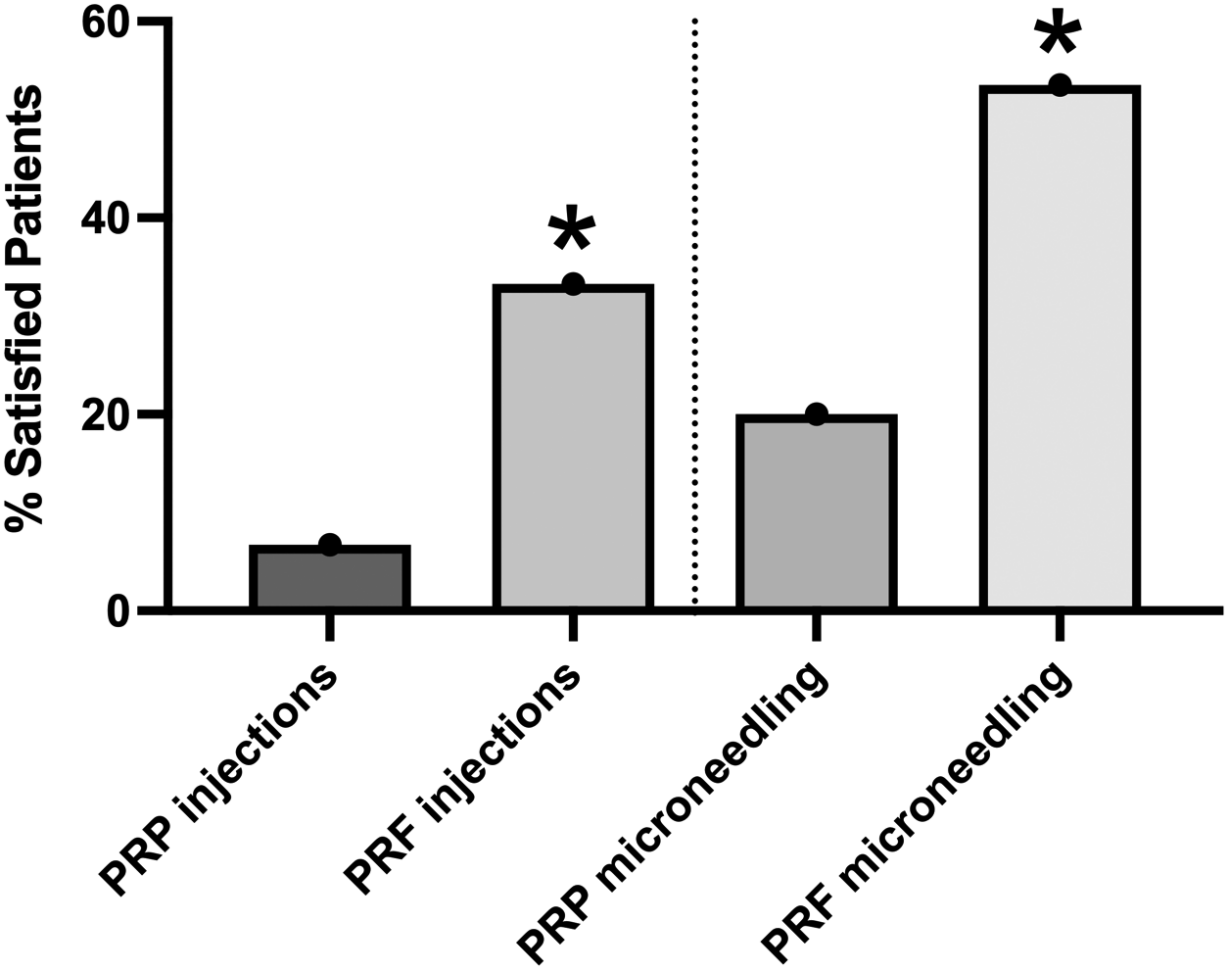
Patients with scars were assigned to one of the following four groups. (1) PRP injections, (2) PRF injections, (3) PRP microneedling, and (4) PRF microneedling. Overall a fold 5 higher “excellent” reported outcomes was found in the PRF injection group when compared to the PRP injection group. Additionally, an over 3 fold “Excellent” reported score was found in the PRF microneedling group when compared to the PRP microneedling group. In either case, microneedling was better for APC scar treatment when compared to injections. Results derived from Diab et al.^26^ * represents p < 0.05.

Comparative results investigating microneedling also demonstrated over a 2‐fold increase of microneedling with PRP (20% of participants) versus microneedling with PRF (53.3% of participants) (Figure 14).^26^ The findings concluded that microneedling with either PRP/PRF led to better results than intradermal injections with PRP/PRF. In either comparison, PRF always led to significantly better results. The severity of the scar reduction as assessed by GSGS showed more improvement in PRF versus the PRP group.^26^

### 6.4 Clinical guidelines

The protocol using APCs for acne requires three treatments, 1 month apart. Thereafter, one treatment is recommended every 6 months for maintenance. Patients are advised to follow a homecare routine to prevent further break outs if active acne is still present. This includes *cleaning skin gently with a mild, nondrying face wash and use of non comedogenic products, and or prescribed anti acne oral or topical formulations*.

## 7 APCs FOR SKIN REJUVENATION

### 7.1 Background

APCs are widely used in the field of facial esthetics for skin rejuvenation and treatment of photoaging. One of the proposed underlying therapeutic mechanism of APCs in skin rejuvenation involves remodeling of the extracellular matrix via increased expression of matrix metalloproteinases, proliferation of fibroblasts, and stimulation of collagen synthesis.^46^ Both microneedling and intradermal injections of APCs are emerging as promising treatment modalities for skin rejuvenation, and they can potentially be used as an anti‐aging modality and for improving the skin quality, skin tone, and skin texture.^47^ Various modalities have been used to measure outcomes as presented below:

**Table:** 

Outcome measures: facial rejuvenation Wrinkle severity rate scale (WSRS) grade 0 = no wrinkles, grade I = just perceptible wrinkle, grade II = shallow wrinkles, grade III = moderately deep wrinkles, grade IV = deep wrinkle with well‐defined edges, grade V = very deep wrinkle with redundant fold. Wrinkle assesment scale (WAS)‐improvement of one point or more is a responder. Skin parameter measurements: spots, wrinkles, texture, pores, ultraviolet (UV) spots, brown spots, red area, and porphyrins. Global Aesthetic Improvement Scale (GAIS): Blinded dermatologists were asked to assess improvement using a 5 point scale No improvement, mild improvement,moderate improvement, marked improvement , excellent improvement. Patient's satisfaction: graded the patient was very satisfied, satisfied, slightly satisfied or not satisfied. Antera camera system (3D, Miravex Limited, Ireland) – A skin analysis tool VISIA‐CR System (Canfield Imaging Systems, Fairfield, New Jersey, USA) obtain high‐resolution facial photographs. Ultrasound (16 MHz high‐frequency)‐Dermal Thickness assessment. FACE‐Q IS a patient‐reported outcome tool.

### 7.2 Outcome

El‐Domyati investigated microneedling alone vs microneedling plus PRP and microneedling plus trichloroacetic acid (TCA).^48^ Microneedling plus PRP demonstrated better skin structural improvements than microneedling alone or with TCA. More commonly, studies investigating skin rejuvenation have evaluated APCs as intradermal injections as further highlighted in the next section.

18 studies were reviewed in total, 10 investigated PRP^48^, ^49^, ^50^, ^51^, ^52^, ^53^, ^54^, ^55^, ^56^, ^57^ and 8 investigating PRF^45^, ^47^, ^58^, ^59^, ^60^, ^61^, ^62^, ^63^ as either a stand‐alone or adjunct therapy to other cosmetic treatments (Table 3). Despite the wide spread use of PRP as a regenerative agent, efficacy remains inconclusive. Although mostly promising, PRP studies were heterogenous and difficult to compare due to a lack of standard dosing, preparation techniques, and subjective outcomes measurements.

**TABLE 3:** APCs for skin rejuvenation using microneedling or intradermal injections.

Author	Study type	Subjects Gender Age Condition % Smokers	Centrifuge rpm/min *g* force Anticoagulant	Treatment protocol and follow‐up	Main outcome *Grading System*
PRP for skin rejuvenation using microneedling					
El‐Domyati et al.^48^	*CCT*	24 patients ♀ = 24 Age: 18–33 years Photoaging	Double spin: 252 *g* 10 min 1792 *g* 5 min Activator: CaCl_2_	Administered by microneedling. Group 1 MN alone Group 2 MN + PRP or Group 3: MN + TCA 15% peeling. One session every 2 weeks for six sessions	FU after 3 months Photographs and punch biopsies Combined treatment of MN + PRP or MN + TCA showed significant improvement when compared with MN alone SS increase in epidermal thickness was apparent in studied groups, especially after MN + TCA. Final conclusion: Improvement of dermal structures was better demonstrated after combined treatment of MN + PRP than MN + TCA 15%.
PRP for skin rejuvenation using intradermal injection					
Du et al.^49^	*CCT* *Spl‐f*	30 patients ♀ = 30/♂ = 0 Age: 30–50 years Facial aging	PRP double spin 110 *g*, 15 min 1400 *g*, 8 min AC trisodium citrate	Three autologous PRP injections with 15 day intervals LHS PRP RHS normal saline	*VISIA® Complexion Analysis System CT data collected week 0, week 2, week 4, week 6* PRP injections improved skin quality: wrinkles, texture, pores Final conclusion: PRP injections are effective in improving skin conditions and protecting skin from photoaging
Abuaf et al.^50^	*NCCT*	20 patients ♀ = 20/♂ = 0 Healthy females	Kit used: Regen Lab Single spin 3000 rpm 5 min AC: sodium citrate Activator CaCl_2_	Intradermal PRP injection Control: intradermal saline 2 mL into dermis of face via point by point	Mean optical density (MOD), Biopsies PRP side: higher mean optical density (MOD) of collagen (1019) compared to pre‐treatment (539) and saline injection (787) (*p* < 0.001 for PRP side). Patients receiving intradermal PRP treatment had an improvement of 89.05%, (control: 46.01%) Ratio from PRP to control was 1.93:1 (*p* < 0.001 for both). Final conclusion: PRP is a safe effective treatment for facial skin rejuvenation
Alam et al.^51^	*RCT* *Spl‐f*	27 patients ♀ = 17/♂ = 10 Age: 18–70years Photoaging Glogau class 2,3	SmartPrep 2 System; Harvest Technologies AC; ACD	3 mL intradermal injections of PRP to one cheek and sterile NS to the contralateral cheek. NLF and cheek Single session	FU 24 weeks Photoaging scores No significant difference between PRP and NS ne in fine lines, mottled pigmentation, skin roughness, or skin sallowness Self‐assessment scores were higher for the PRP‐treated side compared with the normal saline side.in texture (2.0 vs. 1.21 *p* = 0.02) and wrinkles (1.74 vs. 1.21, *p* = 0.03) 6 months after injections Final conclusion: Masked participants noted that both fine and coarse texture improved significantly more with a single treatment of PRP than with normal saline. Both participants and raters found PRP to be nominally but not significantly superior to normal saline
Everts et al.^52^	*RCT*	11 patients ♀ = 11/♂ = 0 Age: 45–65 years	Emcyte Pure PRP system AC: sodium citrate	Intradermal PRP Three session injections 1 month apart	FU 6 months (56 days) Biometric parameters and Self Assessment Score SS decrease in brown spot counts and area (*p* < 0.05) SS Wrinkle count and volume reduced (*p* < 0.05 for total wrinkle appearance) SS Skin firmness improved SS Skin redness improved after 169 days post‐therapy for both the nasolabial and malar areas SS increase in SLEB density (*p* < 0.05 for both parameters) Average satisfaction score of >90%. Final conclusion: PRP injections resulted in SS skin rejuvenation
Gawdat et al.^53^	*RCT*	20 patients ♀ = 20 Age: 35–60 years Glogau 2, 3 (neck)	Double spin PRP 151 *g* 15 min 605 *g* 10 min AC: ACD	Group A: PRP + Fractional RF microneedling Group B: Fractional RF microneedling only Three sessions 1 month apart	*GAIS* Mean dermal thickness after treatment was higher in group A compared with B (statistically insignificant) GAIS: More favorable results were reported in group A Final conclusion: Fractional MN RF offers a safe and effective modality for mild to moderate neck laxity when used alone or in combination with PRP
Basyoni et al.^54^	*RCT* *Spl‐f*	20 patients ♀ = 20/♂ = 0 Age: 36–57 years	Double spin method 3000 rpm 7 min/4000 5 min AC: sodium citrate Activator: CaCl	Group A MN IACM Group B MNPRO Six sessions 2 weeks apart	FU 2 weeks after last Rx WSRS, Antera camera SS difference between both sides Higher response in side (A) % improvement (*p* value > 0.001) and GAIS (*p* value = 0.002). Skin Biopsy: Both sides After treatment, neocollagenosis, increase in collagen bundle deposition and thickness were noticed Final conclusion: MN IACM is more effective than MN with PRP for facial rejuvenation
Navarro et al.^55^	*CS*	9 patients ♀ = 9 Age: 36–65 years Glogau’s 2 3	System V: 580 *g* 8 min Part of the plasma: thermally gelated (76°C 12 min) AC: trisodium citrate Activator: calcium chloride	Non ablative laser plus PRP solution (topical) Home use bd for 8 weeks	Assessed 8 weeks after beginning Rx VISIA‐CR System Combined therapy with home use improved cutaneous spots, wrinkles, and texture after 8 weeks, whereas significant pore reduction was observable at 1 week (*p* ≤ 0.05). Final conclusion: Overall wrinkle amelioration, periorbital hyperpigmentation decrease, softened skin, and tone recovery was observed. Patients referred to be very satisfied and felt that their cutaneous condition was much better
Hersant et al.^56^	*RCT*	93 patients ♀ = 87/♂ = 6 Age: > 40–64 years Smokers 0%	Regen lab PRP with AC Combined with 40 mg of noncrosslinked natural HA	Single session Intradermal injections Group 1: Mixture 2 mL:2 mL (PRP:HA) (Cellular Matrix) Group 2: PRP only Group 3: HA only	*F*U month 0, 3 and month 6 FACE Q and Biophysical measurements Treatment with Cellular Matrix: SS improvement overall facial appearance compared with treatment PRP or HA alone (*p* < 0.0001). cellular matrix group: showed a 20%, 24%, and 17% increase in FACE‐Q score at 1, 3, and 6 months HA group, the improvement in FACE‐Q score was 12%, 11%, and 6% at 1, 3, and 6 months PRP group the improvement was. 9%, 11%, and 8% at 1, 3, and 6 months SS improved skin elasticity for the Cellular Matrix group compared with the groups receiving a‐PRP or HA alone No serious adverse events were reported Final conclusion: Combining RP and HA seems to be a promising treatment for facial rejuvenation with a highly significant improvement in facial appearance and skin elasticity compared with PRP or HA alone
Willemsen et al.^57^	*RCT*	25 patients ♀ = 25/♂ = 0 Age: 30–45 years	Not mentioned	Combination: Group 1: Lipofilling plus saline Group 2: Lipofilling plus PRP	FU 1 year Outcome: changes in skin elasticity, volumetric changes of the nasolabial fold, recovery time Final conclusion: Faster recovery was observed in the PRP group but no improved outcome for facial lipofilling when looking at skin elasticity improvement, graft volume maintenance in the nasolabial fold
PRF for skin rejuvenation using intradermal injection					
Nacopoulos et al.^58^	*NRCT*	32 patients ♀ = 25/♂ = 0 Age: 25–65 years Lower face aging (9 excluded)	Centrifuge: process for PRF 700 rpm 3 min 1300 rpm 5 min	Four sessions PRF with 2–3 weeks intervals (Cleopatra technique)	Photographical study Statistically significant percentage of true answers by the reviewers was noted upon completion of the treatment (*U* = 110.5, *p* < 0.001), Clinically significant effect of PRF in lower face treatment. Only few minor, self‐limited adverse events were recorded. Final conclusion: PRF is a well tolerated, effective method for lower face rejuvenation
Liang et al.^47^	*RCT*	231 patients 103—test 128—control ♀ = 91/♂ = 12 Age: 24–55 years	PRF 2700 12 min Nanofat by Coleman Collection method	Nanofat plus PRF (103) intradermal injection HA injection (control) (128) (Forehead cheeks chin)	FU 12 and 24 months Photographs and VISIA skin analysis Facial texture was improved to a greater extent after PRF and nanofat compared with HA PRF and nanofat had a higher satisfaction rate (significant) Both nanofat‐PRF and HA injection improve facial skin status without serious complications Final conclusion: Nanofat‐PRF injections are a safe, highly effective, and long‐lasting method for skin rejuvenation. Both improve facial skin status but Nanofat‐PRF better result than HA
Wei et al.^59^	*RCT*	62 patients ♀ = 50/♂ = 12 Age: 23–77 years Facial soft tissue depression and aging	PRF 2700 12 min	Nanofat and PRF injections (62) Autologus fat (77)—traditional fat transplant	Facial soft tissue depression symptoms and skin texture were improved to a greater extent after nanofat transplants than after traditional transplants, and the nanofat/PRF group had an overall satisfaction rate above 90% VISIA and SOFT5.5: skin texture, elasticity, pore size, and moisture improved, and trends towards improvement were also observed for wrinkles and splashes. *p* < 0.01 vs. before Final conclusion: PRF and autologous structural fat granules may therefore be a safe, highly‐effective, and long‐lasting method for remodeling facial contours and rejuvenating the skin
Sclafani et al.^60^	*CCT*	15 patients ♀ = 15/♂ = 0 Age:23–72 years NLF	Selphyl System 1100 *g* 6 min Activator: CaCl_2_	PRFM injected into the dermis and immediate subdermis below the NLFs‐single session	WAS FU 12 weeks after treatment. RESULTS: All patients were treated to maximal (no over‐) correction, with a mean reduction in WAS score rose to 1.13 ± 0.72 respectively (*p* < 0.001) No patient noted any fibrosis, irregularity, hardness, restricted movement, or lumpiness Final conclusion: PRFM provides long term dimunition of deep NLF
Hu et al.^61^	*RCT* *spl‐f*	30 patients ♀ = 30/♂ = 0 Age: 26–68 years Condition: mid cheek, NLF	Factor Medical LLC 6 min spin NO AC used	27‐gauge microcannula 4 mL of PRFM intradermally to cheek and NLF vs. NS	FU 6 weeks after last treatment VISIA Median change in total VISIA score (interquartile range) was −1.77 (2.36) in the PRFM group and −0.73 (2.09) in the saline group (*p* = 0.003). FU 12 weeks Texture showed statistically significant change (*p* = 0.004). change in median score was −1.31 (3.26) in the PRFM cohort and −0.76 (2.21) in the saline cohort (*p* = 0.34) Final conclusion: The PRF can objectively improve skin quality. The results persist for at least 6 weeks
Fernanda et al.^62^	*NRCT*	26 patients ♀ = 26/♂ = 0 Age: 35–55 years	2700 rpm/RCF 3 min	Three intradermal i‐PRF sessions spaced 21 days apart	FU 21 days after the final session SS dermal thickness increase post PRF: glabella (*p* < 0.00269), frontal D (*p* < 0.00018), frontal E (*p* < 0.00014), cheek D (*p* < 0.00709), and cheek E (*p* < 0.0008) Self‐Perception Index yielded a *p*‐value < 0.0001, SS self‐perception change post‐treatment Final conclusion: Intradermal PRF application markedly increased dermal thickness, endorsing its potential for dermal restructuring. Furthermore, this approach presents an accessible, cost‐effective, and unbiased alternative for facial rejuvenation
Hassan et al.^63^	*CS*	11 patients ♀ = 11/♂ = 0 Age: 33–60 years	PROCESS i‐PRF 700 rpm 3 min 60 *g* No AC	Monthly intradermal i‐PRF injections in 3 areas of face: Malar areas nasolabial fold upper lip skin	FU 3 months *GAIS scores* SS improvement in skin surface spots (*p* = 0.01) and pores (*p* = 0.03) skin texture, wrinkles, ultraviolet spots, and porphyrins, showed a numerical improvement FACE‐Q: SS improvement from baseline; satisfaction with skin (*p* = 0.002), satisfaction with facial appearance (*p* = 0.025), satisfaction with cheeks (*p* = 0.001), satisfaction with lower face and jawline (*p* = 0.002), and satisfaction with lips (*p* = 04). No major adverse effects were reported Final conclusion: PRF created a remarkable skin rejuvenation that was evident at the 3‐month follow‐up visit
Shashank et al.^45^	*CS*	4 patients ♀ = 3/♂ = 1	— 800 rpm 4 min. No AC	PRF: Acne scars, Rejuvenation, Hair loss in	PRF produced positive clinical outcomes in acne scars, rejuvenation and hair loss. Final conclusion: Preparation of injectable PRF is simple and requires minimal instrumentation and materials, making it a cost‐effective option for facial esthetics
PRP vs. PRF for skin rejuvenation using intradermal injection					
Atsu et al.^64^	NRCT	55 patients ♀ = 54/♂ = 1 Age: 23–58 years	Tlab kits PRF: no AC PRP: sodium citrate Both 2000 rpm 2 min	3 injections 1 month apart Group A: PRP (23) Group B: PRF (32)	FU 3 months after treatment SS: canthal smoothness and wrinkle (lower) scores for PRF group, while For canthal smoothness, at only 3 months the difference reached SS Final conclusion: SS superiority (marginal) of PRF over PRP, only for the treatments of the canthal region and only at 3 months

*Note*: Assessment, Glogau Wrinkle Scale I—no wrinkles, II—wrinkles in motion, III—wrinkles at rest, IV—only wrinkles. Scoring system provided by Zhao et al.^65^

Abbreviations: NLF, naso labial fold. Study type: CCT, controlled clinical trial; CS, case series; non‐R, nonrandomized; RCT, randomized controlled trial; spl‐F, split‐FACE. Centrifuge data: *g*, *g*‐force; rpm, revolutions/rotations per minute. Outcome: APC preparation: AC, Anticoagulant; ACD, acid citrate dextrose; CaCl, calcium chloride. Administration: HA, hyaluronic acid; ID, intradermal; MN, microneedling; NS, normal saline. Results: SS, Statistically significant; NS, Non Statistically significant.

In total, 5 studies investigated PRP as monotherapy,^49^, ^50^, ^51^, ^52^ 3 of these were compared to a control group of normal saline. In 4 of these studies, PRP was administered by intradermal injections and in one, by microneedling.^66^ All studies showed that PRP injections were significantly effective in improving skin conditions from photoaging, except for Alam et al.,^51^ where the difference between saline and PRP was not significant. There was no standardised assessment score across all trials and differing assement tools were used, namely Visia skin analysis, biopsy and patient assessment.

Histopathology was done in 5 studies and all showed changes such as increased dermal thickness, neocollagenosis, increased collagen volume, enhanced collagen organization, and increased fibroblasts (Table 3). Wrinkle assessment showed at least some improvement in most studies, however data did not support a lasting effect. Adverse events were mild and included pain, erythema, burning sensation and bruising.

The studies reviewed indicated that PRP was generally well tolerated and demonstrated efficacy for fine wrinkling and color homogeneity in photoaged skin. PRP treatment regimens for skin rejuvenation remain non standardized, with variable dosing and numbers of treatment sessions. Further large, double‐blind randomized controlled trials (RCTs) with standardized outcome measures are warranted to help optimize this potentially useful treatment approach to skin rejuvenation. Protocols may be a limiting reason for the variability in the findings.

Totally 3 studies investigated PRP as an adjunct therapy to other cosmetic treatments including fractional radiofrequency (RF) microneedling,^53^ nonablative laser^55^ and lipofilling. In all cases, the association was reported to provide beneficial results. Combining PRP with RF microneedling significantly improved cutaneous spots, wrinkles, pore reduction, and texture with great patient satisfaction. In another study, the combination of HA plus PRP was better than either substance alone with a highly significant improvement in facial appearance and skin elasticity, showing a new promising combination for injectable use. Willemsen et al.^57^ showed with lipofilling that although the PRP group had faster recovery time, there was no improvement over saline in the outcome of facial lipofilling when investigating skin elasticity improvement, or graft volume maintenance in the nasolabial fold. The role of PRP with fat grafting remains an area of clinical interest and investigation.

PRP was also compared to irradiated amniotic collagen matrix (IACM),^54^ and although both resulted in significant improvements in skin rejuvenation, better results were observed with IACM than with PRP. Further head‐to‐head studies need to be conducted with new regenerative substances. Cost effectiveness should also be considered. One of the ongoing challenges in establishing PRP as an adjunct in regenerative medicine remains the lack of standard dosing, outcome measures, and controls.

Totally 6 studies investigated PRF^45^, ^58^, ^60^, ^61^, ^62^, ^63^ as a stand‐alone cosmetic treatment, one compared PRF to normal saline in a split face study and 2 studies investigated PRF as a combination treatment with nanofat compared to HA, and as a combination with both nanofat vs autologous fat. Intradermal PRF application showed great potential for dermal restructuring and increased dermal thickness. Furthermore, this approach presents an accessible, cost‐effective, and alternative for facial rejuvenation. Patient satisfaction rates were high.

Combining nano‐fat with PRF gave greater patient satisfaction and the study concluded that PRF injection plus nano fat is a safe, highly effective, and long‐lasting method for skin rejuvenation.^59^ Further multicentre, controlled, and randomized studies with larger sample sizes are required to fully investigate the effects of PRF in photoaging and facial rejuvenation.

Only 1 study aimed to compare PRP and PRF injection treatments for facial skin rejuvenation in terms of efficacy, patient satisfaction, and side effects.^64^ A significant, superiority of PRF over PRP was only evident for canthal smoothness and wrinkles at month 3. Other parameters were similar. The authors concluded that PRF may represent a viable alternative to PRP for skin rejuvenation. Advantages are easier, more standardized preparation, absence of anticoagulants, and possibly its sustained effect. Further large studies remain warranted.

### 7.3 Conclusion

**FIGURE 15:**
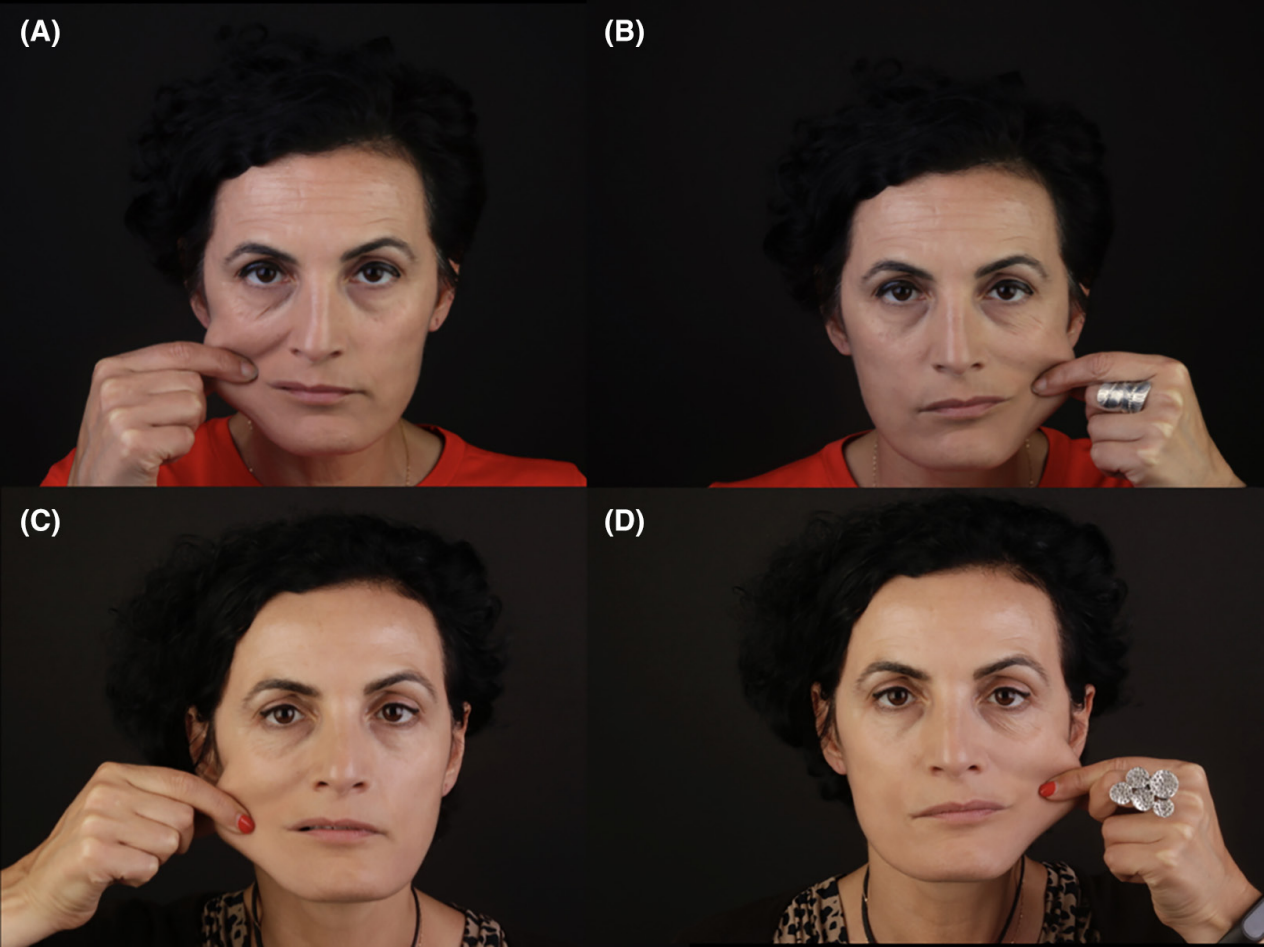
Before and after photo of a mid‐aged female patient having received 3 treatments with microneedling and intradermal injections with PRF. Case performed by Dr. Ana Paz. (A, B) Pre‐treatment skin pull test right and left. (C, D) Post‐treatment skin pull test right and left.

Among the noninvasive facial rejuvenation treatments, APCs have emerged as a promising therapeutic modality. Most studies have reported favorable outcomes in terms of improved skin texture, tone, and elasticity, as well as the mitigation of fine lines and wrinkles in aged skin (Figure 15, QR Code 3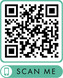). Treatments were generally well tolerated. Protocols for APCs in skin rejuvenation remain variable with regard to APC preparation, dosing and numbers of treatment sessions. Further high‐quality double‐blind RCTs with sufficient follow‐up and standardized outcome measures are warranted to help optimize this potentially useful treatment approach to skin rejuvenation. Slightly better outcomes with PRF injections when compared to PRP for facial rejuvenation in the only comparative study warrants further large studies comparing the two.

### 7.4 Clinical guidelines: skin rejuvenation

- Three treatments are usually required for adequate skin rejuvantaion spaced with a minimum 21 day interval. Therafter one maintenance treatment every 6 months may be required.
- Patients should be advised of a good home care routine and counseled on adequate sun protection and avoiding other damagimg habits such as smoking.

## 8 APC USE FOR MELASMA

### 8.1 Background

Melasma is an acquired pigmentary skin condition occurring most commonly on the face. This disorder, which is more prevalent in females and darker skin types, is predominantly attributed to ultraviolet (UV) exposure and hormonal influences, and can be esthetically displeasing to the patient. APCs have recently emerged as a novel treatment for melasma, but to date there has been no consistent evaluation of its efficacy or safety.^67^ Various topical, oral, and procedural therapies are used to treat melasma. Topical therapies may include hydroquinone, tretinoin, corticosteroids, and tranexamic acid. The Melasma Area and Severity Index (MASI) and the Modified Melasma Area and Severity Index (m‐MASI) is a validated scale used to measure the extent of facial hyperpigmentation. A formula is calculated and response to treatment can be assessed.^68^ The Melasma Severity Score has also been used.^69^

Quite a number of studies have reported promising results of APCs in the treatment of hyperpigmentation. The literature was searched for RCTs with the aim to study the efficacy and safety of APCs in the treatment of patients with melasma measured by an mMASI score reduction from f pre‐treatment compared to post‐treatment.

### 8.2 Outcome

A total of 9 studies^70^, ^71^, ^72^, ^73^, ^74^, ^75^, ^76^, ^77^, ^78^ were included in the analysis (Table 4). Four studies^70^, ^71^, ^74^, ^78^ evaluated the efficacy of PRP in melasma either alone or as compared to normal saline. A significant reduction in mMASI score from pre‐treatment to post‐treatment was observed in all studies. The overall efficacy evaluation of PRP showed that patients had a high degree of satisfaction with the treatment of melasma. Hofny et al.^78^ concluded that microneedling was more effective than intradermal PRP injections for administration method. This pilot study also showed that before treatment with PRP, the expression of TGF‐beta protein in the skins of melasma patients were significantly lower than that in the healthy skins. After treatment with PRP, the expression of TGF‐beta protein was significantly increased in the skin of melasma patients.^78^

**TABLE 4:** Intradermal APC injections for melasma.

Author	Study type	Subjects Gender Age % Smokers	Centrifuge rpm/min *g* force Anticoagulant	Treatment protocol and follow‐up	Outcome *Grading System*
APCs for intradermal injection melasma					
Sirithanabadeekul et al.^70^	*RCT* S*pl‐f*	10 patients ♀ = 10 Age: 18–65 years Melasma	Single centrifugation via YCELLBIO Kit® 3200 rpm @ 4 min AC used	Four treatment sessions every 2 weeks and A: PRP injected intradermally. B: NS injected intradermally FU 1 month after last treatment.	FU 1 month after last treatment. mMasi score A: Before 4.92 ± 0.96 After 3.5 ± 0.67 B: Before 4.98 ± 0.86 After 4.53 ± 0.96 Antera® 3D showed significant improvement in melanin levels in the PRP condition compared to the control condition Mexameter®Erythema and melanin indices did not significantly differ between the control and PRP conditions. PRP condition showed decreased pigmentation Patient satisfaction improved significantly in the PRP group and no change in the control group Final conclusion: PRP injection significantly improved melasma within 6 weeks of treatment in terms of mMASI scores
Acar et al.^79^	*CCT*	15 patients ♀ = 15 Age: 28–52 years Melasma	Double spin 1200 *g* 5 min then 1200 *g* @ 10 min Easy PRP® kit was used AC used	Intradermal PRP injections Every 3 weeks for 3 times	MASI Maxemeter (including MI and EI) done at baseline and 1 month after last treatment Mean MASI reduced from 10 ± 3.6 to 7.3 ± 2.5. Melanin index (MI) (mexameter) reduced from 256.5 ± 31 to 238.9. Mean patients' self‐assessment score (PSS) reduced from 8.3 ± 1.3 to 5 ± 1.4—significant Mean MASI, MI, and PSS reduction after treatment was found significant (*p* = 0.001, *p* = 0.000, *p* = 0.000). Final conclusion: Intradermal application of PRP is an effective option for treatment of melasma
Zhang et al.^72^	*RCT*	80 patients ♀ = 10/♂ = 70 Age: 24–35 years Melasma	Study group 3500 rpm 10 min Stood for 5 min then inverted and then PRP extracted	Control group (38 pts) simple oral TXA treatment 250 mg PO BD × 3 months Study group (42 pts) PRP combined with oral TXA treatment PRP monthly × 3 months + 250 mg TXA BD PO × 3 months	MASI score We classified patients with decreases of the MASI >90% as having a successful treatment 90%–60% having a moderate improvement 60%–20% as showing mild improvement <20% unsuccessful treatment Recurrance rate: 3 months *t*—disease recurrence rates were similar in both groups 6 months—disease recurrence rate of the study group was lower than that of the control group Total efficacy of the study group (90.48%) was higher than that of the control group (73.68%) (*p* < 0.05). Serum biochemical index levels: levels of VEGF were higher and the levels of ET‐1 and MSH were lower in the study group compared to the control group (*p* < 0.05) Final conclusion: PRP combined with oral TXA can improve the treatment effect of TXA alone in the treatment of melasma, maintaining normal levels of VEGF, ET‐1 and MSH, reducing disease recurrences
Patil et al.^73^	*RCT*	40 patients ♀ = 10/♂ = 30 Age: 21–54 years Melasma	Group B 685 *g* 5 min AC used	Once a month for five treatment sessions Group A TXA intradermal microinjections 1 cm apart 4 mg/mL TXA Max dosage 16 mg Group B PRP intradermal microinjections 1 cm apart	Clinical photographs every 4 weeks MASI and mMASI Group A, mean reduction of MASI and mMASI from baseline to end point was 6.572 ± 4.528 and 4.211 ± 2.647, Group B, mean reduction of both scores at the end of treatment reflected values of 8.167 ± 4.975(MASI) and 5.06 ± 2.977 (mMASI). Final conclusion: Statistically significant reduction in scores in both groups. No statistical difference between the 2 groups (although the effect of PRP was found to be better but no statistical significance)
Tuknayat et al.^74^	*CCT*	40 patients ♀ = 40/♂ = 0 Age: 24–52 years	(1) Double centrifugation (2) 10 min 1600 rpm (3) 10 min 4000 rpm	(1) Used PRP alone (2) Intradermal PRP injections Three sessions (4‐week intervals)	FU 3 months mMasi score Av decrease of 54% Patient Satisfaction. >90% were pleased or very pleased with results Final conclusion: PRP is effective as an adjuvant therapy but also as a standalone treatment for melasma. *n* addition to this, PRP also has an additional benefit of inducing collagen synthesis thus improving the quality and texture of the skin
Abd Elraouf et al.^75^	*CCT* *Spl‐f* Melasma	40 patients ♀ = 39/♂ = 1 Age: 28–52 years	R Side–TXA 100 mg/mL L‐PRP Double spin: 3000 rpm 7 min then 4000 rpm 5 min Activate: CaCl_2_ AC – sodium citrate right side‐TXA	Both sides had three treatment sessions monthly Then followed up 3 months after the last treatment. LHS: PRP intradermal injection RHS: 4 mg of diluted it with saline in 1 mL 0.05 mL was injected intradermally. into the lesions at 1 cm intervals, maximum 8 mg	Digital photographs At baseline and 3 months after last treatment mMasi Done before and after treatment across both sides of the face No statistical significance between the 2 sides before therapy After treatment high statistically significant decrease in the mMASI score after treatment on both sides, but higher score reduction in PRP vs. TXA. FU after 3 months: the mean mMASI score in the TXA‐side was 2.49 ± 1.58 with a mean percentage of decrease of 45.67 ± 8.10%, PRP side, the mean mMASI score after treatment was 2.17 ± 1.41 with a mean percentage of decrease of 53.66 ± 11.27%. There was a high statistically significant decrease in the mMASI score after treatment on both sides (*p* < 0.001); however, the percentage of score reduction in the PRP side compared to the TXA side was statistically higher Final conclusion: Intradermal injection with PRP revealed higher efficacy in the treatment of melasma as compared to TXA injection with no significant difference regarding the associated side effects
Mumtaz et al.^76^	*NRCT*	64 patients ♂ = 35 ♀ = 29 Age: 20–40 years Melasma	Group A Double spin 1500 rpm 10 min then 4000 rpm 10 min Activated with CaCl Ac used	Every 4 weeks for 12 weeks Group A 1 mL intradermal PRP Group B 4 mg intradermal TXA 1 mL (0.04 mL of TXA and the rest normal saline)	Final outcome measured at 24 weeks MASI score Statistically significant difference in scores for both groups from baseline to post‐treatment Mean MASI score at Baseline A 29.84 ± 5.14 vs. B 29.56 ± 4.39, (*p* = 0.21) 4 weeks A; 29.44 ± 5.35 vs. B 28.69 ± 4.10, (*p* = 0.01) 12 weeks A; 12.81 ± 1.78 vs. B; 18.38 ± 3.50, (*p* = 00001) 24 weeks A: 8.72 ± 3.40 vs. B: 14.97 ± 4.33, (*p* = 0.02) Significantly better results seen with Intradermal PRP than intradermal TXA Final conclusion: PRP is significantly better than intradermal tranexamic acid in management of melasma
Gamea et al.^77^	*RCCT*	40 patients ♀ = 40/♂ = 0 Age: 34–58 years Melasma	Group B Double spin 2000 rpm 3 min 5000 rpm 5 min Activated with CaCl AC used	Group A Topical TXA 5% liposome cream BD × 12 weeks Group B Topical TXA 5% cream BD × 12 weeks With PRP every 3 weeks throughout the treatment (4 sessions)	mMASI Both groups showed significant reduction in score Significantly better treatment response/improvement in mMASI score in group B (*p* = 0.024). Patient satisfaction and response 1 month after the last treatment, better in group B (*p* = 029). Dermascopic evaluation to confirm diagnosis and type of melasma Final conclusion: PRP is a autologous, safe elixir which boosts the therapeutic effect of TXA cream, yielding better results together. PRP is advisable as an autologous safe elixir which boosts the therapeutic effect of tranexamic acid
Hofny et al.^78^	*RCT* *Spl‐f*	23 patients ♂ = 4 ♀ = 19 Age: 21–50 years Melasma	PRP double spin 160 *g* 10 min 400 *g* 10 min Activator: CaCl_2_	Group A: MN PRP one side Group B: ID injection PRP Three session, 4 weeks intervals FU 1 month after last treatment	MASI score A: Baseline 11.86 ± 5.25, After: 6.96 ± 4.82 B: Baseline 5.71 ± 2.56, After: 2.90 ± 2.05 Final conclusion:Both groups of patiemts improved Pilot study shows increased TGF‐beta protein expression in skin of melasma patients after PRP treatment. The alterations of TGF‐beta protein in skin of melasma patients not only support its use as a therapeutic option in melasma

Abbreviations: Study type: CCT, controlled clinical trial; CS, case series; non‐R, nonrandomized; RCT, randomized controlled trial; spl‐F, split‐FACE. Centrifuge data: *g*, *g*‐force; rpm, revolutions/rotations per minute. Outcome: APC preparation: AC, Anticoagulant; ACD, acid citrate dextrose; CaCl, calcium chloride. Administration; HA, hyaluronic acid; ID, intradermal; MN, microneedling; TXA, tranexamic acid. Assessment: *MASI*, Melasma Area and Severity Index (Score); mMASI, modified Melasma Area and Severity Index (Score); NS, normal saline. Study results: NS, Non Statistically significant; SS, Statistically significant.

A total of five studies^72^, ^73^, ^75^, ^76^, ^77^ aimed to compare the clinical results of tranexamic acid (TXA) and PRP therapies in patients with melasma. Studies showed that both TXA and PRP were effective at improving melasma in terms of mMasi scores. Intradermal injection with PRP revealed higher efficacy in the treatment of melasma as compared to TXA injection with no significant difference regarding the associated side effects. This finding was significant in all studies except for Patil et al.^73^ who found the difference nonsignificant.

The research validated that PRP is a safe and efficient treatment for melasma. Used both alone and in combination therapy, PRP treatment achieved a significant reduction of the MASI or mMASI score.

### 8.3 Conclusion

These results support the efficacy and safety of APCs used either alone or in combination with synergistic effects such as various topical cream formulations.

### 8.4 Clinical guidelines

APC use in melasma works best with an effective home care routine as well as adequate sun protection. Melasma should be managed as a chronic condition with regular follow ups.

## 9 APC USE FOR VITILIGO

### 9.1 Background

Vitiligo is a skin condition characterized by melanocyte destruction leading to depigmented or hypopigmented macules and patches. The disease may significantly impair quality of life (QOL).^80^ Current treatment options offer limited response and has been attempted with a range of topical and systemic corticosteroids, calcineurin inhibitors, fractional CO_2_ lasers, narrow‐band ultraviolet B (NB‐UVB) phototherapy, and surgical transplantation of autologous melanocytes.

### 9.2 Outcome

Four studies have investigated the use of APCs for vitiligo all of which used a combination approach with laser therapies including narrow band UVB,^81^ excimer laser,^82^, ^83^ and fractional CO_2_ laser^84^ (Table 5).

**TABLE 5:** Intradermal APCs for vitilgo.

Author	Study type	Subjects Gender Age % Smokers	Centrifuge rpm/min *g* force Anticoagulant	Treatment protocol and follow‐up	Outcome *Grading System*
APCs for intradermal injection vitiligo					
Ibrahim et al.^81^	*CCT* *Spl‐b*	60 patients ♀ = 34/♂ = 26 Age: 18–35 years Non segmental vitiligo	PRP Double spin: 7 min 5 min AC: Sodium Citrate Activator: CaCl_2_	LHS: Narrowband UV B only RHS Narrowband UV B plus PRP every 2 weeks for 4 months	There was statistically highly significant improvement in the repigmentation in the combination group (PRP plus NB‐UVB) compared with NB‐UVB group. (*p* < 0.001) Final conclusion: lusion: Intradermal PRP injection in combination with NB‐UVB could be considered as a simple, safe, tolerable, and cheap technique for treatment of vitiligo. It shortens the duration of NB‐UVB therapy and is expected to increase patient compliance. Longer follow‐up is needed
Kadry et al.^84^	*RCT*	30 patients ♀ = 22/♂ = 8 Age: 18–59 years Non segmental vitiligo	PRP Regenlab kit 1500 rpm 5 min	4 Groups: Group 1: PRP group Group 2: CO_2_ group Group 3: CO_2_ plus PRP Group 4: Control	FU after 3 months VAS There was a statistically significant difference in repigmentation grade, MISP, and VAS among the combined Fr: CO_2_ with PRP group, the PRP group, and other groups (*p* < 0.001; Kruskal–Wallis test). VACAG was 57.01 in the PRP alone group, 54.22 in the CO_2_ and PRP combined group, 38.08 in the CO_2_ laser alone group, and 13.79 in the control group. MISP and VAS were highest in the CO_2_ and PRP group, and the PRP monotherapy group, with a MISP of 3.20 and 2.97, and VAS of 6.87 and 6.67 in the CO_2_ and PRP combination and PRP monotherapy groups, respectively (*p* < 0.001) Final conclusion: The combination CO_2_ laser and PRP, and PRP alone, had the highest improvement; Regarding the site, the most significant improvement was observed in the trunk followed by the face, the upper limb (UL), and the lower limb (LL), which was the most resistance site
Khattab et al.^82^	*RCT*	52 patients ♀ = 44/♂ = 8 Age: 18–40 years Non‐segmental symmetrical vitiligo	PRP Double spin 200 *g* 10 min 2000 *g* 15 min AC: trisodium citrate Activator CaCl_2_	2 Groups: Group 1: Intradermal PRP and 308 nm excimer laser (PRP every 3 weeks × 4 months) Group 2: 308 nm excimer laser only	FU 3 months after end of Rx VAS score: Group 1. four patients (15.4%) show no response, 13 patients (50%) show good response, and 9 patients (34.6%) show excellent response Group 2. 17 patients (65.4%) show no response, nine patients (34.6%) show good response, and no patient shows excellent response in the group II The combination group had higher repigmentation compared with excimer laser alone, with excellent (75%–100%) response in 34.6% of patients compared to 0% in the placebo group, and good (50%–75%) response in 50% compared to 34.6% in placebo (*p* = 000) Final conclusion: The combination of PRP and excimer laser phototherapy is an efficient vitiligo treatment as PRP increases the excimer laser impact and also improves the result
Deng et al.^83^	*RCT*	60 patients ♀ = 34/♂ = 26 Age: 18–65 years Condition: Localized, stable vitiligo	PRP Double spin: 1500 rpm 6 min 2500 rpm 15 min AC: sodium citrate Activator: CaCl	Three groups Group 1: Intradermal PRP Group 2: 308 nm excimer laser 33. Group 3: 308 nm excimer laser plus PRP injected 30 min before session	FU 3 months after Rx Vitiligo disease activity (VIDA) score: The laser plus PRP group had an increase in repigmentation with a total effective rate of 80% compared to 25% in the PRP alone group and 35% in the laser alone group (*p* < 0.05 for both), but no significant difference between the PRP and laser groups VASI score: showed significant differences among the three groups (*p* < 0.001), with the highest score in group III, followed by group II and then group I Repigmentation responses also showed significant differences among the groups (*p* < 0.001), and the best effect was observed in group III No side effects were reported in any of the groups Final conclusion: The effect of PRP combined with 308‐nm excimer laser on stable vitiligo is significantly better than that of PRP and 308‐nm excimer laser alone. It is safe and satisfactorily tolerant

Abbreviations: Study type: CCT, controlled clinical trial; CS, case series; non‐R, nonrandomized; RCCS, Randomized case control study; RCT, randomized controlled trial; retro, retrospective; spl‐m, split‐mouth. Centrifuge data: *g*, *g*‐force; rpm, revolutions/rotations per minute. Assessment: MISP, mean improvement score by physician; VACAG, Vitiligo analysis by computer‐assisted grid; VASI, Vitiligo Area Scoring Index—clinician‐reported outcome measure^85^; Wood's lamp—visual assessment, VIDA, Vitiligo disease activity (VIDA) score. Study Results: NS, Non Statistically significant; SS, Statistically significant.

The most utilized clinician‐reported outcome measure was the VASI score (Vitiligo Area Scoring Index), measuring repigmentation both before and after treatment. There was highly statistically significant improvement in the repigmentation in the combination groups of all four studies, demonstrating that PRP increased the impact of each of the excimer laser, CO_2_ laser and narrow‐band UVB.

### 9.3 Conclusion

APCs are effective either as an adjunct therapeutic or alternative therapeutic option for patients with conditions like vitiligo. Treatments are more effective on the trunk and face than the rest of the body. It appears the use of energy‐based devices in combination with APCs are preferred over other methods. Additional studies are required to further validate these findings.

### 9.4 Clinical recomendations

Multi modal treatments are best for patients with vitiligo, with adequate UV protection for depigmented areas. Counseling should be offered to patients regarding psycho social stresses related to the condition.

## 10 USE OF APCs FOR STRIAE/STRETCH MARKS

### 10.1 Background

Striae distensae (SD), also known as stretch marks, are esthetically displeasing linear bands of benign dermal lesions, with flattening atrophy of the epidermis.^86^ They are particularly associated with female sex, weight gain, pregnancy, and/or hormonal change. They histologically resemble dermal scars.^86^

SD are therapeutically challenging and based on previous studies, current treatment modalities have only shown modest SD improvement, and large, controlled studies are lacking.^87^ Management of SD includes microneedling, microdermabrasion, pulsed dye lasers, RF, carboxytherapy and both non ablative and ablative fractionated lasers.

Clinical trials on APCs and their effect on striae distensae are scarce and their level of evidence mostly poor. The literature was reviewed by investigating the published literature on APCs as an adjuvant to current treatment modalities in treating skin stretchmarks. There is no use of a standardized assessment scale for the subjective evaluation of SD.

### 10.2 Outcome

A total of 6 studies assessed the use of PRP for the treatment of stretchmarks^88^, ^89^, ^90^, ^91^, ^92^, ^93^ both alone and as an adjunct to microneedling,^88^ intradermal RF,^89^ carboxytherapy,^93^ pulsed dye laser (PDL),^90^ CO_2_ laser,^90^, ^91^ as well as microdermabrasion^92^ (Table 6). Results showed that both subjectively and objectively there was a significant improvement of the appearance of stretch marks treated when combining APCs with other modalities, compared to that same modality alone. Histopathological improvement of epidermal atrophy, increase in epidermal thickness, and an increase in rete ridges formation with a decrease in perivascular inflammatory infiltrate were seen after APC use.

**TABLE 6:** Intradermal APCs for striae distensae/alba.

Author	Study type	Subjects Gender Age Condition % Smokers	Centrifuge rpm/min *g* force Anticoagulant	Treatment protocol and follow‐up	Outcome *Grading System*
APCs for striae					
Abdel et al.^88^	*RCT*	40 patients ♀ = 38/♂ = 2 Age: 15–42 years Striae alba	PRP filtering using system's sleeve filter AC: ACD Activator CaCl_2_	Three sessions, 1 month interval Group I: MN with PRP Group II: MN only	*FU after 3rd session* Photo: SS improvement of the skin lesions of SD was found following the application of combined MN with PRP vs. MN alone. SS (*p*‐value <0.001) Biopsy: Treatment with combined MN with PRP was associated with a statistically significant collagen and elastin fibers deposition (*p* < 0.000) as compared to MN only Final conclusion: MN‐PRP is more effective than MN alone for the treatment of the lesions of SD
Kim et al.^89^	*CCT*	19 patients ♀ = 19 Age: 19–43 years Striae distensae	PRP MyCells device 1200 *g* 7 min AC: ACD Activator: Thrombin filtered using the sleeve filter	Three sessions: 4 weeks interval intradermal RF plus PRP	FU after 4 weeks Histology: collagen and elastin fibers increased with incr collagen density subepidermally. Clinical results: 5.3%—excellent improvement, 36.8%—marked improvement, 31.6%—moderate improvement, 26.3%—mild improvement. A total of 63.2% of patients reported they were “satisfied” or “very satisfied” with the degree of overall improvement Final conclusion: Intradermal RF combined with autologous PRP appears to be an effective treatment for striae distensae
Neinaa et al.^90^	*RCT* *Spl‐b*	30 patients ♀ = 30 Striae distensae	: Rotofix 32 Double spin 1419 *g* 7 min/2522 *g* 5 min AC: sodium citrate Activator: CaCl_2_	Three session 6 weeks interval RHS: intradermal PRP injection followed by (Fr CO (_2_) laser on right side and PDL on left side). LHS: intradermal PRP injection followed by PDL (Pulse dyed laser)	FU 1 month after last session Both treatment sides reported significant clinical improvements of SD lesions, reduction of width of striae lesions – mprovement of skin texture (significant). Higher degree of clinical improvements combined PRP with Fr CO (2) laser rather than combined PRP with PDL. Histopathologically, SD lesions showed improvement of epidermal thickness, and more homogenization and regular orientation of dermal collagen fibers (both sides), with more significant improvement on the side treated by combined PRP with Fr CO (_2_) laser sessions rather than the other side Final conclusion: PRP injection in combination with Fr CO (_2_) laser or PDL is a safe and effective therapeutic regimens for SD. However, its combination with Fr CO (_2_) laser is more promising with better outcome and fewer side effects
Sayed et al.^91^	*RCT* *Spl‐b*	30 patients ♀ = 30 Age: 16–27 years Striae distensae	160 *g* RCF 10 min 400 *g* RCF 10 min AC: sodium citrate Activator: CaCl_2_	Group A: Combined fractional CO_2_ laser with intradermal PRP Group B: fractional CO_2_ laser alone	Biopsy before and after treatment Group A, a significant excellent improvement of the SD was achieved more than in group B (*p* 5 0.007) and the mean of improvement was significantly higher (60.33 ± 26.49) than that in group B (43.80 ± 27.43) (*p*‐value 5 0.001) Group A was also associated with a more significant dermal deposition of collagen and elastic fibers Final conclusion: The study showed that combined fractional CO_2_ laser with intradermal PRP had more therapeutic effect on SD than fractional CO_2_ laser alone, without serious side effects
Zeinab et al.^92^	*RCT*	68 patients ♀ = 54/♂ = 14 Age: 14–40 years Striae distensae	1419 *g* 7 min (soft spin) 2522 *g* 5 min (hard spin)	Six session 2 weeks interval Group I: intradermal injection of PRP Group II: microdermabrasion alone Group III were treated with combination of intradermal PRP and microdermabrasion	FU end of 3 months Histology ↑: epidermal thickness, rete ridge formation, elastic fibers (thicker, longer, and more evenly arranged)↓: perivascular infiltrate (“especially” after PRP) Photography: There was significant clinical improvement of SD in patients treated with PRP injection and patients treated with combination of PRP and microdermabrasion when compared with patients treated with microdermabrasion. Combination PRP and microdermabrasion – better results in short duration Final conclusion: PRP alone is more effective than microdermabrasion alone but better to use the combination of both for more and rapid efficacy
Hodeib et al.^93^	*RCT* *Spl‐b*	20 patients ♀ = 14/♂ = 6 Age: 17–40 years Striae alba	PRP double spin: 1409 *g* 7 min (soft spin) 2504 *g* 5 min (hard spin)	Group A: PRP injection (RHS) Group B: carboxytherapy (LHS) left side Four sessions every 3–4 weeks for	FU after 3 months There was a significant improvement in SA in both groups after than before treatment. No significant difference between A and B: percentage of improvement, response (grading scale), or patient satisfaction. The fibronectin‐stained area was significantly higher in both groups after than before treatment, and it was significantly higher after treatment in group (B) than group (A) Final conclusion: Both methods were safe and effective with minimal side effects

Abbreviations: Study type: CCT, controlled clinical trial; CS, case series; non‐R, nonrandomized; RCT, randomized controlled trial; spl‐b, split‐body; Conditions: SA, straie alba; SD, straie distensae; Centrifuge data: *g*, *g*‐force; rpm, revolutions/rotations per minute; Outcome: AC, Anticoagulant; ACD, acid citrate dextrose; APC preparation: CaCl, calcium chloride; Administration: HA, hyaluronic acid; ID, intradermal; MN, microneedling; Assessment: NS, Non Statistically significant; SS, Statistically significant.

Neinaa et al.^90^ compared two treatment modalities: CO_2_ laser on right side and PDL on the left side, both combined with PRP injections. The side with a combination of PRP and CO_2_ laser proved to be more promising and had better patient satisfaction and fewer side effects. Histology of the SD lesions showed improvement in the epidermal thickness, and normalized orientation of dermal collagen fibers in both treatment groups, but there was more significant improvements on the side treated by combined PRP and CO_2_ laser.^90^

### 10.3 Conclusions

**FIGURE 16:**
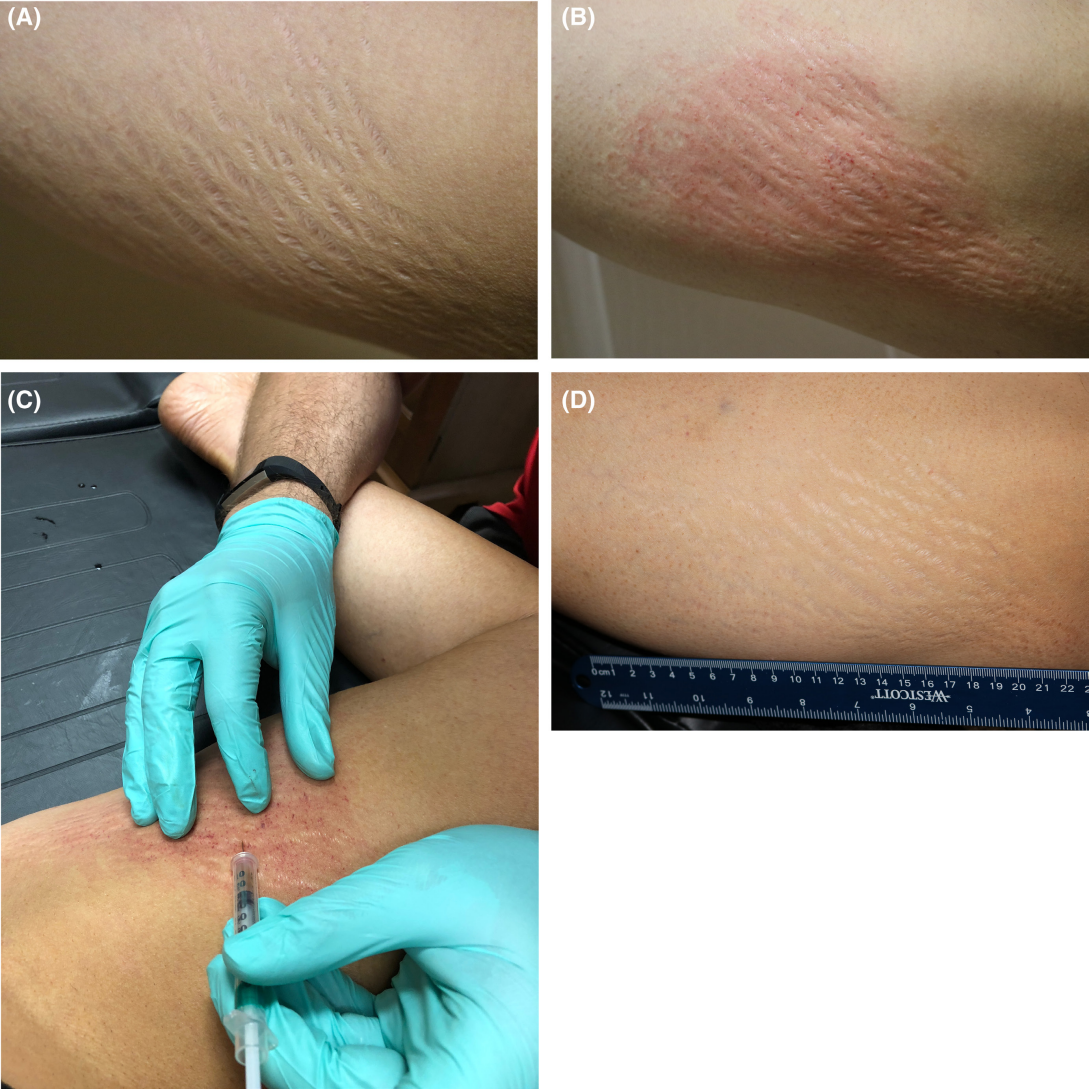
(A) Clinical image demonstrating substantial and pronounced stretch marks on the inner thighs. (B, C) This patient was treated with a combination of liquid PRF + micro‐needling as well as subcutaneous liquid‐PRF injection. (D) Note the clinical improvement following four treatment sessions, though the stretch marks were still apparent and did not reach complete resolution. Reprinted with permission from Davies/Miron.^8^

APCs may be effective in the management of striae, in particual when combined with other treatment modalities, confirmed on histology. The earlier treatment is initiated, the better the outcome. There seems to be much more variability in this space however positive outcomes lead to quite nice clinical outcomes and patient satisfaction (Figure 16).

### 10.4 Clinical recommendations

Patients should avoid further triggers of striae where possible, such as excessive weight gain or loss, or chronic use of corticosteroids.

## 11 APCs FOR PERIORBITAL REGENERATION

### 11.1 Background

The periorbital area is oftenone of the first to show signs of aging. This area is prone to pigmentation, crow's feet, tear‐trough hollowing, decreased skin elasticity, and melanosis, all of which reveal a person's age. Addressing these areas by treating fine lines, wrinkles, and volume loss can benefit the patient. The skin in this region is thin and delicate. Thus, caution needs to be taken when administering treatment and considering the proximity to the eye itself. Common side effects include swelling and bruising.

The periorbital area is a common target for facial esthetics because this is one of the areas where signs of aging are seen the earliest. Dark circles, known as periorbital hyperpigmentation (POH), periocular wrinkles (crow's feet) are two common signs of aging in this region.^94^ Loss of volume, known as tear trough hollowing is also a cosmetic concern in this area and is discussed later in this chapter under “APCs in volumising.”

Established treatment options, such as hyaluronic acid injections, botulinum toxin injections, microneedling, skin resurfacing (microdermabrasion), chemical peel (exfoliation), laser treatment, as well as blepharoplasties and autologous fat transfers, are available and can be successful but are also associated with high costs, downtime and some risks^95^

APCs have previously been used in the rejuvenation of the periorbital areas and may be administered by microneedling, at a depth of 0.25 mm or intradermal papule injection to the eye area and crow's feet. APCs have been used as a nonsurgical option to rejuvenate the peri‐orbital area pathologies of wrinkles, periorbital hyperpigmentation (POH), and photoaging to evaluate the use of APCs in the treatment of periorbital aging.

### 11.2 Outcomes

In total 9 studies were reviewed, 7 on the use of PRP in the periorbital area^95^, ^96^, ^97^, ^98^, ^99^, ^100^, ^101^ and 2 on PRF.^102^, ^103^ One of the studies compared PRP to PRP gel.^101^ Three studies evaluated the effect on only, and the remainder on both POH and crow's feet (Table 7).

**TABLE 7:** APCs for periorbital rejuvenation.

Author	Study type	Subjects Gender Age % Smokers	Centrifuge rpm/min *g* force Anticoagulant	Treatment protocol and follow‐up	Outcome *Grading System*
PRP for peri‐orbital skin regeneration					
Aust et al.^95^	*CT*	20 patients ♀ = 16/♂ = 4 Age: 21–60 years Actinic elastosis	Arthrex ACP double syringe No AC	Three sessions 1 month interval PRP via cannula injection	FU 1 month after last Rx High level of patient satisfaction Cutometer: SS higher level of skin firmness (due to increased collagen production) SS increase in skin elasticity Final conclusion: A series of PRP injections in the kower eyelid region are a safe and effective treatment option
Budania^96^	*RCT spl‐f*	21 patients ♀ = 21/♂ = 0 Age: 18–50 years Condition: POH	Two methods Group A: Single spin and low‐temperature activation (novel) 100 *g* 10 min Group B: Double spin and calcium activation (conventional) AC: Sodium citrate Activator: calcium gluconate 10%	Group A: RHS‐novel PRP Group B: LHS conventional PRP Three PRP injections were given at 1 month intervals	FU week 12 Photography & Dermoscopy Group A: 52.33 ± 6.468 Group B: 53.14 ± 6.99 (*p* = 0.151). Mean improvement in both groups >50% Final conclusion: A high success rate of PRP (both novel and conventional) in managing POH
Ellabban et al.^97^	*RCT*	42 patients ♀ = 38/♂ = 4 Age: 25–32 years Condition: POH	PRP Double spin 150 *g* 5 min 2000 *g* 12 min AC: trisodium citrate Activator CaCl_2_	Group A: sessions chemical peeling (trichloroacetic acid and lactic acid) TCA 3.75% and lactic acid (LA) 15% Group B: 4 sessions of PRP injection with 2 weeks of intervals	FU after 3 months Significant improvement in favor of chemical peeling over PRP Good improvement occurred in 47.6% in the group A vs. 4.8% in group B (*p* < 0.001). None of the PRP group had excellent improvement, 38% of chemical peeling group did Final conclusion: Both PRP and chemical peeling are effective for treatment of POH; however, chemical peeling is much more effective, tolerable, and satisfying procedure than PRP
Mehryan et al.^98^	*RCT spl‐f*	10 patients ♀ = 10/♂ = 0 Age: 26–61 years Condition: Crow's feet & POH	PRP Selex centrifuge device. Double spin: 1800 *g* 6 min/2000 *g* 5 min Activator: CaCl_2_ AC; ACD	Single session with intradermal injections of 1.5 mL PRP into tear trough area and crow's feet wrinkles on each side	FU after 6 months 80% achieved fair to good improvements after 3 months SS improvement in POH (*p* = 0.010), in all patients Final conclusion: PRP as potential therapeutic modality in treating POH
Nofal et al.^99^	*CCT*	80 patients ♀ = 26/♂ = 4 Age: 20–44 years Condition: POH	Double spin method: 150–200 *g* 10 min/1500 2000 *g* 15 min AC: Trisodium citrate Activator: CaCl_2_	R eye: carboxytherapy on the right area with 1 week interval apart. L eye: seven intradermal injections of PRP on the left periorbital area 2 weeks intervals	10 patients dropped out – PRP side effects SS improvement in POH was achieved in both sides (*p* ≤ 0.0001) The improvement was comparable with no SS difference between both modalities Final conclusion: Both PRP and carboxytherapy are relatively effective and Carboxtherapy is simple and slightly more effective modality and well tolerated than PRP
Banihashemi et al.^100^	*CCT*	32 patients ♀ = 32 Age: 35–55 years Glogau 2–5 Crow's feet & POH	Double spin 2000 *g* 2 min and 4000 *g* 8 min AC: Heparin Activator: calcium gluconate	Two sessions PRP injected 3 months apart Point by point intradermal injections Periorbital and NLF	FU 3 and 6 months improvement in periorbital dark circles (47.8, 60.9%), periorbital wrinkles (73.9%, 78.3%) Final conclusion: Face rejuvenation with PRP is a promising and noninvasive technique with best results observed in improving periorbital dark circles and wrinkles
Diab et al.^101^	*CCT spl‐f*	40 patients ♀ = 40/♂ = 0 Age: 21–40 years Condition: crow's feet & POH	PRP: 320 *g* 15 min/1000 *g* 5 min Plasma gel with PPP: hot water bath (60–100°C) – 1 min, cold bath at (8 and 0°C) – 1 min AC: ACD Activator: calcium gluconate	PRP vs. plasma gel: Two session 1 month apart RHS intradermal PRP LHS intradermal plasma gel	FU 16 weeks GAIS and Antera Two sessions of both PRP and plasma gel are effective for periorbital rejuvenation, with plasma gel showing significantly better results Final conclusion: Improvement was not maintained for 3 months for two sessions
PRF for peri‐orbital skin regeneration					
Majewska^103^	*RT*	10 patients ♀ = 10/♂ = 0 Age: 32–45 years Condition: crow's feet thinning of skin	PRF 60 *g* 3 min No AC	PRF intradermal injection—four sessions 1 month apart	FU 1 month after last Rx *DUB SkinScanner* vs. baseline After second 1.66 × higher After the third 5.08 × higher *VAS scale* the Av score baseline vs after: 4 up to: 8.5 (SS) Final conclusion: PRF is an effective treatment modality for skin rejuvenation in the periorbital area for those seeking natural treatments
Mahmoodabadi et al.^102^	*CCT*	16 patients ♀ = 8/♂ = 8 Age: 28–62 years Smokers 0% Condition: crow's feet & POH	PRF 700 rpm 5 min No AC used	Injection with 27 Gauge cannula around eyes, sub dermis once Assessed week 0 week 12	FU after 3 months Visophysis device Noticeable improvement in deep, fine, and small wrinkles, periocular hyperpigmentation, and overall skin freshness of the injection site Final conclusion: PRFM was observed to have potential in skin rejuvenation, promising outcomes in terms of safety and long‐term effects in improving skin condition

Abbreviations: Study type: CCT, controlled clinical trial; CS, case series; non‐R, nonrandomized; RCT, randomized controlled trial; spl‐F, split‐face. Centrifuge data: *g*, *g*‐force; rpm, revolutions/rotations per minute. Outcome: AC, Anticoagulant; ACD, acid citrate dextrose; APC preparation, CaCl, calcium chloride. Administration: HA, hyaluronic acid; ID, intradermal; MN, microneedling. Assessment: GAIS, Global Esthetic Improvement score; POH, periorbital hyperpigmentation. Results: NS, Non Statistically significant; SS, Statistically significant.

Only a few controlled studies have been performed on the use of PRP on POH. Evaluating patient satisfaction in these studies varies greatly, however it seem that PRP has great potential as an effective and safe treatment for POH, with high patient satisfaction rates, as demonstrated by numerous studies.^96^, ^97^, ^98^, ^99^, ^100^

Histologic and patient satisfaction scores showed that PRP treatment has promise for the applications of periorbital wrinkles. Most studies showed improvement in skin thickness and a significant improvement in periocular wrinkles,^95^, ^99^, ^100^, ^101^ except for an early study by Mehryan et al,^98^ that showed a significant improvement in POH only. Although the pool of evidence is low it seems that PRP, although effective is less preferred when it comes to POH than chemical peels^97^ or carboxytherapy.^99^

Diab et al. demonstrated that 2 sessions of both PRP and plasma gel were both effective for periorbital rejuvenation, with plasma gel showing significantly better results. Interestingly, when looking at subject age in the study of treatment efficacy in photoaging 15% of patients were in their early twenties,^95^ which indicates that the lower eyelid region can be affected by signs of aging even in younger years. Another observation was the positive treatment response in the over 60 age group, which is striking, considering that the body's ability to regenerate declines with increasing age.

Of the two PRF studies, Mahmoodabadi et al.^102^ demonstrated a noticeable improvement in peri‐ocular wrinkles, both deep and fine, as well as improved periocular hyperpigmentation. PRF was observed to have potential in periorbital skin rejuvenation, demonstrating promising outcomes in terms of safety and long‐term effects in improving skin condition. Similarly, Majewska demonstrated an improvement in crow's feet and skin thickness (1.6 times) measured by a high frequency ultrasound device. They both concluded that the results of PRF on peri ocular aging are very promising. There remains a need for larger controlled research.

### 11.3 Conclusions

**FIGURE 17:**
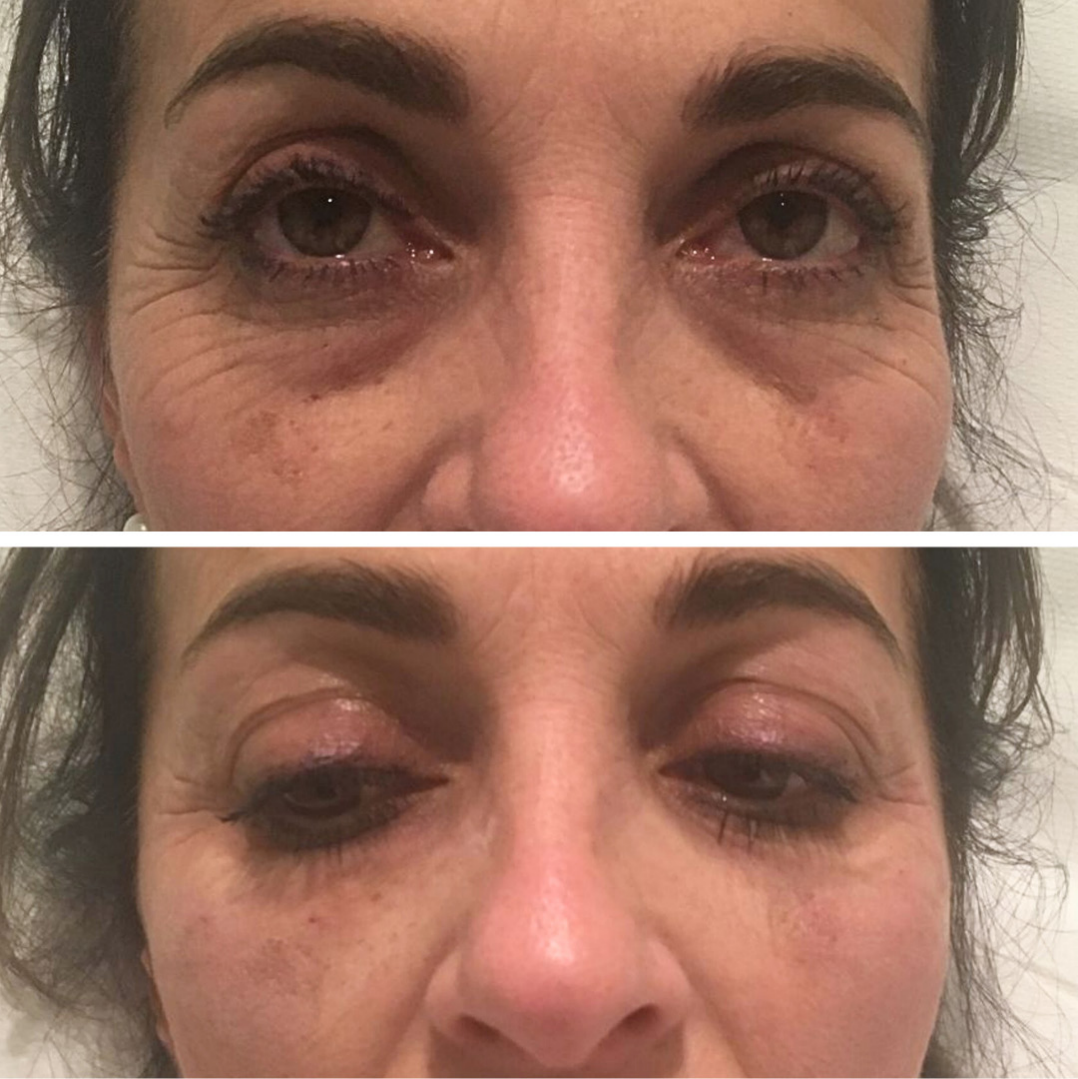
Before and after one session of under eye treatment with Alb‐PRF in a post‐menopausal female. Case performed by Dr. Catherine Davies.

In conclusion, the periocular area is a difficult area to treat due to its thin skin, constant blinking motion and proximity to the eye. Noteworthy, many injectors avoid the use of chemical fillers in the area altogether owing to their raised safety concerns. APCs are a promising option for skin rejuvenation in the periocular region, demonstrating satisfactory outcomes in terms of safety and in improving pigmentation and skin condition. Figure 17 demonstrates a case using PRF (in its Alb‐PRF formulation presented later in the article). Note the significant improvement in appearance from only 1 session when the peri‐orbital region is treated effectively. Further studies remain needed to determine the best way to administer APCs to the undereye area i.e., via intradermal injection, cannula or, microneedling. Studies combining APCs with other periocular treatments, such as neurotoxins and lasers are also needed.

### 11.4 Clinical recommendations

It is recommended to use small gauge needles (such as 30G), and lower the depth of microneedling devices (0.25 mm) when treating the delicate skin of the under eye area. If the novel Alb‐PRF is utilized, a 22G × 2 inches (~5 cm) cannula is recommended for safety around the eyes (QR Code 4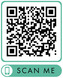). Patients should be warned about the potential of bruising and swelling, which is common when treating this area.

## 12 APCs FOR PERIORAL REGENERATION

### 12.1 Background

Lip augmentation is one of the most common procedures in esthetic medicine and has traditionally been performed using hyaluronic acid‐based fillers (HA fillers). APCs have been increasingly used for lip injections, yet limited research is available on these treatments' efficacy, safety, and longevity.^104^ The treatment goal of lip augmentation is to create harmonious volumizing of the lips, which are symmetrical and in line with the rest of the face. Patients may need lip volume, lip symmetry correction, lip contouring, or superficial lip regeneration. APCs have also been used to decrease perioral wrinkles by improving skin condition.

### 12.2 Outcome

Four studies assessed the use of APC in the lip and perioral area.^105^, ^106^, ^107^, ^108^ 3 involving PRP and one PRF (Table 8). Huang,^105^ Araco^107^ and Suhai^106^ studied the regenerative effect of PRP in the peri oral area. Interestingly the most promising evidence of PRP as an effective option on lip rejuvenation was color improvement They also demonstrated that PRP was an effective option for lip rejuvenation, yet longevity of the effects needs to be studied further. Added benefits were that PRP caused biostimulation of the lips and gave natural, esthetically pleasing results.^106^ There was also an improvement of dermal structure and moisture of the lips.^107^

**TABLE 8:** APCs for peri‐oral rejuvenation.

Author	Study type	Subjects Gender Age % Smokers	Centrifuge rpm/min *g* force Anticoagulant	Treatment protocol and follow‐up	Outcome *Grading System*
APCs for peri‐oral regeneration					
PRP for peri‐oral regeneration					
Huang et al.^105^	*CCT*	15 patients ♀ = 14/♂ = 1 Age: 27–58 years	Double spin 2200 *g* 4 min/2200 *g* 3 min TriCell PRP preparation device Activator: CaCl_2_	Microinjections of 0.15 mL‐point by point into lips	FU 3–24 months VISIA skin detector The most obvious improvement was that the color of the lips which became more vivid Three participants experienced mild pain or discomfort during the injection process. There was no swelling, bruising, scar hyperplasia and other complications Final conclusion: Promising evidence of PRP as an effective option on lip rejuvenation. However, large, multi‐center, controlled, long term, pilot studies are required to confirm the preliminary results of our study
Suha et al.^106^	*CCT*	15 patients ♀ = 14/♂ = 1 Age: 25–42 years Smokers 0% Perioral wrinkle	1700 *g* 5 min AC: ACD‐A Activator: CaCl_2_	Three sessions of monthly PRP injections targeting line and wrinkles in the perioral area	FU after third injection Wrinkle severity scale Patient's Satisfaction Score (PSS) final PSS measurement was 4.4 which is consistent with (good) result The degree of difference between baseline and FU was SS (*p* value = 0.01) Clinician's Satisfaction Score (CLSS): the average result was good (4.33) The difference between baseline and FU was significant, (*p* value = 0.03) Final conclusion: PRP is a safe, natural, cost‐effective substitute to other lip and perioral rejuvenation methods. It is a form of biostimulation that has an early effect with natural looking results. High patient's satisfaction was reported and results became more significant after the third session
Araco^107^	*CCT*	50 patients ♀ = 0.50/♂ = 0 Age: 39–59 years Perioral wrinkles	Plasma Active system 5 min 1800 rpm Activate by combining with 8 mL Medical Device (shaken)	Group 1: single session of fractional CO_2_ laser skin resurfacing plus intradermal PRP Applied topically prp bd‐12 weeks Group 2: 1 single session of fractional CO_2_ laser skin resurfacing plus topical gentamicin /betamethasone bd—7 days, then HA gel daily—12 weeks	FU after 12 weeks Results: In group 1, moisture (*p* < 0.001), amount of collagen fiber (*p* < 0.001) skin elasticity (*p* < 0.001), improved significantly. PSAl (*p* < 0.001) and SSAl (*p* < 0.001) improved significantly. Group 2 all the parameters investigated improved but did not reach significant difference Final conclusion: topical prp reduces superficial perioral wrinkles and restore dermal matrix when used at home for 12 weeks PRP significantly improves the moisture, amount of collagen fibers, and skin elasticity
PRF for peri‐oral skin regeneration					
Hamid et al.^108^	*CT*	10 patients ♀ = 10/♂ = 0 Age: 18–33 years Smokers 0%	PRF((R)) PROCESS system technology i‐PRF 700 rpm 3 min 60 *g* No AC	Single session 0.5 mL PRF injected into each lip quadrant (total 2 mL)	*FU after 3 months* FACE‐Q assessment and ProFace® (data at week 0 week 12) Injection of PRF resulted in significant lip rejuvenation at 3‐month follow‐up. No volume change SS improvement from baseline (*p* = 0.04 and *p* = 0.02, respectively). Satisfaction with lip lines showed a numerical improvement with mean total scores for adverse effect scales related to the skin and lips reduced at 2 weeks post‐procedure (*p* = 0.03 and *p* = 0.13, respectively). Overall lip volume at 3‐month follow‐up was unchanged (*p* = 0.11) Final conclusion: The treatment was well tolerated with only minor adverse effects. A single session of i‐PRF+ injections resulted in significant lip rejuvenation at 3‐month follow‐up, shown by improved patient‐reported outcome measure. No significant change in lip volume was observed

Abbreviations: Study type: CCT, controlled clinical trial; CS, case series; non‐R, nonrandomized; RCT, randomized controlled trial; spl‐F, split‐face. Centrifuge data: *g*, *g*‐force; rpm, revolutions/rotations per minute. Outcome: AC, anticoagulant; ACD, acid citrate dextrose. APC preparation: CaCl, calcium chloride. Administration: HA, hyaluronic acid; ID, intradermal; MN, microneedling. Assessment: Results: NS, Non Statistically significant; SS, Statistically significant; FU, Follow Up.

Hamid et al.^108^ conducted a study to evaluate the efficacy of PRF for lip regeneration and augmentation in 10 healthy females. PRF was injected into the perioral area using a small gauge needle. Lip contour was assessed by blinded evaluators using standardized photographs. Patient satisfaction was assessed using a validated questionnaire. Lip rejuvenation was positive and still present 3 months after the procedure. No change in volume was observed after 3 months, which is important to note when comparing results to HA fillers that may last anywhere from 6 to 18 months. The lip augmentation was well tolerated, with minimal side effects.

### 12.3 Conclusion

**FIGURE 18:**
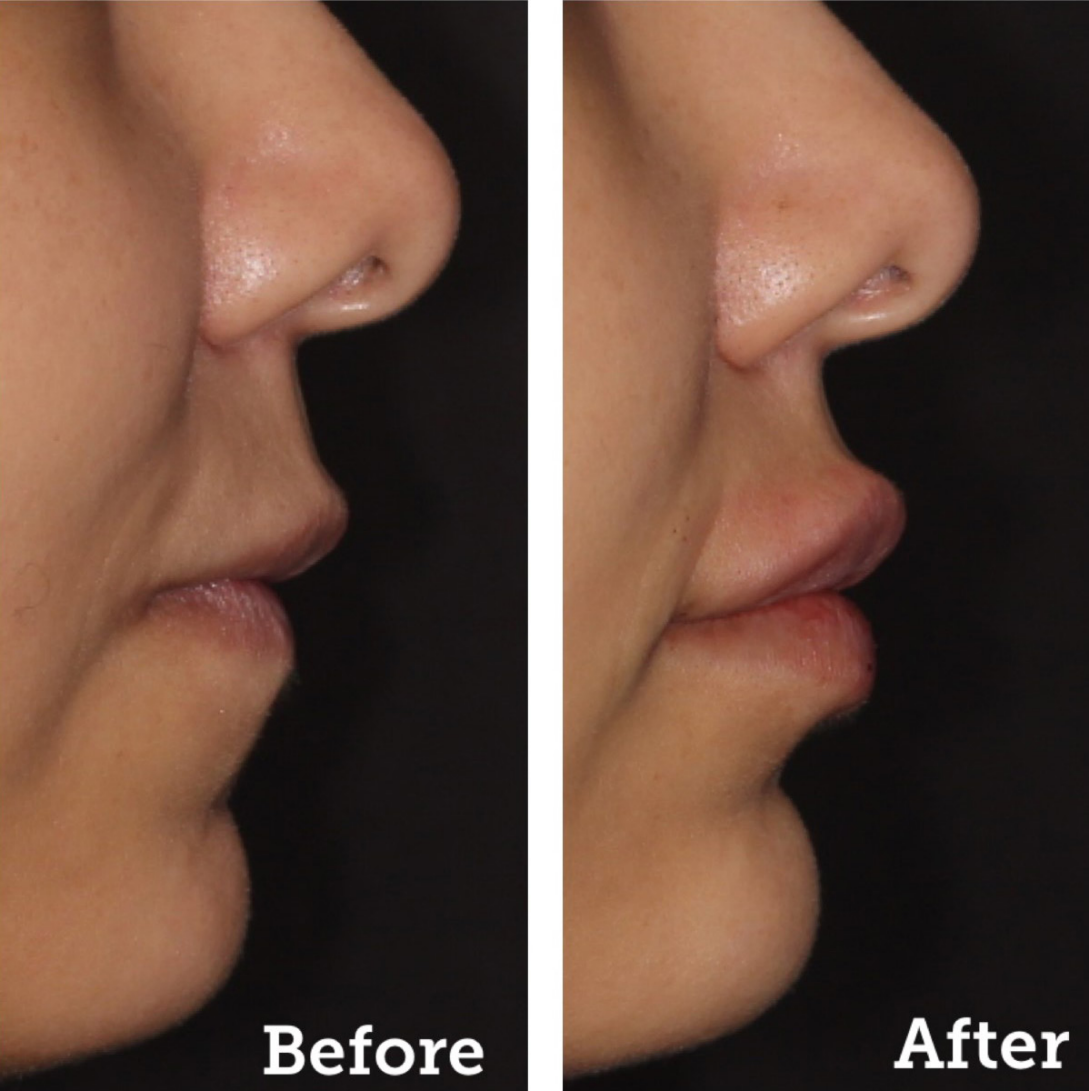
Before and after photos of lip rejuvenation. Note the improvements in fine line and wrinkles and color improvement following PRF use. Noteworthy, PRF is not utilized to volumize the lips similar to HA fillers commonly used.

Existing studies suggest that APCs are a safe and effective treatment option for lip rejuvenation not requiring large augmentations in esthetic medicine (Figure 18). However, more studies are needed to determine the optimal concentration and technique of platelet concentrate administration for this application and better elucidate their functional roles in lip augmentation.

A validated measurement system is needed with specific criteria such as lip volume increase, shape change as well as rejuvenation effect. Limitations of lip injections with APCs, as opposed to HA filler, are that although the rejuvenation effect is present after 3 months, the 3D volumizing effect does not last. Further studies are needed to elucidate these factors and study the safety and efficacy of combining APC with HA filler (QR Code 5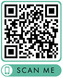).

### 12.4 Clinical guidelines for lip injections with APCs

Injections of liquid APCs into the lip may be done via

- needle (27 to 30 gauge) into each quadrant, in a retrograde fashion. Injection depth is above the orbicularis occuli muscle for vessel safety;
- or via by cannula (25 gauge), with only two entry points.

Between 0.2 to 0.5 mL is to be injected per quadrant.

## 13 APCs IN ANDROGENETIC ALOPECIA

### 13.1 Background

Androgenetic alopecia (AGA) is the leading cause of hair loss in both men and women. Within a culture that places a high premium on hair and links it to attractiveness, hair loss can result in detrimental psychological effects in some patients and can impact an individual's quality of life.^109^ Male (AGA) affects 58% of men by age 50.^110^

The term androgenetic alopecia (AGA), as used in this publication, is used synonymously with PHL (Patterned Hair Loss). MPHL refers Male Patterned Hair Loss, and FPHL to Female Patterned Hair Loss which are both equivalent to AGA. AGA in males occurs in a highly reproducible pattern, preferentially affecting the temples, vertex, and mid‐frontal scalp. In females, it occurs as central region thinning with a preserved frontal hairline.^111^ Currently, the Hamilton‐Norwood classification system for males and the Ludwig system for females are most used to describe patterns of hair loss.^112^ Managing androgenetic alopecia can enhance comprehensive patient care and lead to better patient outcomes.

While many patients choose not to undergo treatment there are approved treatments available. FDA approved Drugs such as topical minoxidil (for both sexes) and oral finasteride (for men)^113^ are widely employed to treat AGA, as are and low‐level 655‐nm laser devices. These treatments need to be continually used to be effective (every 6 months is recommended for APCs and 3 times per week for LLLT). Injectable APCs have become increasingly popular as a treatment option for (AGA). Other modalities include hormonal therapies, nutraceuticals, exosome treatments, and hair transplantation.

APCs have been researched as an alternative or additional treatment for AGA patients. They have been shown to induce the proliferation of the dermal papilla and increase the vascularization of the perifollicular tissue. They may also promote hair regrowth by accelerating the telogen‐to‐anagen transition, improving the appearance of hair loss.^114^ PRP and PRF has been shown to facilitate hair follicle regeneration by promoting human dermal papilla cell proliferation, migration, and trichogenic inductivity.^115^

RCTs were assessed to evaluate the effect of APC treatments on the main outcome parameters, hair density and hair thickness. Various methods are available for monitoring a response to treatment in a patient with hair loss as presented below.^116^ No one single method is ideal as a standalone measure in a clinical trial setting. Most studies use a few of these valuable tools for patient diagnosis and monitoring. Analytical methods such as a trichogram are optimal for a clinical trial, while global photography and patient questionnaire are of greater significance to the individual patient's satisfaction. Biopsy methods are viewed as invasive yet can be useful for assessing hair follicle morphology and immunohistology in a clinical trial setting.

**Table:** 

Assessing the response to treatment in androgenetic alopecia (AGA) Photography: Before‐and‐After Photos: for a visual comparison to evaluate any improvements or changes in hair growth. Trichoscopy: involves using a specialized magnifying device called a trichoscope/folliscope to examine the scalp and hair follicles in detail. This method can help identify signs of hair thinning, hair miniaturization, and the presence of vellus (fine, nonterminal) hairs. Hair Counts: involve counting the number of hairs in a specific area of the scalp before and after treatment. Hair Diameter Measurement: A micrometer or specialized tools can be used to measure the diameter of individual hair shafts. An increase in hair shaft diameter indicates a positive response to treatment. Pull Test involves gently tugging on a cluster of hairs to assess their fragility. Fewer hairs coming out during the test can indicate improved hair strength. Patient Self‐Assessment Questionnaire: Patients may be asked to assess their own hair growth and overall satisfaction with treatment using standardized questionnaires. Trichogram: This method involves taking photographs of the scalp and analyzing them using computer software to measure hair density, hair diameter, and T/V ratio (terminal/vellus). It provides objective data on treatment response. In some cases a Scalp Biopsy may be performed to examine the hair follicles under a microscope. Immunohistology can be performed. Global Physician Assessment (GPA)

### 13.2 Outcome

Numerous clinical trials have studiede growth of hair using PRP,^117^, ^118^, ^119^, ^120^, ^121^, ^122^, ^123^, ^124^, ^125^, ^126^, ^127^ showing a significant increase in the main outcome measure, the number of hairs per cm^2,^ after PRP injections compared to controls^117^, ^118^, ^119^, ^120^, ^121^, ^123^, ^126^, ^127^, ^128^ (Table 9).

**TABLE 9:** Use of APCs for androgenic alopecia.

Author	Study type	Subjects Gender Age % Smokers Condition	Centrifuge rpm/min *g* force anticoagulant	Treatment protocol and follow‐up	Outcome *Grading System result*
PRP for alopecia					
Cervelli et al.^117^	*NRCT* *Spl‐sc*	10 patients ♂ = 10 Age: 22–60 years AGA	Cascade‐Selphyl‐Esforax 1100 *g* 10 min Activator: Ca^2+^	Three treatment sessions, 1 month apart Intradermal PRP vs. placebo	FU 3 months after last treatment Hair Count: incr 18 hairs compared to baseline *p* < 0.0001 Trichoscan: Mean increase in total hair density of 27.7 *p* < 0.0001 Increase in number of basal keratinocytes *p* < 0.05 showed increase epidermal thickness *p* < 0.05 Final conclusion: PRP injections are safe and effective for AGA
Gentile et al.^118^	*RCT* *Spl‐sc*	23 patients ♂ = 23 (3 excluded) Age: 19–63 years	Cascade‐Selphyl‐Esforax system Single spin 1100 *g* 10 min AC: sodium citrate	Three treatment sessions, 1 month apart Month 0, month 1, month 2 Intradermal PRP vs. placebo Spl‐*sc*	FU 12 months after last treatment Hair Counts: mean increase—33.6 hairs treated area (*p* < 0.0001) Trichogram: hair density, mean increase total hair density of 45.9/cm^2^ vs. baseline values (*p* < 0.0001) Final conclusion: Significant increase in hair count and terminal hair density in PRP compared to placebo
Alves and Grimalt^119^	*RCT*	25 patients ♀ = 13/♂ = 12 Age: 21–62 years	Single spin method 460 *g* 8 min AC: sodium citrate Activator: CaCl	L‐PRP R‐saline Three treatments PRP 1 month apart	FU 6 months after Rx SS differences compared with baseline (*p* < 0.05). anagen hairs (67.6 ± 13.1), telogen hairs (32.4 ± 13.1), hair density (179.9 ± 62.7), and terminal hair density (165.8 ± 56.8) PRP was also found to increase hair density when comparing with the control side Final conclusion: PRP showed a positive effect on AGA
Anitua et al.^120^	*NRCT*	19 patients ♀ = 7/♂ = 12 Age: 32–60 years	Single spin method 580 rpm 8 min AC: sodium citrate PRGF activator	Five sessions total Four sessions 1 month apart One session after 7 months	FU after 1 year Trichoscope: density, diameter, T/V ratio ratios significantly improved over baseline: Density (*p* < 0.05) Photograph Improvement seen. Scalp Biopsy Epidermal thickness improved (*p* < 0.05 for most) Final conclusion: Study supports positive therapeutic effect of PRP on hair follicle regeneration
Tawfik et al.^121^	*RCT* *Spl‐sc*	30 patients ♀ = 30 Age: 30–45 years AGA	Centrifuge model 80‐2A Double spin 1200 *g*—15 min/200 *g*—10 min AC: sodium citrate Activator: calcium gluconate	Four treatments 1 week apart Intradermal PRP vs. saline	FU 6 months after last treatment Hair Pull Test: significantly negative after treatment in 83% patients PRP treated areas (vs. basline): hair density incr (*p* < 0.005); hair thickness incr (*p* < 0.005) High overall pt. satisfaction in PRP group Final conclusion: PRP injections can be regarded as an alternative for the treatment of female pattern hair loss with minimal morbidity and a low cost‐to‐benefit ratio
Sasaki et al.^122^	*RCT* *Spl‐sc*	8 patients ♀ = 4/♂ = 4 Age: 34–65 years	Single spin 10 min 2950 rpm Batch A (low platelet) L‐PRP Batch B (high platelet): H‐PRP PRP Sodium citrate AC	Two session 3 months apart Group A: L‐PRP vs. N/S Group B: H‐PRP vs. N/S	FU after 6 months Trichoscan: both higher (H‐PRP) and lower (L‐PRP) numbers of platelets resulted in numerical increases in hair densities, follicle diameters, and terminal hair densities, as well as absolute number and percentage changes over their baseline measurements Minimal difference in density between the groups Final conclusion: Higher numbers of platelets *may* have a greater effect than lower numbers of platelets in regard to hair density, follicle diameter, and terminal hair density. Minimal effects on vellus hair densities at the 6‐month evaluation point
Rodrigues et al.^123^	*CCT*	26 patients ♂ = 26 Age: 18–50 years	Double spin with PLT activator PLT count: 1200 × 10^6^/μL	Four injections (every 15 days) weeks Group 1 PRP (15) Group 2 saline (11)	FU up 3 months after Rx SS: increase in hair count (*p* = 0.0016) SS: increase in hair density (*p* = 0.012) and percentage of anagen hairs (*p* = 0.007) in the PRP group vs. in the control group NS: findings vs. control: terminal/vellus hair ratio (Trichoscan); Individual PDGF, EGF, VEGF correlation with outcomes Final conclusion: PRP significantly increased hair growth. No correlation with platelet counts or quantification of the growth factors in PRP
Mapar et al.^124^	*RCT*	17 patients ♂ = 17 Age: 25–40 years	Double spin method using Tubex PRP tube. Activator: Calcium gluconate 3000 rрm 6 min 3300 rpm 3 min	PRP vs. normal saline intradermal into diff squares Two treatments 1 month interval	FU 6 months after last treatment Magnifying Glass NO change in hair count NO change in hair mass index NS findings vs. baseline: Increased Terminal hair count, vellus hair count Final conclusion: PRP did not improve hair growth
Puig et al.^125^	*RCT*	26 patients ♀ = 26 Age: 24–45 years AGA	Angel PRP system (Cytomedix) 3000 rpm—6 min 3300 rpm—3 min AC: sodium citrate Activator: calcium gluconate	Single subcutaneous injection in centre scalp Group 1—PRP Group 2—Saline	FU 26 weeks Hair Counts: No change Trichoscope: No change in vellus hair Questionnaire: Treatment group vs. control group reported substantial improvement in hair loss, rate of hair loss, hair thick ness, and ease of management/styling hair. Final conclusion: No statistically significant difference between the two groups. NS findings vs. control: increased hair mass index and hair density.13.3% reported SS decreased hair loss
Shapiro et al.^126^	*RCT*	17 patients ♂ = 17 Age: 18–58 years	1500 *g* 5 min	GrA vs. B placebo or PRP Three sessions monthly F/U 3 months after last treatment	FU 3 months after last treatment Significant increase in hair density and hair thickness in PRP compared to baseline, 20 hairs/cm^2^ (*p* < 0.05) No significant increase compared to placebo PRP *may* have benefit in increasing hair density Final conclusion: No significant difference in hair density change between the two groups
Ozcan et al.^127^	*RCT*	62 patients ♂ = 62 Age: 20–47 years	Single spin 8 min 2800 *g*	Four sessions: Three sessions @ 2‐week intervals then last session 1 month Group A MN Group B Intradermal injections	Hair Pull Test: negative after treatment (*p* < 0.05) Hair Counts^118^, ^128^, ^129^, ^130^, ^131^, ^132^, ^133^, ^134^, ^135^: SS difference in both groups vs. baseline (*p* < 0.05) SS difference density in both groups vs. baseline (*p* < 0.05) SS difference: anagen hair, telogen hair and hair length in the dermapen vs. injection Final conclusion: MN was superior to the injection technique in terms of anagen, telogen and average hair length
Asim et al.^128^	*RCT*	72 patients Group A ♀: 6 ♂: 30 Group B ♀: 6 ♂: 30 Age: 20–60 years AGA		12 weeks A PRP group‐monthly injections × 3 B Minoxidil 5%: 1 mL topical daily Month 0, month 1, month 2	FU 12 weeks after last treatment A Negative HP PRP; 91.7% B Negative HP Minoxidil: 69.4% >12 weeks statistically Significant difference between negative hair pull A vs. B (*p* = 0.017) Final conclusion: PRP therapy demonstrates a higher efficacy compared to minoxidil for treating AGA
Verma et al.^129^	*RCT*	30 patients ♂ = 30 Age: 22–30 years	Double‐spin technique 1500 rpm—5 min/2500 rpm—15 min Activator: calcium gluconate	Group A: PRP therapy (intradermal injections monthly × 4) Group B: minoxidil therapy 5% 1 mL bd	FU 6 months after last treatment Photography Group A (PRP) better outcome than Group B (minoxidil). Hair Counts: Group A better than Group B Pull Test Group A better than Group B Questionnaire: Group A better than Group B Final conclusion: PRP therapy and minoxidil therapy found that patients treated with PRP had a significantly higher satisfaction score and more negative hair pull tests than patients treated with minoxidil Platelet counts baseline: 3.07 ± 0.5 lac/mm, three while platelet count in final PRP: 12.4 ± 1.7 lac/mm, and Final conclusion: Patients with a higher platelet count in PRP had a much better clinical improvement compared to patients with a low platelet count in PRP
Balasundarum et al.^131^	*RCT*	64 patients ♂ = 64 Age: 20–50 years	Double spin 10 min 400 *g* Then 10 min 900 *g*	Minoxidil arm: Topical minoxidil 5% at 1 mL twice day for 6 months PRP arm: Three sessions 1 month apart	FU 24 weeks Both PRP and topical minoxidil are effective treatment for AGA PRP is not superior to minoxidil in treating moderate grades of AGA Final conclusion: No significant difference between groups in propensity to increase total hair count, terminal hair count and density. Minoxidil better tolerated than PRP (less pain)
Singh et al.^132^	*RCT*	80 patients ♂: 80 Age: 20–60 years AGA	Remi Model R8M 2200 rpm 12 min AC: sodium citrate Activator: calcium gluconate	Four groups. PRP with Minoxidil PRP alone Minoxidil alone NS Injections given 3× 1 month apart	FU after 5 months PRP with Minoxidil > PRP alone > Minoxidil alone > NS Hair density: highly SS increase 3 months of treatment group I (*p* < 0.001) Improvement in hair density was seen in three groups (minoxidil group, PRP with minoxidil group, and PRP group) Satisfactiom PRP with Minoxidil > PRP alone > Minoxidil alone > NS Final conclusion: PRP with topical minoxidil was the most effective treatment modality while PRP alone and topical minoxidil alone were more effective than placebo
Pachar et al.^133^	*CCT* *Spl‐sc*	50 patients ♂ = 50 Age: 18–54 years AGA	Double spin 1200 rpm 8 min 2400 rpm 4 min	PRP intradermal to LHS scalp (monthly × 6) Minoxidil 5%: both sides (daily)	FU after 6 months Hair density: minoxidil 5% only side at the first visit was 93.97 ± 4.0 and the last visit was 104.77 ± 4.97, and that Hair density PRP + minoxidil 5% side at the first and last visits was 93.97 ± 4.0 and 113 ± 7.66, respectively. Final conclusion: Both sides showed a statistically significant increase The mean hair density after the last visit showed a significant difference in the PRP + minoxidil 5% side
Wei et al.^134^	*CCT*	30 patients ♂ = 30 Age: 21–49 years AGA	Fresenius COM.TEC, automatic blood cell separator	*Group A (PRP* + *M)*: PRP + topical 5% minoxidil therapy. *Group B (PRP* + *P)*: PRP + topical placebo therapy. PRP—3 treatments 1 month apart	FU 4 weeks after last Rx Trichoscope: density diameter ratios: hair density/quantity of all patients SS increase after treatment (*p* < 0.05) Average hair density increased in group A was higher than that in group B, but no significant difference (*p* = 0.26 > 0.05). Final conclusion: PRP injection combined with topical 5% minoxidil therapy is safe and effective
Pakhomova et al.^130^	*RCT*	69 patients ♂ = 69 Age: 18–53 years AGA	Double spin 5 min—570 *g*/1200 *g*—10 min AC used Activator CaCl	Group 1 PRP Group 2 Complex therapy (PRP + Minoxidil) Group 3 Minoxidil	Group 2 Hair density increased by: 1.74 times *p* = 0.0347 Group 2 Hair diameter increased by 14.3 times *p* = 0.00001 Group 2 decrease proportion of telogen hair 9.3 times *p* = 0.00003 Proliferative activity (β‐catenin, CD34, Ki67, Dkk‐1) PRP increased the proliferative activity of HF cells and improves hair morphology in patients with AGA Final conclusion: Complex therapy (PRP with minoxidil) is more effective than minoxidil monotherapy (*p* < 0.0001) and PRP monotherapy. PRP can be considered as a treatment option for AGA
Gentile et al.^136^	*RCT*	90 patients ♂ 63 ♂ 27 Age: 18–53 years	Cascade‐Selphyl‐Esforax system 1100 rpm 10 min 1200 rpm 10 min Activate vs. Non Activated	Group A: AA‐PRP (activated) Group B: A‐PRP (non activated)	FU 58 weeks after last Rx Hair density measurements AA PRP 23 ± 3 and A PRP 13 ± 3 hairs/cm (2) Nonactivated PRP was found to have greater increase in hair count and total hair density (31% ± 2% vs. 19% ± 3%, *p* = 0.0029) than patients treated with activated‐PRP Final conclusion: PRP does not require activation before injection PRP increases in the proliferative activity of HF cells and improves hair morphology inpatients with AGA
Lee et al.^135^	*RCT*	40 patients ♀ = 40 Age: 20–60 years AGA	SmartPrep2 APC System AC: sodium citrate	Group 1 PRP then weekly PRDN × 12 (polydeoxyribonucleotide) Group 2PRDN weekly × 12 only	FU 3 months Combined therapy with PRP and PDRN induces greater improvement in hair thickness than treatment with PDRN therapy alone (*p* = 0.031), but not in hair counts (*p* > 0.05). Final conclusion: Intra‐perifollicular injections of autologous PRP and/or PDRN generate improvements in hair thickness and density in FPHL patients
PRF for alopecia					
Arora et al. 2019^137^	*CS*	10 patients ♂ = 10 Age: 35–40 years	Duo centrifuge 700 rpm 5 min	Intradermal injection 4 Rx 15 days apart	Fu after 4 months Individual cases assessed and were satisfied Final conclusion: All cases showed improvement in hair growth with liquid PRF
Schiavone et al.^138^	*RCT*	168 patients ♀ = 66/♂ = 102 Age: 25–60 years	i‐PRF – single spin 5 min 1500 rpm ACD anticoagulant used	2 groups (TG 126 CG 28) PRF injections month 0, month 3	FU 6 months after Rx TG better scores than control group SS findings vs. control: GPA scale, Jaeschkle's scale GPA: SS improvement in the GPA across all ages and genders (*p* < 0.001) and genders (*p* < 0.001). SS appeared to increase in more severe grades of AGA Final conclusion: PRF showed clinical efficacy in AGA
Bhoite et al.^139^	*CS*	15 patients ♀ = 3/♂ = 12 Age: 30–50 years Smokers 0%	REMI‐R8C centrifuge machine 700 *g* 6 min	PRF and microneedling Four sittings—2 weeks apart background therapy of minoxidil, finasteride and multivitamin supplements	A significant improvement in hair growth was observed clinically with positive score of 7.42 on the patient satisfaction scale and visible changes were noticed on clinical photographs and dermoscopy Final conclusion: PRF is safe, easy, time and cost‐effective adjuvant modality for managing androgenetic alopecia with some theoretical advantages over PRP
Sclafani et al.^140^	*CCT*	15 patients ♀ = 6/♂ = 9 Age: 27–51 years	Single spin: 1100 *g* 6 min	PRF intradermal scalp injections Three sessions 1 month apart	FU after 6 months SS findings vs. baseline: increased hair density index at 2 and 3 months NS findings vs. baseline: improved hair density index at 6 months Other findings: 25% improvement in hair density index at 2 months predicts the response retained at 6 months Final conclusion: PRF is a valuable treatment for managing androgenetic alopecia particularly in mild cases
Mahapatraet al.^141^	*CS*	10 patients ♂ = 10 Age: 18–50 years Norwood 4 to 6	DiponEd BioIntelligence LLP protocol 3000 rpm 10 min No AC	PRF: Split scalp Injection month 0, month 1, month 3 After hair transplantation	FU 1, 2, 6 months SS >1 month (25.60 ± 3.38, *p* < 0.001), 2 months (21.50 ± 5.09, 0.002), and 6 months (26.00 ± 4.63, *p* = 0.005) Final conclusion: PRF treatment showed beneficial effect on hair follicle numbers when done with hair transplantation

Abbreviations: Study type: CCT, controlled clinical trial; CS, case series; non‐R, nonrandomized; RCT, randomized controlled trial; spl‐s, split‐scalp. Centrifuge data: *g*, *g*‐force; rpm, revolutions/rotations per minute. Outcome: APC preparation: AC, anticoagulant; ACD, acid citrate dextrose; CaCl, calcium chloride. Administration: Condition: AGA, Androgenetic Alopecia. Administration: HA, hyaluronic acid; ID, intradermal; MN, microneedling.

An important factor in the effectiveness of PRP is the number of platelets. Studies have shown that a higher concentration of platelets have a greater effect than lower ones in terms of hair density, follicle diameter, and terminal hair density.^122^, ^129^ Rodrigues et al.^123^ did not find a correlation between growth factors measure and effect, and suggested that this may be attributed to the factors that were selected to be measured. Gentile et al.^136^ found no difference between activated and nonactivated PRP in their study and concluded that PRP does not have to be activated for AGA treatment.

When comparing application methods, Ozcan et al.^127^ found microneedling to be superior to the injection technique in terms of anagen, telogen and average hair length. Interestingly, Phakomova et al.^130^ assessed the proliferative activity of the hair follicle cells by measuring antibodies (β‐catenin, CD34, Ki67, and to Dkk‐1). It was concluded that PRP increased the proliferative activity of HF cells and improves hair morphology in patients with AGA.^130^ Cervelli et al.^117^ showed an increase in the number of basal keratinocytes and improved epidermal thickness.

Of some concern is that some studies reported ineffectiveness of PRP in AGA treatment.^124^, ^125^, ^126^ Possible explanations may be low platelet concentration, low volume of PRP injected, and inadequate frequency of treatment. Furthermore, genetics of individuals seems to be a key contributing factor to hair loss in general and its response to various treatments.

Studies comparing PRP to Minoxidil therapy, or as an adjuvant to Minoxidil showed that PRP combined with topical minoxidil was the most effective treatment modality,^128^, ^130^, ^131^, ^132^, ^133^, ^134^ while PRP alone and topical minoxidil alone were more effective than placebo. PRP monotherapy was more effective than minoxidil monotherapy in most studies,^129^ although Balasundarum et al.^131^ found them to be equally effective. Minoxidil was shown to be better tolerated than PRP,^131^ due to the pain incurred during PRP injections. In conclusion, investigators found that topical 5% minoxidil in combination with intradermal PRP has higher efficacy than topical 5% minoxidil alone in AGA. It is a useful therapy in poor responders to conventional therapy.

Lastly, Lee et al.^135^ compared PRP scalp injections to polydeoxyribonucleotide (PDRN) injections and concluded that, intra‐perifollicular injections of autologous PRP and/or PDRN both generate improvements in hair thickness and density in FPHL patients, however combining the two induces greater improvement in hair thickness.

While PRP has been the main APC in use for hair regeneration in AGA over the past decade, data regarding the efficacy of PRF in treating AGA is limited. A PubMed search was conducted with keywords PRF and AGA and no controlled studies were available to date (Oct 2023). A few case studies have been performed which show that PRF may be promising in the management of AGA proposed that PRF may have a greater potential to regenerate hair than PRP based on based on a study by Masuki et al.,^142^ who concluded that PRF contains a longer release of growth factors when compared to PRP,^143^ which not only functions as a scaffold but also a reservoir of growth factors. However, no RCTs are available for PRF to date, and no comparative studies versus PRP exist. Nevertheless studies assessing the efficacy of PRF demonstrated great potential regarding its clinical efficacy in AGA.^137^, ^138^, ^139^, ^140^

Schiavone et al,^138^ Bhoite et al.,^139^ as well as Arora and Shukla used injectable‐PRF to produce positive clinical results in patients with AGA. They observed an increase in hair density with statistical significance. Interestingly, patients with a greater degree of disease severity at baseline tended to achieve a larger improvement after treatment. Monitoring in these studies was done with clinical photographs and dermoscopic evaluation before each session, and a standard assessment questionnaire was given at the end of the study. A significant improvement in hair growth was also observed clinically with a positive score on the patient satisfaction scale. It was concluded that liquid‐PRF is a safe, easy, time and cost‐effective modality for managing androgenetic alopecia.

A split‐scalp study by Mahapatra et al.^141^ showed that using liquid‐PRF during hair transplantation was beneficial, and produced a greater retention of follicles, with a clear statistical difference after 6 months (26.00 ± 4.63, *p* = 0.005**).

### 13.3 Conclusion

Currently, the evidence to support the clinical efficacy of PRP in AGA loss is controversial.^144^ The number of clinical trials in this area has increased substantially over the years, however the studies are highly variable. Preparations, protocols, and treatment intervals vary, and many patients are not assessed for an adequate length of time. Improved and standardized study designs, including larger samples, quantitative measurements of effect, and longer follow‐up periods, are needed to optimize the use of APCs for treating AGA. The optimal number of PRP treatments, interval between treatment and the amount of PRP injected per treatment still need to be assessed. For best results it is advisable to apply a complex combined therapy protocol as early as possible. Further research should also evaluate protocol differences between male^117^, ^118^, ^136^, ^145^, ^146^ versus female outcomes.^147^

PRF appears to be promising as a safe and effective treatment of AGA, and as an adjuvant to hair transplant surgery. RCTs evaluating the effect of PRF in hair regeneration are needed. Protocols and administration methods should be standardized for PRF studies to avoid the pitfalls of PRP studies in alopecia. Additionally, it would be interesting to know how long the effect of PRF lasts after termination of therapy. A split scalp study comparing PRF and PRP would also be valuable to compare the efficacy and safety of both. Future longitudinal studies would be very useful. Furthermore, some studies have begun to compare PRP to hair follicle stem cells (HFSCs) as well as compared to other common procedures such as Minoxidil and Finasteride.^148^, ^149^

A task force assigned by the Indian Association of Dermatologists were asked to provide a framework for clinicians on the use of PRP.^150^ A total of 30 articles were evaluated.

The recommendations on the preparation of PRP for AGA treatment, resulting from the review are as follows.

- Use a manual double‐spin method for preparation of PRP for AGA.
- Perform a minimum of 3–5 sessions of PRP.
- 1 month interval between sessions.
- Recommended dose is; 5–7 mL PRP, using 0.05 to 0.1 mL/cm^2^.
- Activation of PRP is not required when it is used for AGA.
- About 1 to 1.5 million platelets per uL is the recommended concentration of platelets in PRP.

The task force also recommended further studying the use of PRF for AGA treatment.

### 13.4 Clinical guidelines

**FIGURE 19:**
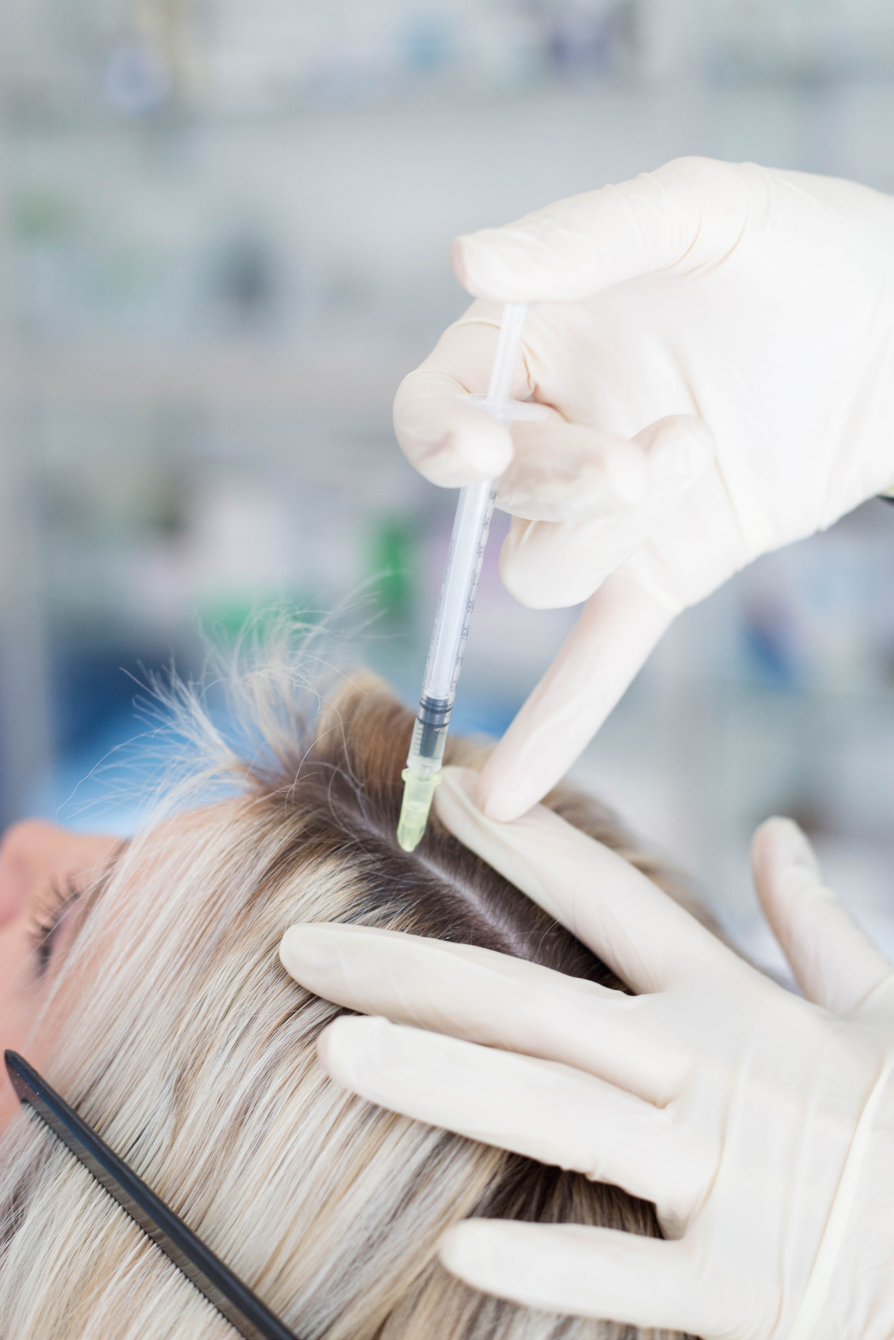
Clinical photo demonstrating the point injection technique into the scalp. Reprinted with permission from Davies/Miron.^8^

**FIGURE 20:**
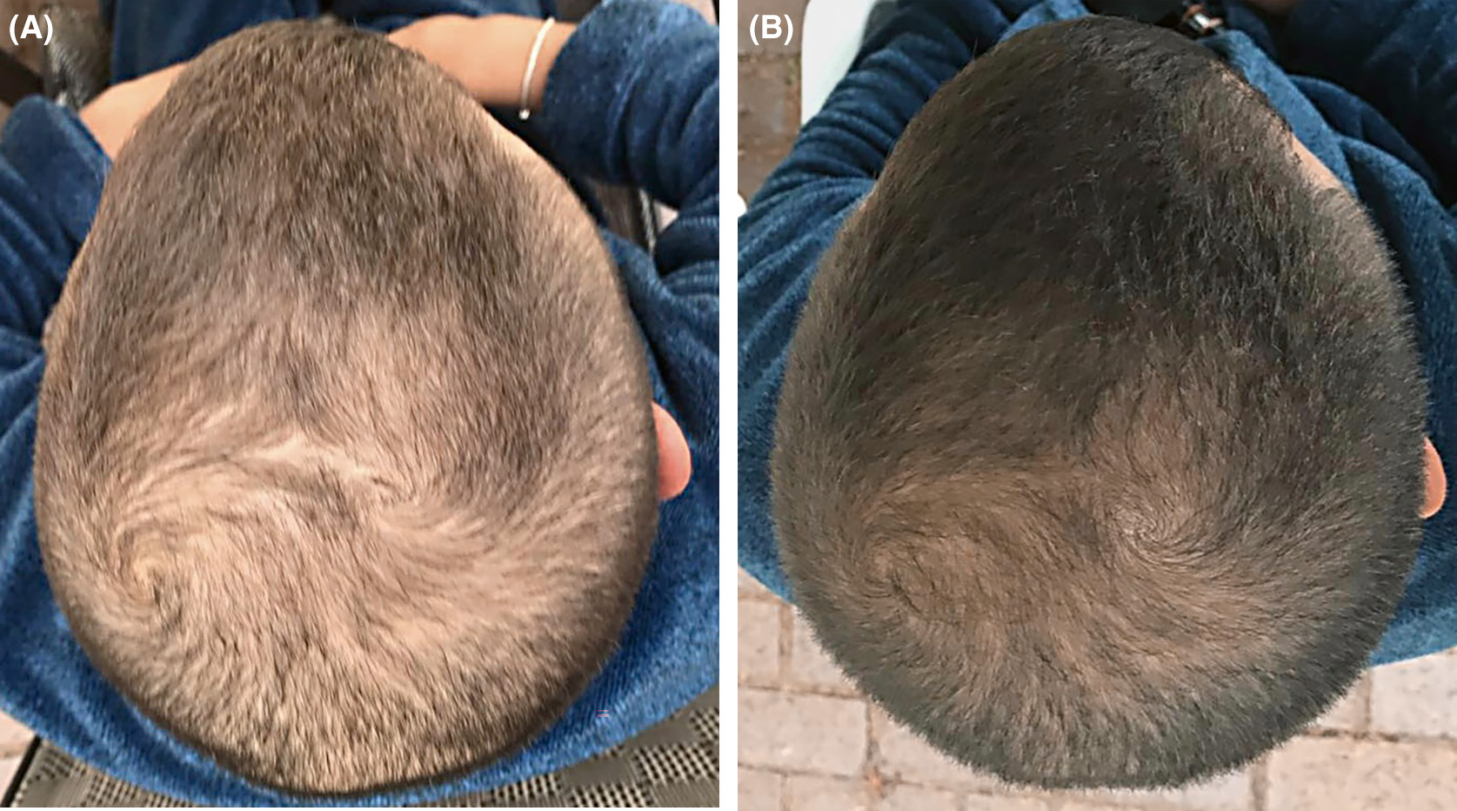
Results (A) before and (B) after three sessions of PRF injections. Reprinted with permission from Davies/Miron.^8^

**FIGURE 21:**
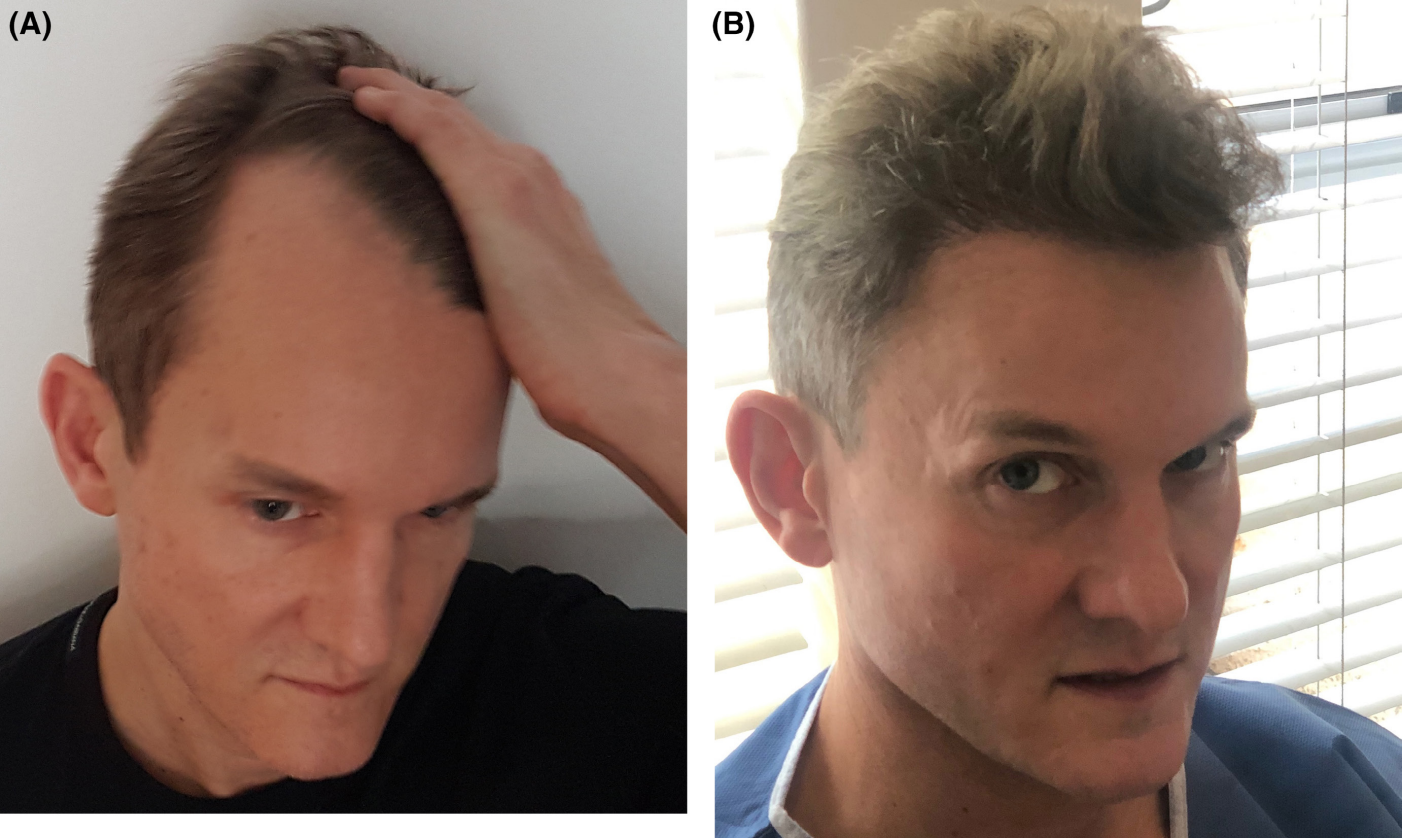
(A) Male patient demonstrating noticeable hair loss. Following follicular unit extraction (FUE) with PRF pre‐treatment 7 days prior and follicles soaked in PRF during surgery, (B) Notice the substantial early hair regrowth (month 6) and pleasing esthetic outcome. Reprinted with permission from Davies/Miron.^8^

Typically injections are done using 30G × 4 mm needles since the therapy in general has been associated with pain upon injections. Therefore, high‐quality needles, proper anesthesia with topically applied pharmaceutical grade numbing cream are highly recommended. The hair is then parted to allow for the rapid injection of APCs into the scalp. PRF is typically favored as the “burning” sensation is minimized since it has been postulated that it is caused from the use of anti‐coagulants and various activators commonly utilized in PRP. Figure 19 demonstrated a point injection technique into the scalp commonly utilized (QR Code 6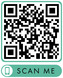). Figure 20 demonstrated before and after three sessions utilizing PRF injection techniques in a male with AGA. More recently, clinicians have also utilized APCs during hair transplants, giving improved graft retention/survival (Figure 21).

**Table:** 

Protocol: Map out areas of hair loss Inject into scalp in these area point by point at a 90 degree angle between 2 to 4 mm depth (full bevel of needle) Injections 1 cm apart Treatment phase: 1 session every 4 to 6 weeks × 3 sessions in total per annum Evaluation after one year Maintenance phase: 1 session every 6 months

Tips: Ask the patient to shampoo and detangle before session. The patient must apply No product to the hair such as wax, gel, hair spray. Take excellent before and after photos. Comb through the area before injection. Mark out the area to be injected Optimal positioning. Make sure the scalp can be reached from all angles. Prepare everything before withdrawing blood, as timing is limited in case of PRF use.

## 14 APCs FOR USE AS A VOLUMIZING AGENT

### 14.1 Background

One of the reported disadvantages of PRP/PRF is its rather liquid consistency in nature and faster‐than‐ideal resorption rates. Liquid‐PRF is ideal for minimizing fine lines and wrinkles but is not effective at replenishing lost volume with age. Therefore, the Bio‐Filler was developed in order to maintain more volume over time, all while supplying the body with growth factors capable of stimulating collagen regeneration and building collagen over time.

To make use of this innovative technology, a certain heating apparatus known as Bio‐HEAT is necessary. The Alb‐PRF is generated by obtaining peripheral blood by the use of 9–10 mL tubes, without the inclusion of any other substances. Peripheral blood is initially drawn and, preferably, centrifuged for 8 min (between 700 and 2000 RCF) in a horizontal centrifuge. Following processing, blood layers may be seen to separate into plasma and the leftover decanted red blood cells.

**FIGURE 22:**
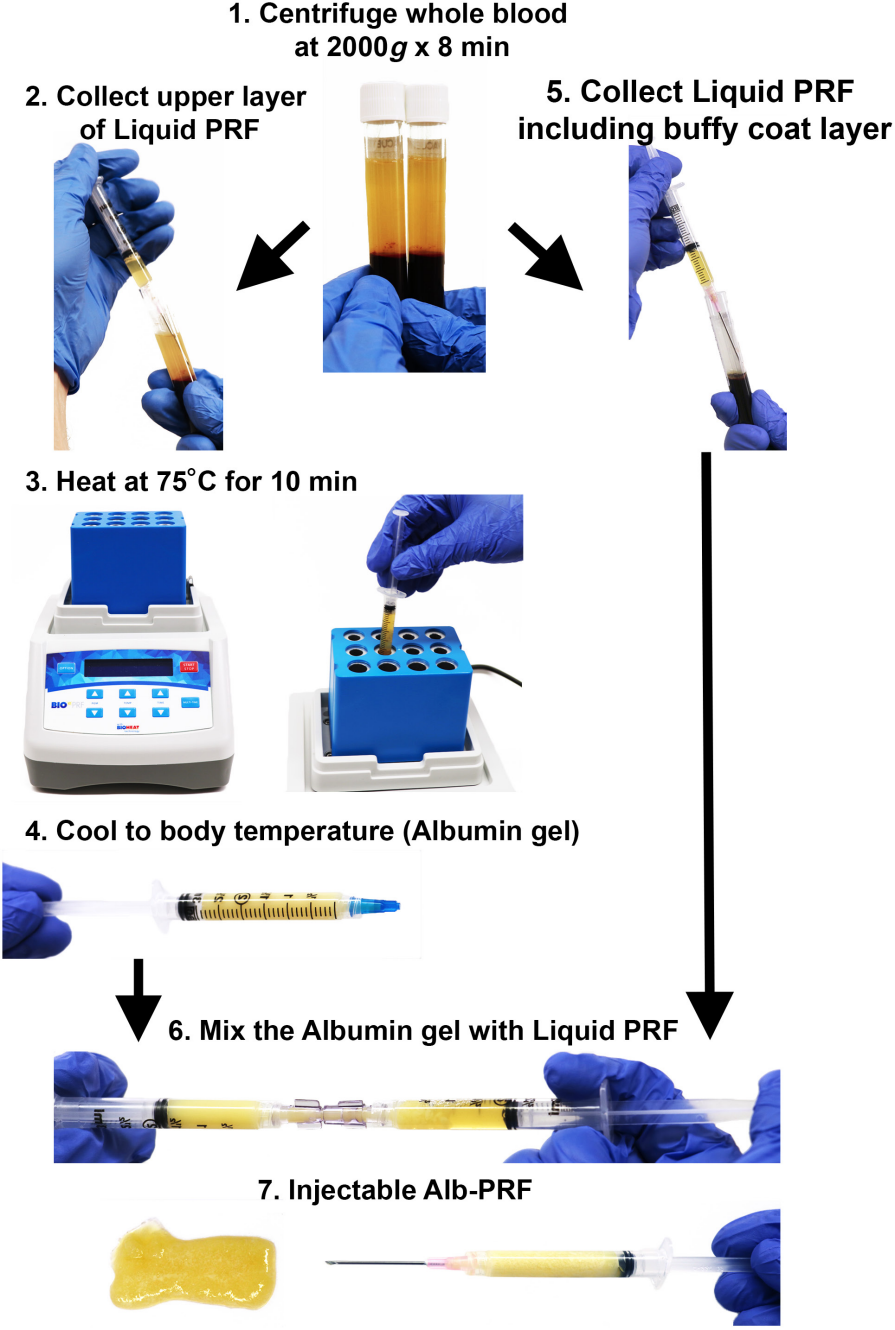
e‐PRF preparation protocol. (1) Whole blood was centrifuged at 2000 *g* for 8 min. The upper layer (yellow layer) shows the liquid plasma layer. (2) The most upper layer of platelet‐poor plasma (PPP) was collected in a syringe. (3) The collected PPP was heated in a heat block device at 75°C for 10 min and thereafter (4) cooled to room temperature for approximately 10 min. An injectable albumin gel was then prepared. (5) The liquid platelet‐rich layer (liquid‐PRF), including the buffy coat layer with accumulated platelets and leukocytes, was collected in a separate syringe. (6) The albumin gel and native liquid PRF were then thoroughly mixed by utilizing a female–female luer lock connector. (7) Injectable e‐PRF in final ready form. Reprinted with permission from Fujioka‐Kobayashi et al.^17^

Using a syringe, 2–4 mL of the first section of plasma (platelet‐poor plasma) are then collected (Figure 22), In order to reduce clotting, the remaining blood components—buffy coat, liquid PRF, and red blood cells—are put in a chilling apparatus. To create the albumin gel, the PPP‐filled syringes are thereafter placed into a heating apparatus for human serum albumin denaturation plasma. After 10 min at an operating temperature of 75°C, the syringes were then removed and cooled in the cooling unit to room temperature.^151^ Subsequently, a female–female luer lock connection is used to move back and forth between syringes to combine the albumin gel and the liquid PRF. (QR Code 7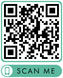). To ensure sufficient mixing, transfer this between the syringes about ten times back and forth. After that, the Alb‐PRF (also known as Bio‐Filler in the area of face esthetics) may be used as an injectable autologous concentration of denatured albumin, growth factors, and cells. This novel formulation is more useful for filling larger voids while retaining the regenerative properties of PRF (e.g., cheek‐bones, temple area, tear trough area, and nasolabial folds).^16^

### 14.2 Outcome

A case series by Doghaim et al.,^152^ enrolling 52 females with tear trough hollowing and rhytides, evaluated 34 females who had wrinkle injections and 18 females who had had tear trough injections using plasma gel (bio filler). Both groups showed an immediate significant clinical improvement after bio‐filler injection. This was measured objectively by the Wrinkle Severity Rating Scale in group A and Tear Trough Rating Scale and was confirmed to be significant and maintained until the end of the follow‐up period of 3 months. A more recent study by Mohammed Gabera et al.^153^ also showed that tear trough and crow's feet were significantly decreased among the studied patients at post‐plasma‐gel injection in comparison with pre‐plasma‐gel injection (*p* < 0.001). Both studies concluded that PPP gel injection seems to be a cost‐effective, safe, well‐tolerated, and minimally invasive technique producing significant esthetic correction of facial wrinkles and tear trough deformity (Table 10).

**TABLE 10:** APC use as a Biofiller.

Author	Study type	Subjects Gender Age % Smokers	Centrifuge rpm/min *g* force Anticoagulant	Treatment protocol and follow‐up	Outcome *Grading System*
Doghaim et al.^152^	*CCT*	52 patients ♀ = 52 Age: 40–55 years TTD	PRP double spin 320 *g*, 10 min 1000 *g* rpm, 5 min AC citrate dextrose Activator: calcium gluconate PPP only heated 60–100°C for 1 min, then cooled	Group A (34)—facial wrinkles Two sessions of deep intradermal PPP gel—2 weeks interval Group B (14)‐tear trough deformity Two sessions of deep intradermal PPP gel—2 weeks interval	FU 3 months after last treatment Statistically significant improvement in the facial wrinkles: WSRS decreased baseline 3.18 ± 0.81 before treatment to reach 1.65 ± 0.61 3 months 9 *p* value less than 0.050. SS clinical improvement: TTRS decreased from baseline: 15.67 ± 1.63 before to 3 months: 7.0 ± 1.10 (*p* < 0.05) Final conclusion: Autologous platelet poor plasma gel injection seems to be a cost effective, safe, well‐tolerated, and minimally invasive technique producing significant esthetic correction of facial wrinkles
Mohammed et al.^153^	*CCT*	200 patients ♀ = 200 Age: 40–65 years Facial winkles TTD	PRP Upper layer: 5 min 1000 *g* PPP heated at 15 min at 89°C	Group A (150)—satisfied One session of deep intradermal PPP gel Group B (50)—unsatisfied One session of deep intradermal PPP gel	FU after 2 weeks WSRS and TTRS after treatment: TTRS SS decreased among all patients. WSRS SS decreased (*p* < 0.05) Final conclusion: PPP gel injection seems to be a cost‐effective, safe, well‐tolerated, and minimally invasive technique producing significant esthetic correction of facial wrinkles and tear trough deformity

Abbreviations: Study type: CCT, controlled clinical trial; CS, case series; non‐R, nonrandomized; RCT, randomized controlled trial; spl‐s, split‐scalp. Centrifuge data: *g*, *g*‐force; rpm, revolutions/rotations per minute. Outcome: APC preparation: AC, Anticoagulant; ACD, acid citrate dextrose; CaCl, calcium chloride; PPP, Platelet Poor Plasma. Administration: HA, hyaluronic acid; ID, intradermal; MN, microneedling. Assessment, PRDN: TTD, tear trough deformity; TTRS, Tear Trough Rating Scale; WSRS, Wrinkle Severity Rating Scale. Findings: NS, nonstatistically significant finding; SS, statistically significant finding.

### 14.3 Conclusions

**FIGURE 23:**
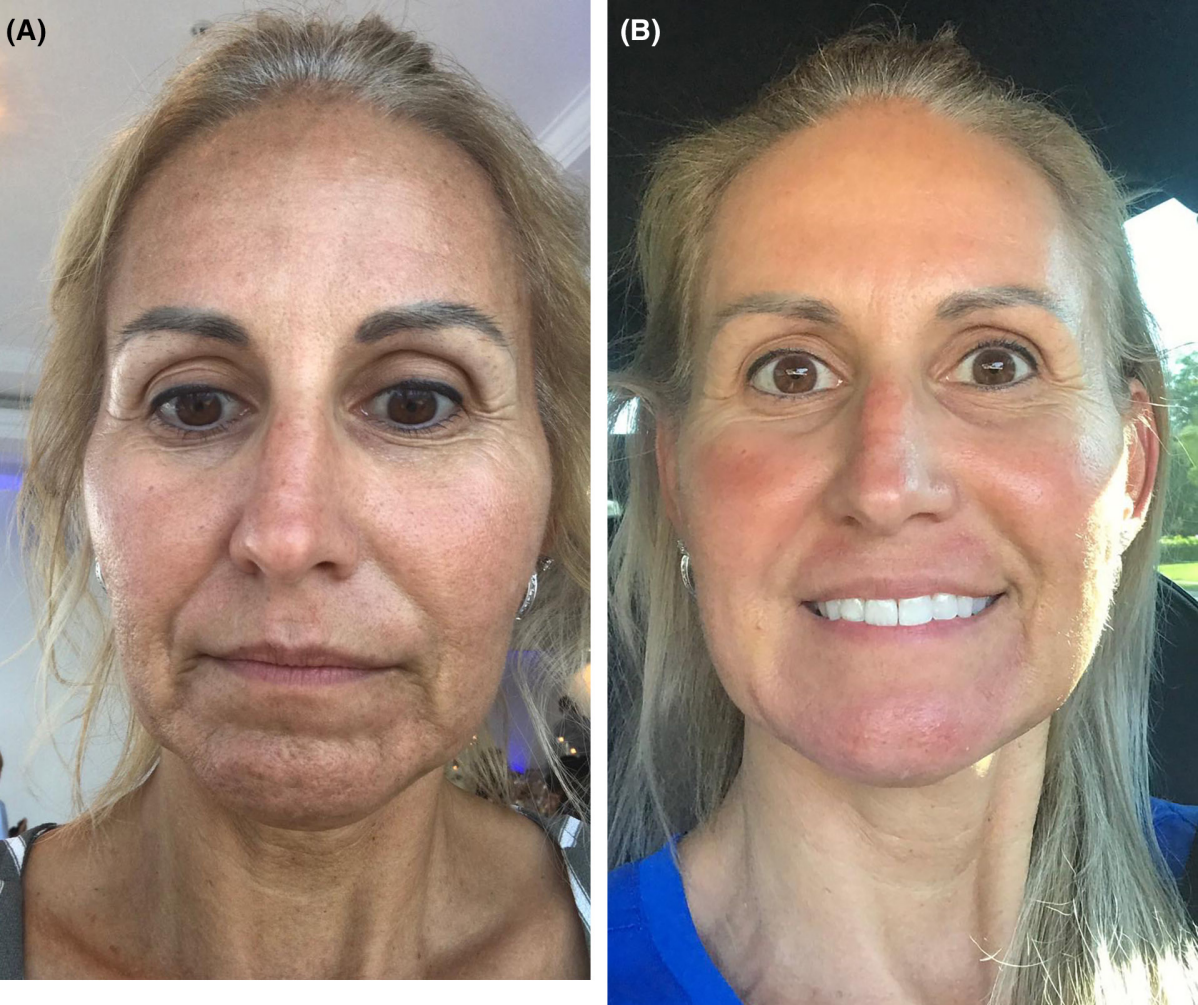
(A) Before and (B) after of a mid‐40 year‐old woman having received treatment for deep fine lines and wrinkles. Patient was treated with some Bio‐Filler injections. Case performed by Dr. Richard Miron.

The preliminary studies have demonstrated that the Bio‐Filler is a much longer lasting APC with up to 4–6 months resorption properties.^17^, ^18^, ^19^ Figure 23 demonstrates an example of a before and after of a mid 40‐year‐old woman having received Bio‐Filler injections. The Bio‐Filler is much thicker in consistency than liquid APCs as highlighted in QR Code 8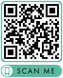. Further studies are needed to assess the frequency of injections, comparison to HA filler as well of the safety of the Bio‐Filler if accidentally injected into a vessel. Nevertheless, Bio‐Filler is a cost‐effective, safer, and effective esthetic process being introduced in esthetic medicine/dermatology. It works well for fine rhytides reduction and to volumize, contour, and rejuvenate the face, neck, and hands. The consistency and autologous nature of Bio‐Filler are well accepted by patients when compared to HA fillers.^154^

### 14.4 Clinical guidelines

**Table:** 

Tips for mixing and injecting ALB‐PRF: Eliminate bubbles before heating upper layer of PRF Ensure that heated albumin gel cools off before mixing with liquid. Use a firm luer to luer connection and mix slowly. Ensure no dead space in leur connection by pushing liquid through the connector and elimination bubbles before mixing. Use a 25 gauge needle or large bore cannula to inject Alb‐PRF to avoid blockages.

## 15 LASERS WITH APCs IN FACIAL ESTHETICS

### 15.1 Background

The use of lasers for facial esthetic procedures has seen a long history of use since the 1960s.^8^ While originally clinical procedures and indications were limited to ablative therapies, over the past decade widespread use has been observed owing to technological advancements. Today, over 150 commercially available lasers exist on the market for various indications including scar revisions, pigmented lesions, vascular lesions, hair removal, facial resurfacing, facial rejuvenation, fat ablation and laser lipolysis.^155^ This article does not aim to provide in‐depth knowledge on the topic but instead wishes to present uses of laser therapy in facial esthetics. Much like platelet concentrates, laser therapy offers an all‐natural regenerative strategies to facial tissues.^8^

While CO_2_ lasers were first utilized as extensive ablative therapies with long downtimes, modern developments of newer and more frequently utilized wavelengths have seen widespread. In the 1990s, the Erbium:YAG laser (Er:Yag) was introduced demonstrating a positive role in skin resurfacing, especially for mild skin pigmentation, facial wrinkles and acne scaring.^156^ Furthermore, their use as nonablative fractional lasers have been developed with much shorter recovery periods. The use of the Neodymium:YAG laser (Nd:Yag) is a deeper penetrating laser that may be utilized to stimulate tissue regeneration and or ablate/attracted to pigmented lesions. Several indications for laser therapies including resurfacing, laser peels, as well as age spot/mole/vein/hair removal.

### 15.2 Outcome

A total of 7 studies reviewed PRP^42^, ^157^, ^158^, ^159^, ^160^ and laser therapy for the treatment of acne scarring (Table 11). All studies created PRP using a 2‐spin centrifugation protocol. The studies demonstrated that the addition of PRP to ablative laser therapy improves acne scarring, patient satisfaction, and postprocedural symptoms. As fractional laser creates microthermal wounding of skin, PRP is added to aid in wound healing and promote scar resolution. Gawdat et al.^42^ demonstrated that topical application of PRP after laser may be just as effective as intradermal injections of activated PRP when utilized with fractional ablative laser treatment, with less pain in the topical group. Adverse effects, including erythema and oedema, were significant shorter in duration in the PRP‐treated groups, leading to shorter downtime (*p* = 0.02). Min et al.^162^ assessed changes in growth factors during treatment and concluded that increased levels of TGFb can be suggested as a mechanism for the clinical improvement shown when using a combination of fractional CO_2_ laser and PRP treatment.

**TABLE 11:** APC with laser ablation.

Author	Study type	Subjects Gender Age Condition % Smokers	Centrifuge rpm/min *g* force Anticoagulant	Treatment protocol and follow‐up	*Grading System* Outcome
PRP Microneedling for acne scars					
Faghihi et al.^157^	*NRCT* *spl‐f*	16 patients ♀ = 12/♂ = 4 Age: 22–52 years Acne gr2–4	PRP double spin 200 *g* 3 min 5000 *g* 5 min AC used Activator CaCl	Two sessions 1 month apart: Ablative CO_2_ laser the LHS Intradermal PRP vs. NS	FU 4 months after treatment PRP 87.5% of cases and with saline in 68.8% of cases (*p* = 0.23) Patients noted being satisfied or very satisfied with the PRP treatment in 56.2% of cases and with the saline treatment in 43.8% of cases (*p* = 0.12) Erythema, oedema and crusting SS lees PRP side Final conclusion: PRP combined with Laser is a better treatment option with shorter downtime
Lee et al.^158^	*RCT* Spl‐f	14 patients ♀ = 4/♂ = 10 Age: 21–38 years Acne mod sev	Prosys PRP system 3000 rpm 3 min 4000 rpm 3 min Not activated	Two sessions 1 month apart Q‐ray ablative fractional CO_2_ laser PRP injections_half face NS‐LHS	Degree of clinical improvement was significantly better on the PRP‐treated side (2.7) than on the saline‐treated side (2.3) (*p* = 0.03) Erythema, oedema and crusting SS lees PRP side Final conclusion: Treatment with PRP after ablative CO_2_ fractional resurfacing enhances recovery time and synergistically improves the clinical appearance of acne scarring
Gawdat et al.^42^	*RCT* *Spl‐f*	30 patients ♀ = 18/♂ = 12 Acne gr 2–4 Age: 19–35 years	Double spin 150 *g*—15 min 400 *g*—10 min AC: ACD Activator: CaCl	Three sessions 1 month apart. Fractional CO_2_ laser + Intradermal‐PRP RHS intradermal NS LHS Intradermal‐PRP RHS topical‐PRP LHS	FU 6 months Intradermal or topical PRP showed SS in skin smoothness > saline‐treated area (*p* = 0.03) NS diff between intradermal PRP and topical PRP (*p* = 10) In in areas treatedwith PRP, leading SS shorter downtime (*p* = 0.02) Final conclusion: PRP shorter downtime than FCL alone and better tolerability than FCL combined with ID PRP
Kar et al.^159^	*CCT* *Spl‐f*	30 patients ♀ = 10/♂ = 20 Acne gr 2–4 Age: 18–34 years	1500 rpm 10 min 3000 rpm 20 min Not activated	Three sessions 1 month apart. Fractional CO_2_ laser + Only RHS Fractional CO_2_ laser + Intradermal‐PRP LHS	FU 3 months *GQS* Appearance of scars SS improvement both sides (*p* = 0.0001) No diff between R and L face (*p* = 0.2891) Self assessment higher for both sides after Rx (*p* = 0.0001) Patients reported significantly decreased in‐Itensity of erythema, edema, and pain symptoms on the side treated with combination treatment including topical PRP compared with laser treatment alone (*p* \0.05) Final conclusion: Both methods were effective in management of acne scars. Addition of PRP did not improve the scar quality; however, the downtime and inflammation associated with laser treatment gets significantly reduced on the PRP‐treated side
Min et al.^160^	*RCT* *Spl‐f*	25 patients ♀ = 18/♂ = 12 Acne gr 2–4 Age: 24–34 years	160 *g* 10 min 400 *g* 10 min AC: ACD Activator CaCl	Two sessions 1 month apart Fractional CO_2_ laser LHS intradermal PRP, half face saline	FU day 84 The mean IGA scores showed that the fractional CO_2_ laser plus PRP resulted in an improvement of;75% vs. the 50% seen with a fractional CO_2_ laser plus saline (*p* \ 0.001) Final conclusion: ECCA scores showed significantly greater improvement with treatment using a CO_2_ laser plus PRP (*p* \0.05) degree of erythema was significantly lower on the PRP‐treated side than on the saline‐treated side throughout the whole study period (*p* \ 0.05)
Abdel et al.^161^	*RCT* *Spl‐f*	30 patients ♀ = 18/♂ = 12 Acne gr 2–4 Age: 20–46 years	3000 rpm 7 min 4000 rpm 5 min AC: sodium citrate Activator, CaCl	Two session 1 month apart Ablative CO_2_ laser PRP RHS face	FU 6 months GBS Combination better than the CO_2_ laser monotherapy (*p* < 0.001) Final conclusion: Patients were more satisfied with the combination treatment than with laser monotherapy (*p* \ 0.001)
Zhu et al.^162^	*RCT*	22 patients ♀ = 18/♂ = 12 Acne gr 2–4 Age: 19–39 years	1500 rpm 10 min 3000 rpm 20 min Activator: Ca Gluconate	Three sessions 1–2 months apart Erbium fractional laser PRP topical	FU 3 months Improvement was rated as moderate Physician assessment showed that 90.9% of patients reported excellent or marked improvement after three treatments; no patients showed no improvement Final conclusion: Patient assessment at 4 weeks after treatment completion showed that 91% were satisfied or very satisfied, with 45% wanting to receive further treatment. All six patients with active acne had resolution
Hui et al.^163^	*CT* *Spl‐f*	13 patients ♀ = 13/♂ = 0 Facial aging Age: 32–57 years	1200 rpm 10 min 3500 rpm 5 min AC: heparin calcium Activator: calcium gluconate	LHS PRP/NS Then CO_2_ laser	Combination superior to laser treatment alone *p* < 0.05 PRP group had faster recovery, less duration of adverse events Final conclusion: PRP and ultra‐pulsed fractional CO_2_ laser had a synergistic effect on the therapy for facial rejuvenation

Abbreviations: ECCA, Echelle d'evaluation clinique des cicatrices d'acne; TGF‐b, transforming growth factor‐b; TIMP, tissue inhibitor of metalloproteinase. Study type: CCT, controlled clinical trial; CS, case series; non‐R, nonrandomized; RCT, randomized controlled trial; spl‐s, split‐scalp. Centrifuge data: *g*, *g*‐force; rpm, revolutions/rotations per minute. APC preparation: AC, Anticoagulant; ACD, acid citrate dextrose; CaCl, calcium chloride. Treatment: N/S, Normal saline. Administration: HA, hyaluronic acid; ID, intradermal; MN, microneedling. Assessment: TTD, tear trough deformity; TTRS, Tear Trough Rating Scale; WSRS, Wrinkle Severity Rating Scale. Findings: NS, nonstatistically significant finding; SS, statistically significant finding.

Aside from scarring, lasers have also been used for skin rejuvenation. Fractional CO_2_ lasers are an efficient, precise, and safe therapeutic intervention for skin resurfacing.^163^ Hui et al. concluded that PRP combined with ultra‐pulsed fractional CO_2_ laser had a synergistic effect on facial rejuvenation, shortening duration of side effects, and promoting better therapeutic effect, as shown in a split face study, analyzed by VISIA.

### 15.3 Conclusion

Much like platelet concentrates, laser therapy offers an all‐natural regenerative strategies to facial tissues and has a synergistic effect with platelet concentrates. Laser peels in particular have become quite popular in the space of esthetic medicine (QR Code 9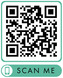). While both technologies are only beginning to be utilized in combination treatments, their combined use offers patients advanced approaches in the “all natural” facial esthetic domain. Future studies remain needed to further assess these approaches, especially granted the wide range of available lasers and wavelengths on the market. Noteworthy, all studies pointed to the fact that the use of APCs was shown to accelerate healing times.

### 15.4 Clinical recommendations

Because many laser wavelengths are ablative, the use of APCs prior to laser therapy has been shown to result in cell damage. Laser treatment is therefore performed first when combining modalities, with APCs applied directly after treatment either topically, via microneedling or as an injectable.

## 16 FUTURE APPLICATIONS OF APCs IN FACIAL ESTHETICS

**FIGURE 24:**
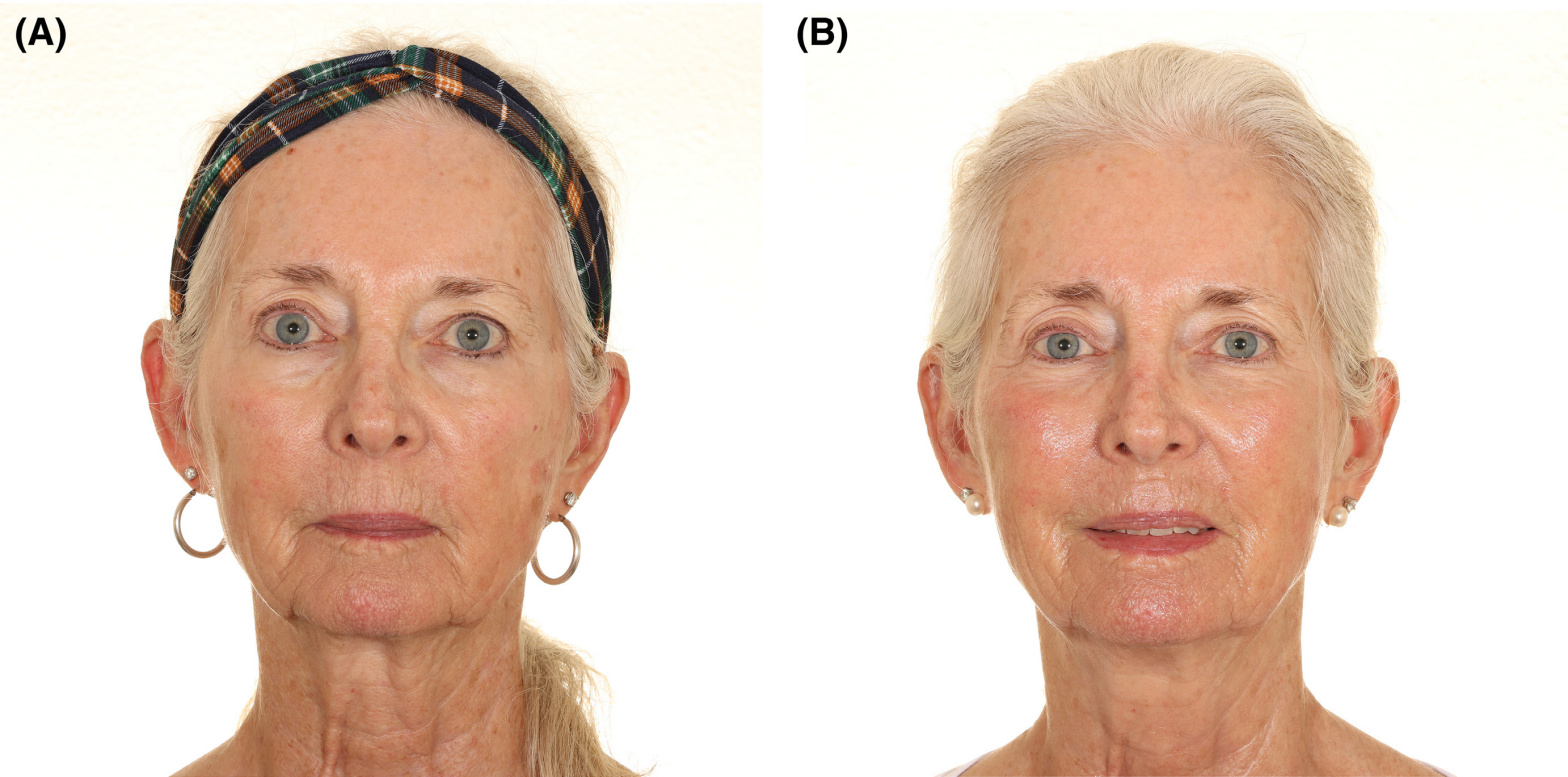
(A) Before and (B) after of a 72‐year‐old lady having received three treatments combining lasers and PRF (QR Code 10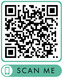). Case performed by Dr. Richard Miron.

In the field of facial esthetics, a wide array of small biomolecules have been utilized to favor skin regeneration and/or hair regeneration. Interestingly, it was recently proposed that liquid‐PRF could be combined with hyaluronic acid very simply, but could also be pre‐mixed with various vitamins, regenerative agents, fibroblast growth factors and a vast array of various small biomolecules.^164^ This field of research is sure to expand over the coming years as the ability to create a three‐dimensional fibrin mesh with entrapments of growth factors and small biological agents at the clinician's discretion will surely open a wide array of clinical possibilities in the coming years. Furthermore, comination treatments with exosomes, amino acids, vasodilators, vitamins and energy based devices are becoming more and more popular (Figure 24, QR Code 10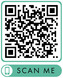). Once again, an array of research in the hair field is also surely set to explode in the coming years.

### 16.1 APCs for use in combination with HA filler and other modalities

An area that is currently being investigated is the possible synergistic effect of cross‐linked hyaluronic acid fillers (HA fillers) and APC injections on facial skin rejuvenation.^165^ Very little evidence exists about the efficacy of combining such procedures.

Cross‐linking agents can also be quickly introduced within the APC, which significantly enhances the degradation properties of APCs. As is currently done with HA fillers, the cross‐linking agents are, in fact, responsible for the more extended degradation properties in most utilized commercial fillers. Therefore, while APC currently only lasts a few months, it is also possible to extend their resorption properties significantly simply by adding cross‐linking agents, such as conventional HA fillers, which would be a tremendous benefit to the field as the treating clinician could (1) offer similar degradation properties as currently utilized fillers, (2) being more natural and having the inclusion of natural cells and growth factors that participate further with regeneration, (3) being markedly cheaper than currently available facial fillers on the market and (4) present more biocompatibility/safety thus reducing the change of adverse effects/complications such as allergic reactions and vascular occlusions. Each of these advantages poses great additional benefit to patient care.

An RCT by Hersant et al.,^56^ concluded that combining PRP and HA (a 50;50 mix), there was a benefit for facial rejuvenation with a highly significant improvement in facial appearance and skin elasticity compared with PRP or HA alone. A total of 93 patients were included. Treatment with Cellular Matrix led to a very significant improvement in the overall facial appearance compared with treatment with a‐PRP or HA alone (*p* < 0.0001). Participants treated with the cellular matrix showed a 20%, 24%, and 17% increase in FACE‐Q score at 1, 3, and 6 months posttreatment, respectively.

In summary, combining HA filler with APCs is a promising option for facial esthetic treatments with a number of studies evaluating their use.^166^ Furthermore this combination approach has been utilized for chronic ulcers effectively.^167^, ^168^, ^169^, ^170^ Further studies and systematic reviews are needed to determine the optimal ratios of the two, patient satisfaction with the volumizing effect as well as safety protocols to avoid vascular occlusion after injection.

### 16.2 APC with nanofat

Nanofat grafting is a fat transfer method in which adipose tissue is removed from a region rich in adipose tissue and then applied via a microinjection technique to a respective region such as the skin. This technique has become quite popular in recent years due to its rejuvenation properties but also owing to its all‐natural autologous sourcing.^171^ Combining the nano fat with APCs may enhance the process of patients undergoing facial rejuvenation treatments.

A large RCT study by Liang et al.^47^ compared 103 patients with skin aging who underwent both nano fat and intradermal liquid‐PRF injection (treatment group) with a control group of 128 patients who underwent hyaluronic acid (HA) injection treatment. The results of VISIA testing of facial skin condition showed that patients in each group achieved significant skin quality enhancement at 1 month (*p* < 0.01 vs. before treatment). The scores at 12 months were still significant in the treated group but not in the control group. It was concluded that nano fat + PRF injections are a safe, highly effective, and long‐lasting method for skin rejuvenation when injected intradermally.^47^

APCs, when used as an adjuvant to other facial esthetic modalities, have almost always a synergistic effect. PRP, when used with fractional photothermolysis, showed an overall positive effect with positive collagen induction. Benefits included improved healing time with improvements in symptoms such as erythema, edema, crusting, and clinical outcomes during rejuvenation. It has been shown to be a convenient treatment without serious complications.^172^ This is especially important in aging areas that pose some risk with other modalities such as around the eyes or in regions prone to potential vascular occlusions which may cause necrosis. When combined with laser therapies, microneedling, dermal fillers, and autologous fat grafting, APCs produce synergistic effects, leading to improved esthetic results.^173^, ^174^, ^175^, ^176^, ^177^

## 17 CONCLUSION: APCs IN FACIAL ESTHETICS

APCs have now been studied for well over a decade in facial esthetics for various conditions. They can be applied topically, via intradermal injection or via microneedling. They are used as either stand alone treatments or in combination with exisiting modalities, and can be administered in the form of liquid PRP and PRF or in the gel like albumin‐APC form (BioFiller). Strides have been made in the study of PRP for dermatological conditions yet important questions remain unanswered. These include PRP preparation methods, activation versus non activation, as well as dosing, timing, and frequency of PRP injections, and techniques for delivery (microneedling vs intradermal injections). To date, there are only a few RCTs involving PRF in facial aesthetic applications, however great potential is shown as in vitro studies have demonstrated an over 2‐fold improvement in collagen synthesis and the limited clinical studies have shown superior results including patient reported outcomes. Clinical studies however remain needed, avoiding the pitfalls of PRP study variability, preparation techniques, terminology, outcome measure, and modes of delivery. After comprehensive review of the literature, it appears that APCs have great potential in facial esthetics. They are safe, effective, low‐cost agents used routinely in esthetic medicine with much potential upside for practicing clinicians.

## CONFLICT OF INTEREST STATEMENT

Richard Miron is the founder of BioPRF, Florida USA.

## Data Availability

Data sharing not applicable to this article as no datasets were generated or analyzed during the current study.
